# A multivariable *cis*-Mendelian randomization method robust to weak instrument bias and horizontal pleiotropy bias

**DOI:** 10.1093/bib/bbaf250

**Published:** 2025-06-05

**Authors:** Yihe Yang, Noah Lorincz-Comi, Mengxuan Li, Xiaofeng Zhu

**Affiliations:** Department of Population and Quantitative Health Sciences, Case Western Reserve University School of Medicine, 10900 Euclid Ave, Cleveland, OH 44106, United States; Department of Population and Quantitative Health Sciences, Case Western Reserve University School of Medicine, 10900 Euclid Ave, Cleveland, OH 44106, United States; Department of Population and Quantitative Health Sciences, Case Western Reserve University School of Medicine, 10900 Euclid Ave, Cleveland, OH 44106, United States; Department of Population and Quantitative Health Sciences, Case Western Reserve University School of Medicine, 10900 Euclid Ave, Cleveland, OH 44106, United States

**Keywords:** cis-Mendelian randomization, horizontal pleiotropy, multivariable Mendelian randomization, weak instrument bias

## Abstract

Multivariable *cis*-Mendelian randomization (*cis*-MVMR) has become an effective approach for identifying therapeutic targets that influence disease susceptibility. However, biases from invalid instruments, such as weak instruments and horizontal pleiotropy, remain unsolved. In this paper, we propose a new method called the *cis*-Mendelian randomization bias correction estimating equation (*cis*-MRBEE), which mitigates weak instrument bias by leveraging a local sparse genetic architecture: most variants within a genomic region are associated with a trait through linkage disequilibrium with a few causal variants. *Cis*-MRBEE identifies causal variants or proxies of exposures via fine-mapping, re-estimates genetic associations using the identified variants, and applies a double-penalized minimization to estimate causal exposures and account for horizontal pleiotropic effects. Simulations showed that in the presence of weak instruments and horizontal pleiotropy, directly adapting standard MVMR methods to *cis*-MVMR was infeasible, and existing *cis*-MVMR methods failed to control type I errors. In contrast, *cis*-MRBEE exhibited robustness to these sources of bias. We applied *cis*-MRBEE to the *ANGPTL3* locus and identified a credible set comprising APOA1, APOC1, and PCSK9 as likely causal proteins for LDL-C, HDL-C, and TG. The subsequent analysis revealed a complex protein regulation network that influenced lipid traits. Furthermore, we used *cis*-MRBEE to discover that the expressions of *CR1* in the basal ganglia, hippocampus, and oligodendrocytes were potentially causal for Alzheimer’s disease and its biomarkers, A$\beta $42 and pTau, in cerebrospinal fluid.

## Introduction

Mendelian randomization (MR) is an instrumental variable (IV) method that leverages genetic variants as IVs to estimate the causal effects of exposures on outcomes [1]. Compared with randomized controlled trials (RCTs), MR depends on the natural randomization of genetic variation, thereby avoiding the ethical and logistical challenges often encountered in RCTs. In addition, MR can be conducted by only using genome-wide association studies (GWAS) summary data [2], which have significantly contributed to the flourishing of MR in genetic epidemiology. Beyond its role in causal inference, MR has inspired the development of statistical methods for broader applications, such as constructing phenotype networks [3] and detecting gene–environment interactions [4]. As a comprehensive approach, MR and its extensions have provided valuable insights into the etiology of numerous diseases in the post-GWAS era [5].

Conventional MR methods face significant challenges in the presence of weak instruments or horizontal pleiotropy. Specifically, MR relies on three key conditions called the valid IV conditions: (IV1) the genetic variants must be strongly associated with the exposure, (IV2) their association with the outcome must occur solely through the exposure, and (IV3) they must be independent of confounders of the exposure–outcome relationship [5]. Genetic variants that fail to meet (IV1) are referred to as weak instruments [6], while genetic variants that violate (IV2) or (IV3) are considered to be uncorrelated horizontal pleiotropy (UHP) or correlated horizontal pleiotropy, respectively [7]. A large number of statistical methods have been developed to improve conventional MR methods, making them robust to these sources of bias; see, e.g. MR-Median [8], MR-PRESSO [9], MR-RAPS [10], CAUSE [7], IMRP [11], MRcML [12], and MR.CUE [13].

*Cis*-Mendelian randomization (*cis*-MR) has garnered increasing attention for its potential in drug target discovery and validation [14]. This growing interest arises from its ability to use gene expressions and protein abundances as exposures, given that coding gene regions encode proteins, which often serve as drug targets [15]. *Cis*-MR typically uses genetic variants located near a gene of interest, known as *cis*-variants, as IVs to examine causal relationships. Since *cis*-variants are often in linkage disequilibrium (LD), a significant category of *cis*-MR methods has been developed to improve conventional MR approaches, making them robust to correlated IVs. Representative *cis*-MR methods include *cis*-IVW [16], JAM algorithm [17], and PC-GMM [18]. More recently, Yuan *et al*. [19] developed the MRAID, Lin and Pan [20] proposed the *cis*-MRcML, and van der Graaf *et al*. [21] contributed the MR-link 2, which can handle correlated IVs and horizontal pleiotropy, simultaneously.

Multivariable MR (MVMR), which considers multiple exposures simultaneously, offers a compelling solution to estimate the direct causal effects of exposures on an outcome [22]. By contrast, univariable MR and *cis*-MR, referred to as UVMR and *cis*-UVMR, respectively, estimate the total causal effect of an exposure [22]. Recent developments in MVMR methods include MVIVW [8], GRAPPLE [23], MVMRcML [24], SRIVW [25], and MRBEE [26]. Furthermore, multiple methodological contributions have been made to multivariable *cis*-MR (*cis*-MVMR). For instance, Burgess *et al*. [16] proposed the *cis*-MVIVW to account for correlated IVs, Batool *et al*. [27] extended the PC-GMM and Patel *et al*. [28] further modified it to address overdispersion heterogeneity, Cai and Dudbridge [29] developed the CWBLS to adjust for collider bias, and Lorincz-Comi *et al*. [30] contributed a software called whole-genome regulatory network analysis tool (HORNET) for genome-wide causal genes searches.

However, most *cis*-UVMR and *cis*-MVMR methods do not consider a unique feature which we call the “sparse genetic architecture”: within a genomic region, only a few variants are truly causal for a trait, while the remaining variants are associated with the trait due to LD with these causal variants. In this case, simply relaxing the threshold of $r^{2}$ in clumping and thresholding (C+T) method [31] to select IVs would result in most IVs being null, which would only introduce estimation error. Especially, many regulatory quantitative trait loci (xQTL) datasets, such as the genotype-tissue expression (GTEx) [32], typically have a sample size of a few hundred individuals. The IVs selected based on small sample sizes often contain substantial estimation errors and thus lead to large weak instrument bias. To address this issue, Yuan *et al*. [19] proposed a *cis*-UVMR method called MRAID, which applies a spike-and-slab prior distribution on the effects of IVs. This prior distribution performs Bayesian fine-mapping to identify significant IVs with nonzero effects, thereby reducing estimation errors. However, how to leverage the sparse genetic architecture for *cis*-MVMR to address weak instrument and horizontal pleiotropy biases simultaneously remains an unresolved challenge.

In this paper, we propose an extension of MRBEE that we named *cis*-MRBEE for *cis*-MVMR analysis. *Cis*-MRBEE uses fine-mapping methods, such as the sum of single effects (SuSiE) [33, 34], to first identify a subset of variants potentially causally associated with exposures. We call this small set of variants as “informative” variants, distinct from the actual causal variants. Subsequently, *cis*-MRBEE takes a new strategy called “sparse prediction,” which uses the informative variants to predict the GWAS effect sizes. It limits estimation errors to just a few variants selected by the fine-mapping method instead of all variants in the region, thus improving the accuracy of MRBEE. Moreover, *cis*-MRBEE employs a double-regularized minimization to simultaneously select causal exposures and horizontal pleiotropy.

Simulations demonstrated that *cis*-MRBEE yielded unbiased causal effect estimates, exhibited high statistical power, and effectively controlled type-I error rates. We applied *cis*-MRBEE to two real datasets. First, on the *ANGPTL3* locus, *cis*-MRBEE identified a credible set comprising APOA1, APOC1, and PCSK9 as causal proteins influencing low-density lipoprotein cholesterol (LDL-C), high-density lipoprotein cholesterol (HDL-C), and triglycerides (TG), using protein QTLs (pQTLs) as instruments. The subsequent analysis further revealed a complex protein regulation network that impacts lipid traits. Second, *cis*-MRBEE identified *CR1* expression in the basal ganglia, hippocampus, and oligodendrocytes as potentially causal for Alzheimer’s disease (AD), amyloid-beta-42 levels (A$\beta $42), and phosphorylated tau (pTau) levels in cerebrospinal fluid (CSF), utilizing both expression QTLs (eQTLs) and single-cell eQTLs (sceQTLs) as instruments.

## Method

### Cis-MVMR model

Consider the following *cis*-MVMR model:


(1) \begin{align*}\kern-.5pc y_{i}&=\sum_{j=1}^{p}x_{ij}\theta_{j}+\sum_{s=1}^{m}g_{is}\gamma_{s}+\epsilon_{i0}, \end{align*}

(2) \begin{align*} x_{ij}&=\sum_{s=1}^{m}g_{is}\beta_{sj}+\varepsilon_{ij},\quad 1\leq j\leq p, \end{align*}

where $y_{i}$ is an outcome, $\boldsymbol x_{i}=(x_{i1},\dots ,x_{ip})^\top $ is a vector of the $p$ exposures, $\boldsymbol g_{i}=(g_{i1},\dots ,g_{im})^\top $ is a vector of the $m$ IVs, $\boldsymbol \beta _{j}=(\beta _{1j},\dots ,\beta _{mj})^\top $ is a vector of the corresponding genetic effects for the $j$th exposures, $\boldsymbol \gamma =(\gamma _{1},\dots ,\gamma _{m})^\top $ refers to the horizontal pleiotropy or the direct genetic effects on the outcome, $\boldsymbol \theta =(\theta _{1},\dots ,\theta _{p})^\top $ is a vector of the causal effects of the exposures on the outcome, and $\boldsymbol \epsilon _{i}=(\epsilon _{i0},\varepsilon _{i1},\dots ,\varepsilon _{ip})^\top $ are the white noises. In addition, the means and variances of $y_{i}$, $x_{i1},\dots ,x_{ip}$, and $g_{i1},\dots ,g_{im}$ are assumed to be 0 and 1, respectively, cov$(\boldsymbol g_{i})=\mathbf R$ is the LD matrix of IVs, cov$(\boldsymbol \epsilon _{i})=\mathbf \Sigma _\varepsilon $ is the covariance matrix of noises, and $\boldsymbol g_{i}$ and $\boldsymbol \epsilon _{i}$ are independent of each other. Substituting for $\boldsymbol x_{i}$ in (1), we thus obtain the following reduced form:


(3) \begin{align*} y_{i}=\sum_{s=1}^{m}g_{is}\alpha_{s}+\varepsilon_{i0},\end{align*}

where


(4) \begin{align*} \alpha_{s}=\sum_{j=1}^{p}\theta_{j}\beta_{sj}+\gamma_{s},\quad\varepsilon_{i0}=\sum_{j=1}^{p}\varepsilon_{ij}\theta_{j}+\epsilon_{i0}.\end{align*}

Current GWAS summary statistics, which represent marginal rather than direct causal genetic effects, have facilitated the development of many novel statistical methods [2]. Specifically, the GWAS summary data are generated as follows. Let $\mathbf y^{[0]}=(y^{[0]}_{1},\dots ,y^{[0]}_{n_{0}})^\top $ be the sample vector from an outcome GWAS, $\boldsymbol x^{[1]}=(x^{[1]}_{1},\dots ,x^{[1]}_{n_{1}})^\top ,\dots ,\boldsymbol x^{[p]}=(x^{[p]}_{1},\dots ,x^{[p]}_{n_{p}})^\top $ be the sample vectors from $p$ exposure GWAS cohorts, and $\boldsymbol g^{[0]}=(g^{[0]}_{is})_{n_{0}\times m},\dots ,\boldsymbol g^{[p]}=(g^{[p]}_{is})_{n_{p}\times m}$ be the sample matrices of $m$ genetic variants from the outcome and $p$ exposures, and $n_{0},n_{1},\dots ,n_{p}$ are the corresponding sample sizes, respectively. The marginal genetic effect between a variant and an exposure/outcome is estimated by


(5) \begin{align*} \hat a_{s}=\frac{\boldsymbol g_{s}^{[0]\top}\boldsymbol y^{[0]}}{n_{0}},\quad \hat{b}_{js}=\frac{\boldsymbol g_{s}^{[j]\top}\boldsymbol x^{[j]}}{n_{j}}.\end{align*}

GWAS summary data usually include $\hat{\boldsymbol a}=(\hat a_{1},\dots ,\hat a_{m})^\top $, $\hat{\boldsymbol b}_{j}=(\hat b_{1j},\dots ,\hat b_{mj})^\top $, $j=1,\dots ,p$, the their corresponding SEs, $P$-values, sample sizes, and SNPs information. Furthermore, to let var($y_{i})=1$, var$(x_{ij})=1$ ($1\leq j \leq p$), and $\text{var}(g_{is})=1$ ($1\leq s\leq m$), the GWAS summary data are usually standardized before statistical analysis:


(6) \begin{align*} \hat a_{s}\leftarrow\frac{\hat a_{s}}{\text{se}(\hat a_{s}-a_{s})\sqrt{n_{0}}},\quad \text{se}(\hat a_{s}-a_{s})\leftarrow\frac1{\sqrt{n_{0}}},\notag\\ \hat b_{sj}\leftarrow\frac{\hat b_{sj}}{\text{se}(\hat b_{sj}-b_{sj})\sqrt{n_{j}}},\quad \text{se}(\hat b_{sj}-b_{sj})\leftarrow\frac1{\sqrt{n_{j}}}.\end{align*}

Zhu and Stephens [35] pointed out that the summary statistics follow


(7) \begin{align*} \hat{\boldsymbol a}&\sim\mathscr{N}\left(\mathbf R\left(\sum_{j=1}^{p}\boldsymbol\beta_{j}\theta_{j}+\boldsymbol\gamma\right),\sigma_\alpha^{2}\mathbf R\right), \end{align*}

(8) \begin{align*} \hat{\boldsymbol b}_{j}&\sim\mathscr{N}\left(\mathbf R\boldsymbol\beta_{j},\sigma^{2}_{\beta_{j}}\mathbf R\right),\quad 1\leq j\leq p, \end{align*}

where $\sigma _\alpha ^{2}=n_{0}^{-1},\sigma ^{2}_{\beta _{j}}=n_{j}^{-1}$, $j=1,\dots ,p$, for standardized GWAS summary data. From this asymptotic distribution, it is easy to see that the summary statistic of a variant can be interpreted as the sum of the contributions from all causal variants being in LD with the variant.

### Sparse prediction of xQTL effect sizes

In this paper, we extended MRBEE [26] developed by our group to *cis*-MVMR. Specifically, MV-IVW [8], a fundamental method in MVMR, estimates $\boldsymbol \theta $ by


(9) \begin{align*} \hat{\boldsymbol\theta}_{\text{IVW}}=\arg\min_{\boldsymbol\theta}\left\{\frac12||\hat{\boldsymbol a}-\hat{\mathbf B}\boldsymbol\theta||_{2}^{2}\right\},\end{align*}

where $\hat{\mathbf B}=(\hat{\boldsymbol b}_{1},\dots ,\hat{\boldsymbol b}_{p})$ and $\mathbf R$ is assumed to be the identity matrix. The score function of this minimization is $\text{U}_{\text{IVW}}(\boldsymbol \theta )=-\hat{\mathbf B}^\top (\hat{\boldsymbol a}-\hat{\mathbf B}\boldsymbol \theta )$, which means that $\hat{\boldsymbol \theta }_{\text{IVW}}$ is yielded such that $\text{U}_{\text{IVW}}(\hat{\boldsymbol \theta }_{\text{IVW}})=0$. Since $\hat{\mathbf B}$ are estimated and so contains estimation error, $\hat{\boldsymbol \theta }_{\text{IVW}}$ is subject to the measurement error bias [36]. Lorincz-Comi *et al*. [26] thus pointed out that weak instrument bias originates from estimation errors in the GWAS effect estimates of exposures, and the severity of this bias is influenced by the relative size of the true genetic effect compared with the estimation error. To remove the estimation error bias, Lorincz-Comi *et al*. [26] proposed MRBEE that estimates $\boldsymbol \theta $ by using the following bias-corrected estimating equation:


(10) \begin{align*} \text{U}_{\text{BEE}}(\boldsymbol\theta)=\text{U}_{\text{IVW}}(\boldsymbol\theta)-\text{E}(\text{U}_{\text{IVW}}(\boldsymbol\theta)),\end{align*}

where


(11) \begin{align*} \text{E}(\text{U}_{\text{IVW}}(\boldsymbol\theta))=\text{E}\left((\hat{\mathbf B}-\mathbf B)^\top(\hat{\mathbf B}-\mathbf B)\boldsymbol\theta-(\hat{\mathbf B}-\mathbf B)^\top(\hat{\boldsymbol a}-\boldsymbol a)\right).\end{align*}

This bias-correction term in the equation (10) can be estimated from insignificant variants in GWAS [37].

**Figure 1:**
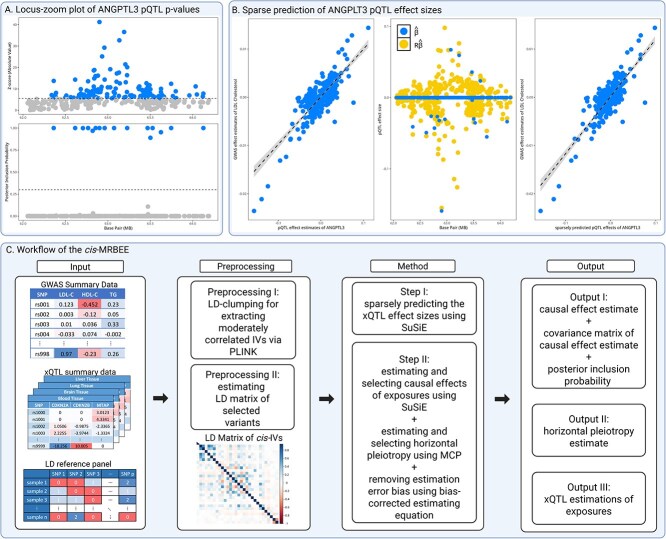
Overview of the *cis*-MRBEE workflow and key features. A. The locus-zoom plot with absolute Z-score for ANGPTL3 pQTLs, with fine-mapping performed using SuSiE. The gray and blue points refer to the variants with PIPs < 0.5 and otherwise, respectively. B. The sparse prediction of ANGPTL3 pQTL effect sizes highlights the reduction in estimation errors by selecting only the informative variants. The left subpanel shows a scatter plot of ANGPTL3 pQTL effect sizes and LDL-C GWAS estimates, with the dashed line and confidence interval indicating their regression relationship. The middle subpanel presents the direct effect of ANGPTL3 estimated by SuSiE (denoted as $\hat{\boldsymbol \beta }$, identified with PIP $>0.3$), along with sparsely predicted pQTL effect sizes (i.e. $\mathbf{R}\hat{\boldsymbol \beta }$). The marginal pQTL effect sizes in a region are largely driven by LD with a few informative variants. The right subpanel shows that using sparsely predicted pQTL effects provides a better prediction of local variation in LDL-C. C. the *cis*-MRBEE workflow. The inputs for *cis*-MRBEE include GWAS summary data and xQTL summary data. The *cis*-MRBEE method consists of two main steps: the sparse prediction of xQTL effect sizes and the joint estimation of causal effects and horizontal pleiotropy. The outputs include causal effect estimates, covariance matrices, PIPs, and horizontal pleiotropy estimates. Created in BioRender. Yang, Y. (2025) https://BioRender.com/kceg3g1.

However, most of the MVMR approaches that rely on the techniques of measurement error analysis [36] have a common limitation: they usually yield causal effect estimates with large SEs when the estimation errors are substantial [25]. The reason is the bias-variance tradeoff: while reducing or eliminating bias, these methods inevitably increase variance to a certain degree. This issue is particularly pronounced in *cis*-MVMR because most IVs do not have direct effects on exposure after accounting for their LD with informative variants, and meanwhile, the sample sizes for some xQTL cohorts (e.g. GTEx) are relatively small. Figure 1A presents an example, where the top panel presents the absolute values of Z-scores for the pQTLs effect sizes of ANGPLT3, while the bottom panel shows the corresponding posterior inclusion probabilities (PIPs) of being informative, as estimated by SuSiE [33]. Figure 1A indicates that, although many variants are associated with ANGPTL3, the number of informative variants is small.

To effectively remove the estimation errors in effect size estimates of exposures, *cis*-MRBEE estimates $\boldsymbol \beta _{j}$ by a penalized least square (PLS):


(12) \begin{align*} \hat{\boldsymbol\beta}_{j}^{\text{PLS}}=\arg\min_{\boldsymbol\beta_{j}\in\mathbb R^{m}}\left\{ \frac12\boldsymbol\beta_{j}^\top\mathbf R\boldsymbol\beta_{j}-\hat{\boldsymbol b}_{j}^\top\boldsymbol\beta_{j}+p_{\boldsymbol\lambda}(\boldsymbol\beta_{j}) \right\},\end{align*}

where $p_{\boldsymbol \lambda }(\cdot )$ is a penalty function with a vector of involved parameters $\boldsymbol \lambda $. Traditional variable selection penalties include lasso [38] and nonconvex penalties, such as those in the class encompassing smoothly clipped absolute deviation [39] and minimax concave penalty (MCP) [40]. On the other hand, from the Bayesian point of view, $p_{\boldsymbol \lambda }(\boldsymbol \beta _{j})\propto -\log f_{\boldsymbol \lambda }(\boldsymbol \beta _{j})$ where $f_{\boldsymbol \lambda }(\boldsymbol \beta _{j})$ is the density function of the prior distribution of $\boldsymbol \beta _{j}$. Bondell and Reich [41], Bas *et al*. [42], and Tang *et al*. [43] have shown that many Bayesian priors, including the global-local shrinkage priors and spiked-and-slab priors, can be viewed as special cases of nonconvex penalties.

In *cis*-MRBEE, SuSiE is used to select informative variants in the optimization (12), which is one of the most popular fine-mapping methods for GWAS summary data. Specifically, SuSiE constructs a novel prior distribution for $\boldsymbol \beta _{j}$, based on the assumption of “sum of single effects [33].” Notably, SuSiE estimates $\boldsymbol \beta _{j}$ via an iterative Bayesian stepwise selection (IBSS), rather than directly operating on the density function $f_{\boldsymbol \lambda }(\boldsymbol \beta _{j})$, although we use equation (12) to describe the principle in general. In addition, variants are considered as informative if they are in a 95 % credible set constructed by SuSiE. In Supplementary Materials, we show more details of the statistical principle and implementation of the sparse prediction.

Note that we can rearrange the equation (7) as


(13) \begin{align*} \hat{\boldsymbol a}&=\sum_{j=1}^{p}\hat{\boldsymbol b}_{j}\theta_{j}+\underbrace{\sum_{j=1}^{p}(\boldsymbol b_{j}-\hat{\boldsymbol b}_{j})\theta_{j}}_{\text{Estimation Error I}}+\mathbf R\boldsymbol\gamma+\boldsymbol e_\alpha\notag\\ &=\sum_{j=1}^{p}\hat{\boldsymbol b}_{j}^{\text{PLS}}\theta_{j}+\underbrace{\sum_{j=1}^{p}(\boldsymbol b_{j}-\hat{\boldsymbol b}_{j}^{\text{PLS}})\theta_{j}}_{\text{Estimation Error II}}+\mathbf R\boldsymbol\gamma+\boldsymbol e_\alpha,\end{align*}

where $\boldsymbol b_{j}=\mathbf R\boldsymbol \beta _{j}$ is the expectation of marginal GWAS effects, $\hat{\boldsymbol b}_{j}^{\text{PLS}}=\mathbf R\hat{\boldsymbol \beta }_{j}^{\text{PLS}}$ is termed the sparse prediction of $\boldsymbol b_{j}$, and the noise $\boldsymbol e_\alpha \sim \mathscr N(\mathbf 0,\sigma _\alpha ^{2}\mathbf R)$. Since $\hat{\boldsymbol{\beta }}_{j}^{\text{PLS}}$ is a sparse vector, with only the informative variants of the $j$th exposure having nonzero effects, the estimation errors in the sparsely predicted GWAS effect $\hat{\boldsymbol{b}}_{j}^{\text{PLS}}$ are primarily driven by these nonzero effect estimates. We provided a rigorous proof that shows, under moderate conditions, the expectation of Estimation II is theoretically smaller than that of Estimation I (Supplementary Materials). In this theoretical investigation, we constructed the asymptotic normal distribution of $\hat{\boldsymbol \beta }_{j}^{\text{PLS}}$ based on Theorem 2 (Oracle Property) in Fan and Li [39]. Also, our simulations and real data analysis suggest that Estimation Error II is much smaller than Estimation Error I in the equation (13), indicating that sparse prediction can efficiently reduce estimation errors (Supplementary Materials). While sparse prediction has been widely employed in transcriptome-wide association studies (TWAS) [44, 45] and also in MRAID [19], most of *cis*-MVMR methods have not considered it yet.

**Figure 2:**
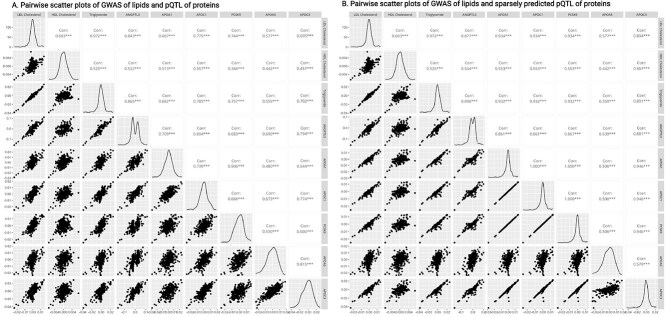
Original GWAS effects versus sparsely predicted GWAS effects. A. The scatter plots and empirical correlations between outcome GWAS effects and proteins’ pQTL effects from the *ANGPTL3* analysis. B. Similar scatter plot but uses sparsely predicted pQTL effects. Kernel density estimates are shown along the diagonal. The correlations do not account for LD. The analysis focuses on the *ANGPTL3* locus, while other proteins have *trans*-pQTLs at this locus. We manually reviewed whether common lipid-associated genes had *trans*-pQTLs in this region, retaining these six selected proteins. Created in BioRender. Yang, Y. (2025) https://BioRender.com/c8ae1o6.

Figure 1B demonstrates the sparse prediction of ANGPTL3 pQTL effect sizes, i.e. using the direct effects on ANGPTL3 estimated by SuSiE to predict the pQTL marginal effect sizes. It can be observed that the marginal pQTL effect sizes in a region are largely driven by LD with a few informative variants colored in blue. Figure 2 extends Fig. 1B to lipid traits and proteins analyzed in the real data: Fig. 2A presents the pairwise scatter plots and empirical correlation estimates between outcome GWAS effects and proteins’ pQTL effects at the *ANGPTL3* locus, while Fig. 2B shows the corresponding results for sparsely predicted pQTL effects. Clearly, the sparsely predicted effects had stronger correlations with outcome GWAS effects by reducing estimation errors (e.g. increasing from 0.757 to 0.944 for LDL-C and APOA1), and exhibiting more distinct horizontal pleiotropy patterns (e.g. sparsely predicted APOC3 pQTL effects).

### Cis-MRBEE

*Cis*-MRBEE estimates the causal effect $\boldsymbol \theta $ and the horizontal pleiotropy $\boldsymbol \gamma $ by


(14) \begin{align*} (\hat{\boldsymbol\theta}^\top,\hat{\boldsymbol\gamma}^\top)^\top=\arg&\min_{\boldsymbol\theta\in\mathbb R^{p},\boldsymbol\gamma\in\mathbb R^{m}}\left\{\frac12\left(\hat{\boldsymbol a}-\widehat{\mathbf B}^{\text{PLS}}\boldsymbol\theta-\mathbf R\boldsymbol\gamma\right)^\top\mathbf R^{-1}\notag\right.\\ &\left.\left(\hat{\boldsymbol a}-\widehat{\mathbf B}^{\text{PLS}}\boldsymbol\theta-\mathbf R\boldsymbol\gamma\right)+p_{\boldsymbol\lambda_\theta}(\boldsymbol\theta)+q_{\boldsymbol\lambda_\gamma}(\boldsymbol\gamma)\right\},\end{align*}

where $\widehat{\mathbf B}^{\text{PLS}}=(\mathbf R\hat{\boldsymbol \beta }_{1}^{\text{PLS}},\dots ,\mathbf R\hat{\boldsymbol \beta }_{p}^{\text{PLS}})$ is the matrix of sparsely predicted GWAS effects, $p_{\boldsymbol \lambda _\theta }(\cdot )$ and $q_{\boldsymbol \lambda _\gamma }(\cdot )$ are two penalty functions with parameters $\boldsymbol \lambda _\theta $ and $\boldsymbol \lambda _\gamma $. SuSiE is used to select the nonzero elements of $\boldsymbol \theta $, while MCP [40] is used for penalizing $\boldsymbol \gamma $. We select the exposures if they are in a 95% credible set constructed by SuSiE. In addition, we choose MCP for updating $\boldsymbol \gamma $ due to its analytical solution, which significantly reduces the computational burden, particularly when the number of IVs ($m$) is large. In fact, the purpose that we introduce $\boldsymbol \gamma $ is to control for large pleiotropic effects, ensuring more accurate causal effect estimation, rather than fine-mapping the non-exposure-mediated variants for the outcome. Therefore, since SuSiE is more computationally expensive than MCP, we prioritize a tradeoff between computational efficiency and estimation accuracy when estimating $\boldsymbol \gamma $. In Supplemental Materials, we demonstrate the two penalties, explanation of the algorithms, tuning parameter selection, covariance matrix estimation, hypothesis testing, and other implementation details.

Two additional innovation points of *cis*-MRBEE are highlighted as follows. First, we incorporate the unbiased estimating function into the above estimating procedure by using the same idea as the MRBEE (i.e. the equation (10)). Let $\boldsymbol \theta ^{(t)}$ and $\boldsymbol \gamma ^{(t)}$ are the estimates of $\boldsymbol \theta $ and $\boldsymbol \gamma $ at the $t$th iteration, and $\mathscr M_\theta ^{(t)}=\{j:\ \theta _{j}^{(t)}\neq 0\}$ and $\mathscr M_\gamma ^{(t)}=\{s:\ \gamma _{s}^{(t)}\neq 0\}$ be two working active sets. The marginal score function of $\boldsymbol \theta _{\mathscr M_\theta ^{(t)}}^{(t)}$ is thus given by


(15) \begin{align*} \text{U}\left(\boldsymbol\theta_{\mathscr M_\theta^{(t)}}^{(t)}\right)=&-\widehat{\mathbf B}_{\mathscr M_\theta^{(t)}}^{\text{PLS}\top}\mathbf R^{-1}\left(\hat{\boldsymbol a}-\widehat{\mathbf B}_{\mathscr M_\theta^{(t)}}^{\text{PLS}}\boldsymbol\theta_{\mathscr M_\theta^{(t)}}^{(t)}\notag\right.\\ &\left.-\mathbf R_{\mathscr M_\gamma^{(t)}}\mathbf R_{\mathscr M_\gamma^{(t)}\mathscr M_\gamma^{(t)}}^{-1}\mathbf I_{\mathscr M_\gamma^{(t)}}^\top\left(\hat{\boldsymbol a}-\widehat{\mathbf B}_{\mathscr M_\theta^{(t)}}^{\text{PLS}}\boldsymbol\theta_{\mathscr M_\theta^{(t)}}^{(t)}\right)\right),\end{align*}

where $\widehat{\mathbf B}^{\text{PLS}}_{\mathscr M_\theta ^{(t)}}$ and $\mathbf I_{\mathscr M_\gamma ^{(t)}}$ are the sub-matrices of $\widehat{\mathbf B}^{\text{PLS}}$ and $\mathbf I_{m}$ with columns being $\mathscr M_\theta ^{(t)}$ and $\mathscr M_\gamma ^{(t)}$, respectively. Similar to traditional MRBEE, if the expectation of $\text{U}(\boldsymbol \theta _{\mathscr M_\theta ^{(t)}}^{(t)})$ is not zero, the corresponding estimate of $\boldsymbol \theta _{\mathscr M_\theta ^{(t)}}^{(t)}$ such that $\text{U}(\boldsymbol \theta _{\mathscr M_\theta ^{(t)}}^{(t)})=\mathbf 0$ is subject to bias. We thus considered the following unbiased estimating equation:


(16) \begin{align*} \text{U}_{\text{BEE}}\left(\boldsymbol\theta_{\mathscr M_\theta^{(t)}}^{(t)}\right)=\text{U}\left(\boldsymbol\theta_{\mathscr M_\theta^{(t)}}^{(t)}\right)-\text{E}\left(\text{U}\left(\boldsymbol\theta_{\mathscr M_\theta^{(t)}}^{(t)}\right)\right),\end{align*}

and then re-update the sub-vector $\boldsymbol \theta _{\mathscr M_\theta ^{(t)}}^{(t)}$ by solving $\text{U}_{\text{BEE}}(\boldsymbol \theta _{\mathscr M_\theta ^{(t)}}^{(t)})=\mathbf 0$. The complete deduction can be found in Supplementary Materials.

Second, using SuSiE for sparse prediction (i.e. the minimization (12)) can lead to highly correlated or even identical predicted exposures. This occurs when the sample sizes of the xQTL studies are small, or a specific local genetic architecture presents, such as there is only one xQTL at the locus, making them statistically indistinguishable. An example is shown in Fig. 2B, which illustrates the identical predicted pQTL effects of APOA1, APOC, and PCSK9. The unbiased update (16) still follows a formula similar to the ordinary least squares estimate, which is susceptible to colinearity and fails to incorporate the information provided by the credible sets identified by SuSiE. To address this problem, we introduce the following discrete differential penalty:


(17) \begin{align*} d_{\lambda_{s}}(\text{CS}_{s})=\frac{\lambda_{s}}{2}\sum_{j,t\in\text{CS}_{s}}\left(\sqrt{\hat{\boldsymbol\beta}_{j}^{\text{PLS}\top}\mathbf R\hat{\boldsymbol\beta}_{j}^{\text{PLS}}\theta_{j}^{2}}-\sqrt{\hat{\boldsymbol\beta}_{t}^{\text{PLS}\top}\mathbf R\hat{\boldsymbol\beta}_{t}^{\text{PLS}}\theta_{t}^{2}}\right)^{2},\end{align*}

where $\text{CS}_{s}$ is the $s$th credible set given by SuSiE and $\lambda _{s}$ is a tuning parameter. This penalty function constrains the pairwise differences of the square root of the local heritability of outcome explained by exposures within a credible set, effectively mimicking the concept of single effects by enforcing averaged predictions across exposures in the credible set. As a result, $\hat{\boldsymbol \beta }_{j}^{\text{PLS}}\theta _{j}$ will be almost identical for all $j$ within the same credible set and hence can be appropriately regarded as a single effect. In addition, adding this penalty can also render the design matrix non-singular, thereby addressing collinearity.

In practice, *cis*-MVMR methods generally apply C+T with a more relaxed LD threshold to derive a set of IVs that are associated with at least one exposure; see, e.g. Gkatzionis *et al*. [14]. Although *cis*-MRBEE is designed to handle LD among IVs, we still recommend applying C+T, such as using --r2 0.64 in PLINK [31], that restricts the pairwise correlations of IVs within (−0.8,0.8) and remove highly correlated IVs. This precaution is necessary because *cis*-MRBEE relies on $\mathbf{R}^{-1}$, which becomes unstable when IVs are highly correlated, and potentially causes *cis*-MRBEE to fail. Empirical evidence suggests that while C+T may exclude some biologically causal variants, the remaining variants passing C+T can still include informative proxies for these causal variants, thereby preserving the model’s predictive power (Supplementary Materials). Note that this is a common problem for summarized-statistics-based methods. SuSiE [34] mitigates this problem by adding a small constant to the diagonal of $\mathbf{R}$. This adjustment is equivalent to incorporating a ridge penalty during fine-mapping, which could also bias the parameter estimation and model selection. How to thoroughly resolve the issue of $\mathbf{R}$ being unreliable when IVs are highly correlated warrants future investigation. In addition, we recommend including all variants passing the C+T filtering as IVs, rather than using only the informative ones selected by SuSiE. During the exploratory stage of method development, we observed that the latter approach could increase the variability of causal effect estimation.

## Results

### Simulation

We compared the performance of *cis*-MRBEE with six other methods. *Cis*-MVIVW [16] and PC-GMM [27, 28] were included for the comparison because they are frequently used in *cis*-MVMR. We also considered MRBEE-IPOD, a generalization of MRBEE used in HORNET [30] as a causal effect estimator, allowing for correlated IVs and using the iterative penalized outlier detection (IPOD) algorithm [46] to detect horizontal pleiotropy. The differences between *cis*-MRBEE and MRBEE-IPOD are: (1) *cis*-MRBEE further sparsely predicts the xQTL effect sizes, (2) uses SuSiE to select exposures, (3) leverages the credible sets identified by SuSiE with a differential penalty. We will demonstrate that *cis*-MRBEE has significant improvement over MRBEE-IPOD in simulations. We further included an adjusted *cis*-MVIVW, which applies the *cis*-MVIVW method with sparsely predicted GWAS effects as input. This could investigate whether other *cis*-MVMR methods can also benefit from the use of sparse prediction. Moreover, we examined two multivariable TWAS methods: TGFM [47] and TGVIS [45]. While multivariable TWAS methods also estimate causal effects, their primary focus lies in calibrating causality using PIPs.

The number of IVs was set to $m=200$ and the number of exposures to $p=10$, with local heritability of exposures at 0.3 and the local heritability of outcome at 0.001, reflecting scenarios similar to real data analysis. We set the causal effect vector as $\boldsymbol \theta = (1, 0, 0.5, \dots , 0)^\top $. To ensure the local heritability equaled 0.001, the variance of the outcome was calculated accordingly. Each exposure had 3 randomly assigned informative xQTLs, while 1 or 2 horizontal pleiotropy variants were also randomly generated. The outcome heritability explained by exposures and horizontal pleiotropy was set at a 1:1 ratio. The outcome GWAS sample size was fixed at 500000, while xQTL sample sizes for exposures were set to 300, 3000, and 30 000 which aligns with most of the real-data *cis*-MVMR studies. Further details can be found in the Supplemental Materials. The simulations were replicated 500 times.

**Figure 3:**
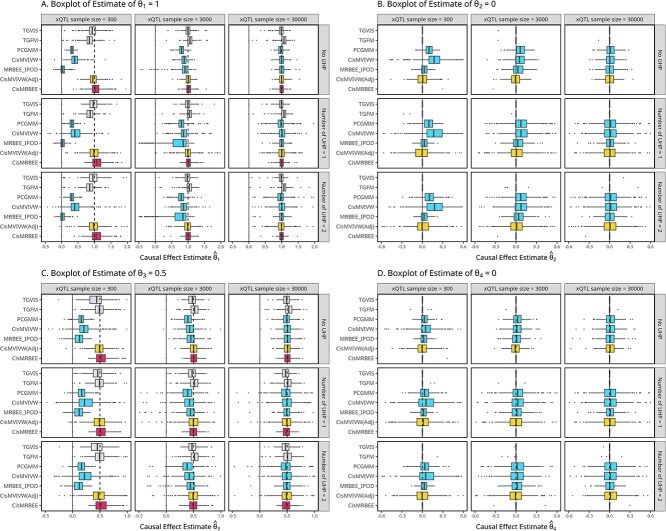
Boxplots of causal effect estimates ($\hat{\theta }_{1}$ to $\hat{\theta }_{4}$) for *cis*-MRBEE, *cis*-MVIVW (Adjusted), MRBEE-IPOD, *cis*-MVIVW, and PCGMM across different sample sizes of xQTLs. A. to D. the causal estimates when $\theta _{1} = 1$, $\theta _{2} = 0$, $\theta _{3} = 0.5$, $\theta _{4} = 0$, respectively. The figure illustrates the performance of each method in estimating causal effects under different conditions, including the presence or absence of UHP. The true causal effect vector $\boldsymbol \theta =(1, 0, 0.5, \dots , 0)^\top $, corresponding to a 0.001 local heritability of the outcome.

Figures 3A- 3D display boxplots of $\hat \theta _{1}$ to $\hat \theta _{4}$ estimated by the 7 methods across different simulated models, when the number of informative xQTL for each exposure is 3. The results showed that *cis*-MRBEE provided unbiased causal effect estimates for $n_{\text{xQTL}}=300, 3000$, or $30\,000$. When the true causal effects were zero, *cis*-MRBEE correctly excluded these exposures with zero effect. For $n_{\text{xQTL}}=300$ or $3000$, MRBEE-IPOD greatly underestimated the causal effects, and the underlying reason is that MRBEE-IPOD tended to classify outcome-associated informative variants as horizontal pleiotropy when without accounting for the underlying sparse genetic architecture. Therefore, its performance resembled a fine-mapping method of outcome GWAS and could not detect causal effects of exposures. When $n_{\text{xQTL}}=30\,000$, MRBEE-IPOD and *cis*-MRBEE performed similarly, with *cis*-MRBEE further penalizing the causal effect estimates of noncausal exposures to be exactly zero. We observed that $\hat \theta _{2}$ yielded by *cis*-MVIVW and PC-GMM were significantly biased when $n_{\text{xQTL}}=300$ and 3000. We also observed bias in $\hat \theta _{2}$ and $\hat \theta _{4}$, despite their true values being 0, as the second and fourth exposures are correlated with the first and third exposures. This bias disappeared when $n_{\text{xQTL}} = 30\,000$, implying that it arises from weak instrument bias caused by estimation error. The causal effects estimated by adjusted *cis*-MVIVW had smaller variances and suffered from reduced biases than *cis*-MVIVW, providing the evidence that *cis*-MVMR methods could benefit from the use of sparse prediction. Both TGFM and TGVIS estimates exhibited slight bias even when $n_{\text{eQTL}}=30\,000$, with TGFM showing greater bias.

**Figure 4:**
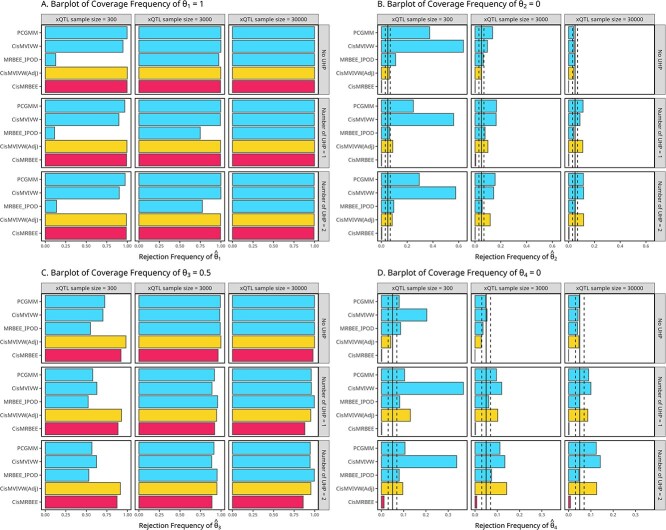
Barplots illustrating the power and type I error rates for causal effect estimates ($\hat{\theta }_{1}$ to $\hat{\theta }_{4}$) across multiple methods (*cis*-MRBEE, *cis*-MVIVW (Adjusted), MRBEE-IPOD, *cis*-MVIVW, and PCGMM) and different xQTL sample sizes. Panels A and B. The power for detecting nonzero causal effects ($\theta _{1} = 1$ and $\theta _{3} = 0.5$). Panels C and D. Type I error rates for null causal effects $\theta _{2} = 0$ and $\theta _{4} = 0$, respectively. The figure illustrates the performance of each method in power or type-I error under different conditions, including the presence or absence of UHP. The true causal effect vector $\boldsymbol \theta =(1, 0, 0.5, \dots , 0)^\top $, corresponding to a 0.001 local heritability of the outcome. In Panels B and D, the vertical solid line indicates the Type I error rate of 0.05, while the two dashed lines represent the confidence interval with a width of $\pm 2\sqrt{0.95*0.05 / 500}$.

The frequency of rejecting $H_{0}:\theta _{j}=0$, $j=1,\dots ,4$ was presented in Figs 4A- 4D. For *cis*-MVIVW, adjusted *cis*-MVIVW, PC-GMM, and MRBEE-IPOD, $H_{0}$ was rejected if the $P$-value was <0.05. For *cis*-MRBEE, $H_{0}$ was rejected if the exposure was not selected by SuSiE or the $P$-value was <0.05. Two TWAS methods used PIP > 0.5 to calibrate causality. We observed that all the methods except for MRBEE-IPOD exhibited high power across various cases (Figs 4A and 4B). We also observed that PC-GMM and two *cis*-MVIVW methods exhibited inflated type-I error in the presence of horizontal pleiotropy, even when $n_{xQTL}=30\,000$. In comparison, *cis*-MRBEE maintained high power and a well-controlled type-I error rate. In addition, both TGFM and TGVIS achieved high power and maintained well-controlled type-I errors. Note that since *cis*-MRBEE, TGFM, and TGIVS incorporate variable selection, their actual Type-I error rates can be lower than the theoretical value of 0.05 based on hypothesis testing.

We provide additional simulation results in the Supplementary Materials. First, we investigated the coverage frequency across all methods except for the two TWAS approaches (Figure S1). *Cis*-MRBEE had a leading performance generally, although its coverage frequency was$\sim $90% for $\hat{\theta }_{3}$ in the presence of horizontal pleiotropy. We also conducted parallel analyses to evaluate the unbiasedness, power, and coverage frequency when each exposure had only one informative xQTL, which were generally consistent with the results in the case of three informative xQTLs (Figures S2–S4). *Cis*-MRBEE was unbiased no matter whether the LD was low or high, but the coverage frequency was reduced in the cases of high LD (Figures S5 and S6). In addition, we examined whether the proposed discrete differential penalty (17) could mimic SuSiE’s single effect. The results demonstrated that the single effect estimated using this method was unbiased, with a coverage frequency consistently around 95% (Figure S7). Furthermore, we compared the performance of *cis*-MRBEE with MRAID [19], *cis*-MRcML [20], *cis*-IVW, and PCGMM under the *cis*-UVMR model (Figures S10 and S11). Both *cis*-MRcML and MRAID suffered from low power because they tended to act as a fine-mapping method that directly identified the informative variants of the outcome as horizontal pleiotropy. In contrast, *cis*-MRBEE did not suffer from this issue. All the results support that *cis*-MRBEE generally outperforms the existing methods in both *cis*-UVMR and *cis*-MVMR.

### Cis-MVMR analysis of lipoproteins on blood lipids

Angiopoietin-like 3 (ANGPTL3) is a liver-derived circulating factor that inhibits the enzyme lipoprotein lipase [55]. A recent *cis*-UVMR analysis [56] reported that the circulating ANGPTL3 levels have total causal effects on LDL-C, HDL-C, and TG by using three *cis*-pQTLs as IVs. However, emerging evidence suggests that some genetic associations identified by GWAS may be mediated by proteins whose coding regions are located outside the locus, possibly even on different chromosomes. For example, Sun *et al*. [49] reported that GWAS signals for COVID-19 hospitalization at the *ICAM5* and *MUC2* loci colocalized with *trans*-pQTLs for proteins encoded on other chromosomes. Motivated by this observation, we performed a *cis*-MVMR analysis to jointly evaluate ANGPTL3 and other proteins that have *trans*-pQTLs on the *ANGPTL3* locus, in order to investigate whether ANGPTL3 is directly or indirectly causal for the lipid traits.

Table 1 summarizes the details of the GWAS summary data used in this analysis. The coding regions of ANGPTL3 and PCSK9 are located on chromosome 1, those of APOA1, APOA3, and APOA5 are located on chromosome 11, and that of APOC1 is located on chromosome 19. We excluded proteins such as LPL and APOB that lack significant *trans*-pQTLs in this region and retained the six selected proteins that have at least one informative variant at this locus. All data are based on European populations. A total number of 313 variants were selected as IVs which have a minimum $P$-value of association with proteins and lipids less than P<5E-5 and $r^{2}$<0.64. We used an LD reference panel calculated from the 9,680 European individuals randomly sampled from the UK Biobank, which contains 9324 716 common variants. Supplementary Materials provide the data and codes.

**Table 1:** GWAS summary data used in real data analysis. If two sources are listed for a particular trait, this indicates that we performed a meta-analysis by combining data from the two sources. The effective sample size is reported for binary traits

Trait	Tissue	Sample Size	Source (ref #)
High-density lipoprotein cholesterol (HDL-C)		1320 016	[48]
Low-density lipoprotein cholesterol (LDL-C)		1320 016	[48]
Triglyceride (TG)		1320 016	[48]
Angiopoietin-like protein 3 (ANGPTL3)	Plasma	69 016	[49, 50]
Apolipoprotein A1 (APOA1)	Plasma	33 995	[49]
Apolipoprotein A5 (APOA5)	Plasma	35 357	[50]
Apolipoprotein C1 (APOC1)	Plasma	69 287	[49, 50]
Apolipoprotein C3 (APOC3)	Plasma	35 357	[50]
Proprotein convertase subtilisin/kexin type 9 (PCSK9)	Plasma	69 447	[49, 50]
Alzheimer’s disease (AD)		103 772	[51]
Amyloid-beta-42 (A$\beta $42)	Cerebrospinal fluid	8074	[52]
Phosphorylated tau (pTau)	Cerebrospinal fluid	7,798	[52]
Complement receptor 1 (*CR1*)	Basal Ganglia	208	[53]
Complement receptor 1 (*CR1*)	Cerebellum	457	[53]
Complement receptor 1 (*CR1*)	Cortex	2239	[53]
Complement receptor 1 (*CR1*)	Hippocampus	168	[53]
Complement receptor 1 (*CR1*)	Spinal Cord	108	[53]
Complement receptor 1 (*CR1*)	Endothelial	192	[54]
Complement receptor 1 (*CR1*)	Microglia	192	[54]
Complement receptor 1 (*CR1*)	Oligodendrocytes	192	[54]
Complement receptor 1 (*CR1*)	Pericytes	192	[54]

**Figure 5:**
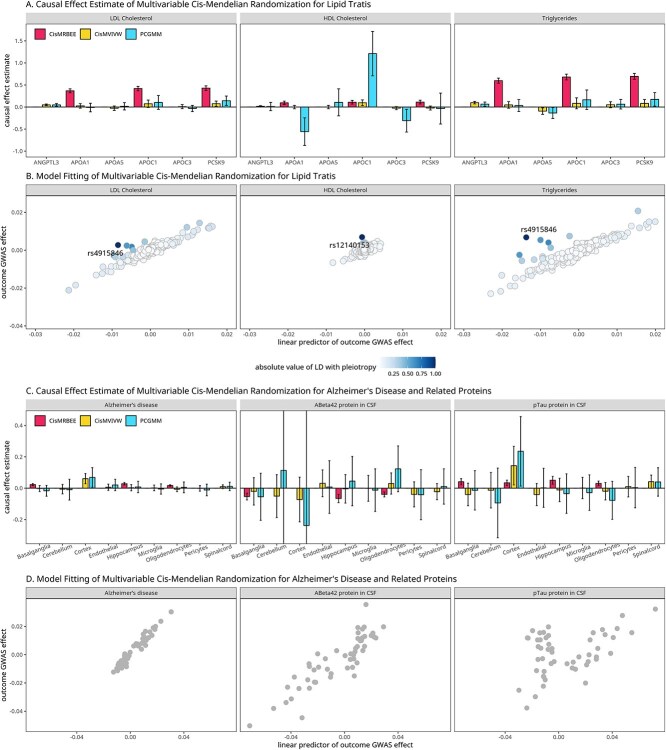
Causal effect estimations in real data analysis. A. Causal effect estimates and associated double SEs error bars of proteins such as ANGPTL3 on LDL-C, HDL-C, and TG, using pQTLs at the *ANGPTL3* locus. B. The scatter plot of the outcome GWAS effects versus their linear predictor, i.e. $\hat{\mathbf B}^{\text{PLS}}\hat{\boldsymbol \theta }_{\text{cis-MRBEE}}$. We highlighted the horizontal pleiotropy and used a color gradient to indicate the LD between other IVs and this horizontal pleiotropy. C. Causal effect estimates and associated double SEs of gene expression of the *CR1* gene in tissues including the cortex and cells like pericytes within cortex tissue on AD and PD, using eQTLs and sceQTLs at the *CR1* locus. D. The scatter plot of the outcome GWAS effects versus their linear predictor. Confidence intervals were calculated while accounting for Bonferroni correction.

Figure 5A shows that *cis*-MRBEE identified a credible set that uniformly contained APOA1, APOC1, and PCSK9 as causal for the three traits. In contrast, *cis*-MVIVW and PCGMM identified multiple proteins including ANGPTL3, APOA1, and PCSK9 as causal for LDL-C and TG but lacked credible set incorporation, making their results harder to interpret. These three proteins were identified by SuSiE within the same credible set, which suggests that at least one of them is causal for the outcomes. However, the current data do not allow us to further distinguish whether all or only some of them are truly causal. Figure 5B illustrates that *cis*-MRBEE also identified rs4915846 as horizontal pleiotropy for LDL-C and TG, and rs12140153 for HDL-C. After accounting for these two pleiotropy and the variants in LD with them, APOA1, APOC1, and PCSK9 could accurately explain the local variations of LDL-C, HDL-C, and TG. This result suggests that ANGPLT3 may not have direct causal effects for lipid traits.

To further investigate if ANGPTL3 is indirectly causal for lipids traits, we conducted subsequent analyses. First, a *cis*-UVMR analysis using *cis*-MRBEE identified a significant total causal effect of ANGPTL3 on lipid traits (Figure S14). To further explore these relationships, we performed a bi-directional *cis*-UVMR analysis, which revealed that ANGPTL3 was causally associated with APOA1, APOC1, and PCSK9, whereas these three proteins did not exhibit a causal effect on ANGPTL3 (Figures S16 and S17). This suggests that ANGPTL3 may contribute to the local genetic variations of lipid traits through the mediation of these three proteins, as the direct causal effect of ANGPLT3 was not detected in our *cis*-MVMR analysis. Additionally, we conducted traditional UVMR analyses using IVs across the genome, which indicated that ANGPTL3 contributed only to TG levels (Figure S18). However, MVMR analyses using IVs across the genome did not support a direct effect of ANGPTL3 on TG, LDL-C, or HDL-C (Figure S19). Furthermore, a bi-directional MR analysis of protein-protein co-regulation networks revealed reciprocal causality between ANGPTL3 and APOC1, with ANGPTL3 also regulating APOC3 (Figure S25). Taken together, our findings suggest that ANGPTL3 regulate lipid levels through mediating APOA1, APOC1 and PCSK9.

### Cis-MVMR analysis of tissue-specific expressions of CR1 on Alzheimer’s disease

AD is a progressive neurological disorder characterized by the gradual degeneration of brain cells [51]. A$\beta $42 and pTau are two major proteins implicated in the AD pathological process. Recent GWAS have identified strong associations for AD and A$\beta $42 at the complement receptor 1 (*CR1*) locus [51, 52]. Some biological studies suggest that *CR1* may be linked to AD through immune pathways, although evidence regarding the risk or the protective role of *CR1* expression on AD remains inconsistent [57]. Furthermore, *CR1* overexpression is observed to contribute to neuroinflammation and neuronal damage, and its expression levels are positively correlated with neurofibrillary tangle density and pTau abundance [55].

We conducted a *cis*-MVMR analysis at the *CR1* locus, using tissue- and cell-type-specific expressions of the *CR1* gene to: (1) evaluate the potential risk or protective role of *CR1* in AD, A$\beta $42 and pTau in CSF, and (2) identify the causal tissues involved. Table 1 summarizes the data used in this data analysis. Other genes with potentially *trans*-eQTL or *trans*-sceQTL located at this locus were not considered due to the unavailability of data. All data are based on European populations. A total number of 51 variants were selected as IVs using the same LD reference panel and the same criteria as the previous analysis. Note that A$\beta $42 levels in CSF are inversely correlated with total A$\beta $ load in the brain [58, 59], while pTau levels in CSF are significantly positively correlated with pTau concentration in the brain [60]. Thus, in this *cis*-MVMR analysis, their CSF levels served as inverse and direct proxies, respectively, for their brain loads.

The causal effects estimated by the three methods are shown in Fig. 5C. *Cis*-MRBEE identified the basal ganglia, hippocampus, and oligodendrocytes as causal tissues and cell-type, with *CR1* increasing the risk on AD and pTau while decreasing A$\beta $42. In particular, the *CR1*-basal-ganglia, *CR1*-hippocampus, and *CR1*-oligodendrocytes pairs were in the same credible set for all AD, A$\beta $42 and pTau, suggesting that the link between AD, A$\beta $42, and pTau might be mediated through *CR1* expression in both basal ganglia and hippocampus tissues, as well as oligodendrocytes. Figure 5D shows that no underlying horizontal pleiotropy was detected for these three traits. Results from *cis*-MVIVW and PC-GMM in this region also differed from *cis*-MRBEE. Both *cis*-MVIVW and PC-GMM identified only the cortex as a causal tissue, possibly due to the larger sample size of the cortex than the basal ganglia, hippocampus, and oligodendrocytes. When the outcome was A$\beta $42 or pTau, neither method detected causality after multiple testing corrections. This highlights that when the sample sizes of exposures xQTL studies are small, sparse prediction can remove substantial estimation errors in xQTL effect sizes and yield a better causal estimation in *cis*-MVMR.

## Discussion

In this paper, we propose a new method called *cis*-MRBEE method, which, to our knowledge, is the first *cis*-MVMR method to address weak instrument bias driven by estimation errors of genetic effects while simultaneously selecting causal exposures and horizontal pleiotropy. Unlike traditional MVMR methods, *cis*-MRBEE leverages the sparse genetic architecture in *cis* regions and introduces a sparse prediction approach to effectively reduce estimation errors. In its implementation, *cis*-MRBEE applies a double-regularized minimization where SuSiE is used to select causal exposures and MCP is employed for controlling horizontal pleiotropic effects. In addition, we introduced a discrete differential penalty, offering an effective method to incorporate credible sets identified by SuSiE into non-Bayesian *cis*-MVMR approaches. This represents an alternative innovation compared with existing pipelines [61]. Our simulations showed that directly adapting standard MRBEE to *cis*-MVMR was infeasible, and existing *cis*-MVMR approaches failed to control type I errors in the presence of horizontal pleiotropy. By using the adjusted *cis*-MVIVW as an example, we further demonstrated that the existing *cis*-MVMR approaches could also benefit from the use of sparse prediction.

Recently, Patel *et al*. [28] introduced a concept of phenotypic heterogeneity, referring to the local genetic correlations among exposures (phenotypic heterogeneity) and between an exposure and outcome (overdispersion heterogeneity). This concept is contradictory with neither our *cis*-MVMR model (1-2 and 7-8) nor the local sparse genetic architecture assumption. Indeed, *cis*-MRBEE further allows a lack of phenotypic heterogeneity among partial exposures, solving this issue by grouping into the same credible set using SuSiE. The interpretation is that at least one exposure in the credible set is likely causal to the outcome. *Cis*-MRBEE also accounts for overdispersion heterogeneity when estimating the SE of the causal effect estimates (Supplementary Materials), although our *cis*-MVMR model (1-2 and 7-8) does not assume its presence. The superior performance of *cis*-MRBEE in simulations and real data likely arises from the use of sparse prediction to minimize estimation errors, as well as its capability to select causal exposures correctly and control horizontal pleiotropic effects. On the other hand, *cis*-MR and TWAS share some methodological similarities and both aim to identify causal phenotypes that can explain genetic variations in a locus affecting the outcome. However, current TWAS approaches, including TGFM [47] and TGVIS [45], do not particularly emphasize the unbiasedness of causal effect estimation. In simulations, we did observe that these methods suffered from slight bias. In the future, the unbiased estimating equations from *cis*-MRBEE could be leveraged to improve existing TWAS methods.

Our real data analysis provided key insights. Specifically, our *cis*-MVMR analysis on the *ANGPTL3* locus provides a novel interpretation: the impact of ANGPTL3 on lipids is mediated through APOC1, APOC1, and PCSK9. In our *cis*-MVMR analysis including ANGPTL3, APOC3, APOC1, APOC1, and PCSK9 as exposures, we found both ANGPTL3 and APOC3 no longer affected these lipids. We followed with a bi-directional *cis*-UVMR analysis among the six proteins, and observed that ANGPTL3 showed significant effects on APOA1, APOC1, and PCSK9 but not in opposite directions. This suggests that the effects of ANGPTL3 on lipid traits are mediated through APOC1, APOC1, PCSK9, or other proteins, as also discussed in [55]. It should be pointed out that the conclusion that ANGPTL3 is not a direct causal protein for LDL-C or TG does not conflict with existing drug trials [62]. First, our *cis*-UVMR analysis showed that AGNPTL3 had a total causal effect on lipids, which means that ANGPTL3 can affect lipid levels via the mediation/interaction with these proteins. Second, the pQTL summary data are derived from blood tissue rather than liver tissue, which may lead to false negatives in detecting direct causal effects. In the future, when non-blood pQTL data become available [63], corresponding analyses should be conducted to verify the tissue heterogeneity of pQTLs.

Second, we identified that *CR1* gene expression in the basal ganglia, hippocampus, and oligodenocytes are likely causal for AD, A$\beta $42, and pTau levels in CSF, with positive and negative directions of causal effects, respectively. In addition, this finding suggests that *CR1* might increase brain A$\beta $ and pTau loads, thereby elevating AD risk. This inference aligns with the latest clinical trial: lecanemab reduces brain amyloid levels and is associated with moderately less decline in cognitive and functional measures [64]. Moreover, a recent experiment revealed that oligodendrocytes produce A$\beta $ and contribute to plaque formation alongside neurons in AD model mice [65]. Further research is warranted to investigate whether *CR1* plays a key role in this process, particularly given that *CR1* is one of only two genome-wide significant loci of A$\beta $42 [52].

Some limitations for *cis*-MRBEE warrant to discuss. First, *cis*-MRBEE is a two-stage approach: selecting informative variants in the first stage and estimating causal effects in the second stage. This two-stage approach leads to uncertainty from the selection stage that is not propagated into the estimation stage, which may explain why *cis*-MRBEE exhibited coverage frequencies lower than 0.95 when the sample size of the exposure was small. In the future study, the method simultaneously selecting informative variants and estimating causal effects, such as the one used in MRAID [19], can be adopted to improve *cis*-MRBEE. Second, *cis*-MRBEE assumes a sparse genetic architecture, while genomic regions with polygenic architectures (e.g. regulatory hubs) may violate this assumption [66]. Our additional simulations showed that *cis*-MRBEE is still robust to a moderate degree of polygenicity. Third, *cis*-MRBEE recommends using PLINK to remove highly correlated variants before selecting informative variants, which could inadvertently exclude true causal variants. According to [33], variable selection typically serves two purposes: building an accurate predictor and gaining insights into underlying biological mechanisms. At the informative variant selection stage, the goal is not necessarily to select the true causal variants, but rather to identify variants that effectively predict the exposures. Therefore, we recommend using moderate clumping when variants are in high LD in the *cis*-MVMR analysis. Third, the computational cost may be high if the number of IVs is large. Our supplementary analysis showed that, reassuringly, *cis*-MRBEE maintained efficiency comparable with PCGMM when we reduced the number of IVs to a few hundred using C+T. In particular, this clumping procedure had a minimal impact on causal effect estimation, and conversely, it improved computational efficiency. In Supplementary Materials, we discuss these issues in more detail and examine the robustness of *cis*-MRBEE with simulations and real data analysis.

**Key Points**

- Summarily, our developed *cis*-MRBEE and software represent a major advancement in *cis*-MVMR, with applications in drug discovery and repurposing.
- Conceptually, we propose a sparse genetic architecture: informative variants in a genomic region are sparse.
- Methodologically, *cis*-MRBEE is a two-stage method: it first identifies informative variants via fine-mapping and re-estimating genetic associations, and then applies a penalized minimization to estimate causal exposures and account for pleiotropic effects.
- Using *cis*-MRBEE, we showed that plasma ANGPTL3 levels may indirectly regulate lipid levels via the protein co-regulation network.
- With *cis*-MRBEE, we discovered *CR1* expression in the basal ganglia, hippocampus, and oligodendrocytes as likely causal for AD, A$\beta $42, and pTau levels in CSF, consistent with recent findings.

## Supplementary Material

Supplementary_Materials_bbaf250

## Data Availability

The GWAS summary data, eQTL summary data, and pQTL summary data used in this study can be downloaded from the “Data available” section of the related literature.

## Code availability

The code for the analyses presented in this paper is available in the Supplemental Materials, complete with step-by-step instructions. MRBEEX package is available at https://github.com/harryyiheyang/MRBEEX.
