# Supplementary material for: A multivariable cis-Mendelian randomization method robust to weak instrument bias and horizontal pleiotropy bias
Source: Brief Bioinform. 2025 Jun 5;26(3):bbaf250. doi: 10.1093/bib/bbaf250 (PMC12140020; doi:10.1093/bib/bbaf250)
Supplement: Supplementary_Materials_bbaf250 [file supplementary_materials_bbaf250.pdf]

Supplementary Materials of A multivariable cis-Mendelian randomization method robust to weak instrument bias and horizontal pleiotropy bias

Yihe Yang, Noah Lorincz-Comi, Mengxuan Li, Xiaofeng Zhu

Contents

1 Real data analysis **2**
1.1 Data structure 2
1.2 Step-by-step analysis of ANGPLT3 3
1.3 Step-by-step analysis of CR1 10

2 Simulation **15**
2.1 Examination of discrete differential penalty 18
2.2 Simulations of Maximum correlation of LD 20
2.3 Simulations of univariable cis-Mendelian randomization 25

3 Supplementary Statistical Method **28**
3.1 Multivariable cis-Mendelian randomization model 28
3.2 Sparse prediction of GWAS effect 30
3.3 Estimation of causal effect and horizontal pleiotropy 32
3.4 Deduction of expectation of estimating equation 34
3.5 Dealing with credible sets using discrete differential penalty 36
3.6 Covariance matrix of causal effect estimate 36
3.7 Strength of horizontal pleiotropy 37
3.8 Implementation 38
3.9 Algorithm 38

4 Supplementary Issues During Peer Review **38**
4.1 Polygencity of human complex trait 39
4.2 Computing Time 41
4.3 Influence of clumping threshold r^2 42
4.4 Lack of causal variants 44
4.5 Irrepresentable condition 45
4.6 Influence of local heritability 46

5 Settings in Real Data Analysis **49**
5.1 C+T in cis-MR analysis 49
5.2 C+T in MVMR analysis using genome-wide significant IVs 49
5.3 C+T in UVMR analysis using genome-wide significant IVs 49
5.4 Meta-analysis of pQTL summary data 49

6 Supplementary Figures **51**

1 Real data analysis

1.1 Data structure

Here we illustrate a real example of how to perform cis-MRBEE in real data analysis of the manuscript. This tutorial starts with the structures of involved data.

1.1.1 LD reference panel

The first dataset is the LD reference panel. This dataset is derived from the 9,680 unrelated individuals we described in the paper, selected from approximately 500,000 imputed individuals in the UK Biobank (UKBB). We refined the data using the bim files from these individuals. The files we shared on Google Drive include all 9.32 million SNPs involved; however, in this tutorial, we will focus only on a subset in the ANGPTL3 or CR1 locus. Below is a glimpse of the data structure:

```
library(data.table)
library(dplyr)
variant=readRDS("RDS/ANGPTL3_variant.rds")%>%as.data.frame(.)
head(variant)
```

```
##                SNP CHR       BP A1 A2      Freq MarkerName
## 1         rs334732   1 61600399  T  C 0.0617230 1:61600399
## 2         rs334731   1 61602342  A  G 0.0441490 1:61602342
## 3 1:61602418_GT_G   1 61602418  G GT 0.9105647 1:61602418
## 4         rs334730   1 61602706  T  C 0.0442680 1:61602706
## 5        rs4915729   1 61602963  G  A 0.9104124 1:61602963
## 6        rs4915730   1 61603158  C  G 0.9104567 1:61603158
```

In this reference panel, `SNP` is the identifier for the variants; `CHR` represents the chromosome; `BP` indicates the base pair position in the hg19 genome build; `A1` is the effect allele as specified in the BED file; `A2` is the other allele; `Freq` denotes the frequency of the effect allele; and `MarkerName` is another unique identifier for the variants in the format CHR:BP. It is important to note that some variants in the UKBB bed file do not have an rsID. For these variants, their `SNP` are in the format CHR:BP:A2:A1.

1.1.2 GWAS and xQTL summary data

The second dataset is the GWAS and xQTL summary data, which should include at least the following columns: `SNP`, `A1`, `A2`, `Zscore`, and `N`. In this dataset, `Zscore` represents the Z-score of the marginal effect size estimates from the outcome GWAS, while `N` denotes the sample size. Other statistics can be deduced from `Zscore` and `N`, e.g.,

$$\texttt{BETA} = \frac{\texttt{Zscore}}{\sqrt{\texttt{N}}}, \quad \texttt{SE} = \frac{1}{\sqrt{\texttt{N}}}.$$

Below is an example of the dataset's structure:

```
datalist=readRDS("RDS/ANGPTL3_datalist.rds")
list2env(datalist,envir=.GlobalEnv)
```

```
## <environment: R_GlobalEnv>
```

```
head(LDL)
```

```
##                 SNP CHR       BP A1  A2     Zscore       N
## 1  1:62010290_CT_C   1 62010290  C  CT  1.3774111 1071241
## 2  1:62020511_AT_A   1 62020511 AT   A -0.5402734  390326
## 3  1:62030258_CA_C   1 62030258 CA   C -0.4779975 1082893
## 4 1:62076847_CAG_C   1 62076847  C CAG  0.2391612 1094352
## 5 1:62079302_GAC_G   1 62079302  G GAC -0.7518729 1091695
```

```
## 6  1:62085587_TG_T   1 62085587 TG   T -1.3232658 1072869
```

```r
head(ANGPTL3)
```

```
##                 SNP CHR       BP A1  A2     Zscore     N
## 1  1:62010290_CT_C   1 62010290  C  CT -0.2841204 69019
## 2  1:62020511_AT_A   1 62020511 AT   A  0.3600681 69019
## 3  1:62030258_CA_C   1 62030258 CA   C -1.9646074 69019
## 4 1:62076847_CAG_C   1 62076847  C CAG -2.7906215 69020
## 5 1:62079302_GAC_G   1 62079302  G GAC  2.7658935 69020
## 6  1:62085587_TG_T   1 62085587 TG   T  1.4495517 69019
```

It should be noted that we did not use the original `SNP` identifiers from the GWAS. Instead, we merged the GWAS data with the variant file using the `MarkerName` (CHR:BP) identifiers, and then assigned `SNP` from the variant file to the corresponding entries in the GWAS data. In cases where the GWAS file is based on the hg38 genome build, we use LiftOver to convert it to hg19.

1.1.3 LD refernece panel with individual

We used the UKBB BED file to estimate the LD reference, with a sample size of 9,680. Below is a glimpse of the data structure:

```r
UKBBGenotype=readRDS("RDS/ANGPTL3_LDref.rds")
UKBBGenotype[1:10,1:5]
```

```
##                 rs4915763 rs6699079 rs12036653 rs6694989 rs6686620
## 1000559-1000559         2         2          1         1         2
## 1000916-1000916         2         1          2         1         1
## 1001097-1001097         1         1          2         1         1
## 1001150-1001150         2         1          1         1         2
## 1001312-1001312         2         1          2         2         2
## 1002233-1002233         2         2          2         2         2
## 1003235-1003235         2         2          2         2         2
## 1004066-1004066         2         2          1         1         2
## 1004469-1004469         2         2          2         2         1
## 1004972-1004972         1         2          2         1         1
```

Note that the data may contains very few missing values. Such missing values are imputed by the means of non-missing values column-by-column.

1.2 Step-by-step analysis of ANGPLT3

In the first step, we adjust the direction of the Z-scores in the GWAS and xQTL summary data to ensure that the effect alleles in these datasets match the effect alleles in our reference panel. This step is crucial because the LD matrix is estimated from this reference panel, and accurate LD estimation is fundamental to all statistical methods based on GWAS summary data. We wrote a function, `allele_harmonise()` in the R package `MRBEEX`, to perform this step:

```r
library(MRBEEX)
datalist=filter_align(list(LDL=LDL,HDL=HDL,TG=TG,ANGPTL3=ANGPTL3,APOA1=APOA1,
APOC1=APOC1,APOA5=APOA5,APOC3=APOC3,PCSK9=PCSK9),
ref_panel=variant[,c("SNP","A1","A2")],allele_match=T)
```

```
## [1] "Adjusting effect allele according to reference panel..."
## [1] "Finding common SNPs..."
## [1] "Aligning data to common SNPs and ordering..."
## [1] "Filtering complete."
```

```
ZMatrix=matrix(0,dim(datalist[[1]])[1],length(datalist))
NMatrix=matrix(0,dim(datalist[[1]])[1],length(datalist))
for(i in 1:length(datalist)){
ZMatrix[,i]=datalist[[i]]$Zscore
NMatrix[,i]=datalist[[i]]$N
}
rownames(ZMatrix)=rownames(NMatrix)=datalist[[1]]$SNP
colnames(ZMatrix)=colnames(NMatrix)=names(datalist)
```

In `allele_harmonise()`, we automatically set `gwas_data` as a data.table with `key="SNP"`, allowing `eQTLsQTL=eQTLsQTL[LDL, nomatch=0]` to efficiently merge the two datasets. The reason for merging these datasets, as described in here, there is a vast and heterogeneous landscape of publicly available GWAS. These studies were conducted on different genotyping platforms, using different imputation schemes, and defined on different releases of the human genome, is that there is a vast and heterogeneous landscape of publicly available GWAS. These studies were conducted on different genotyping platforms, using different imputation schemes, and defined on different releases of the human genome. Therefore, we aim to find the common variants between the GWAS and xQTL summary data to perform the analysis.

1.2.1 Extracting the moderately correlated variants

The next step is to remove highly correlated variants using C+T. Although SuSiE can group highly correlated or statistically duplicated variants into a single group and assign them one effect, including many redundant variants can significantly increase the dimensionality of the model. Therefore, primarily to enhance computational efficiency, we recommend retaining only moderately correlated variants.

We use the smallest p-value across all exposures corresponding to each variant as the input p-value for PLINK to extract a subset of moderately correlated variants. While we will not execute the following steps in this tutorial, we will provide the code for you. You can modify the file paths as needed for your own data.

```
MinP=apply(ZMatrix^2,1,max)%>%pchisq(.,1,lower.tail=F)
jointtest=data.frame(SNP=rownames(ZMatrix),P=MinP)
write.table(jointtest,"ANGPTL3_Protein.txt",row.names=F,quote=F,sep="\t")
system("./plink --bfile your_bed_file --clump ANGPTL3_Protein.txt --clump-field P
--clump-kb 1000 --clump-p1 5e-5 --clump-p2 5e-5 --clump-r2 0.64
--out ANGPTL3_Protein")
IVlist=fread("ANGPTL3_Protein.clumped")%>%dplyr::select(SNP,CHR,BP,P)
```

The most important part in this step is:

- `-clump-kb 1000`: we consider the window size to be 1M,
- `-clump-p1 5e-5`: we use the threshold of 5E-5,
- `-clump-r2 0.5`: the correlation between two variants is in the range $(-0.8, 0.8)$.

We have recorded this pool of variants in the PCSK9 locus:

```
IVlist=readRDS("RDS/ANGPTL3_IVlist.rds")
```

1.2.2 Regularization of LD matrix

Our next step is to estimate a "good" LD matrix. Note that when the dimension of the LD matrix is large (e.g., $m > 100$), we suggest using the POET method (Fan et al., 2013), as described in this paper, to regularize such an LD matrix. The code is as

```
R=cor(UKBBGenotype)
R[is.na(R)]=0;diag(R)=1
R=TGVIS::poet_shrinkage(R)
R=(t(R)+R)/2
```

```r
genosnp=colnames(UKBBGenotype)
rownames(R)=colnames(R)=genosnp
```

1.2.3 Performing cis-MVMR analysis

First, we organize the data, ensuring that the order of rows in the effect size matrix, SE matrix, and LD matrix must match precisely. It is worth noting that we use genome-wide exposures and outcome summarized statistics to estimate the correlation matrix of estimation errors. For specific examples, please refer to https://github.com/noahlorinczcomi/MRBEE and subsequently the analysis of CR1.

```r
ZY=ZMatrix[genosnp,1:3]
ZX=ZMatrix[genosnp,-c(1:3)]
NY=NMatrix[genosnp,1:3]
NX=NMatrix[genosnp,-c(1:3)]
Rxy=readRDS("RDS/Rxy.rds")
```

First, we analyze LDL-C:

```r
by=ZY[,"LDL"]/sqrt(NY[,"LDL"])
byse=1/sqrt(NY[,"LDL"])
bX=ZX/sqrt(NX)
bXse=1/sqrt(NX)
NAM=c(colnames(bX),"LDL")
```

Next, we perform cis-MVMR analysis using cis-MRBEE, cis-MVIVW, and PCGMM:

```r
library(MendelianRandomization)
MVINPUT=mr_mvinput(bx=bX,by=by,bxse=bXse,byse=byse,correlation=R)
fitMRBEE=MRBEEX::CisMRBEEX(causal.pip.thres=0.2,by,bX,byse,bXse,LD=R,Rxy=Rxy[NAM,NAM],
                          reliability.thres=0.75,xQTL.max.L=15,
                          xQTL.pip.thres=0.3,xQTL.Nvec=colMeans(NX),
                          tauvec=seq(4.5,30,1.5),susie.iter=500,
                          ridge.diff=100,ebic.gamma=0)
```

```
## Please standardize data such that BETA = Zscore/sqrt n and SE = 1/sqrt n
## Sparse prediction ends: 2.754 secs
## Causal effect estimation ends: 1.554 secs
```

```r
fitCisIVW=mr_mvivw(MVINPUT,correl=T)
fitPCGMM=mr_mvpcgmm(MVINPUT,nx=colMeans(NX),ny=mean(NY[,"LDL"]),thres=0.999)
ANGPTL3_LDL=list(fitMRBEE=fitMRBEE,fitCisIVW=fitCisIVW,fitPCGMM=fitPCGMM)
```

Finally, we summarize the results. It should be noted that when using BH and BY for adjustments, we set the p-values of variables not selected by SuSiE to 1. This approach yields the most conservative results.

```r
LDL=data.frame(
Estimate=c(ANGPTL3_LDL$fitMRBEE$theta,ANGPTL3_LDL$fitCisIVW@Estimate,ANGPTL3_LDL$fitPCGMM@Estimate),
SE=c(ANGPTL3_LDL$fitMRBEE$theta.se,ANGPTL3_LDL$fitCisIVW@StdError,ANGPTL3_LDL$fitPCGMM@StdError),
Exposure=colnames(ZX),
Method=c(rep("CisMRBEE",6),rep("CisMVIVW",6),rep("PCGMM",6)))
LDL$P=pchisq(LDL$Estimate^2/LDL$SE^2,1,lower.tail=F);LDL$P[is.na(LDL$P)]=1
LDL$Outcome="LDL-C"
LDL=dplyr::select(LDL,Outcome,Exposure,Method,Estimate,SE,P)
print(LDL)
```

```
##    Outcome Exposure   Method     Estimate           SE            P
## 1    LDL-C  ANGPTL3 CisMRBEE  0.000000000 0.000000000 1.000000e+00
```

```
## 2    LDL-C    APOA1 CisMRBEE  0.334009716 0.016611870 6.448596e-90
## 3    LDL-C    APOC1 CisMRBEE  0.379902092 0.018894677 6.499206e-90
## 4    LDL-C    APOA5 CisMRBEE  0.000000000 0.000000000 1.000000e+00
## 5    LDL-C    APOC3 CisMRBEE  0.000000000 0.000000000 1.000000e+00
## 6    LDL-C    PCSK9 CisMRBEE  0.388433097 0.019319181 6.527649e-90
## 7    LDL-C  ANGPTL3 CisMVIVW  0.048920705 0.007431774 4.621631e-11
## 8    LDL-C    APOA1 CisMVIVW  0.025478782 0.019373117 1.884556e-01
## 9    LDL-C    APOC1 CisMVIVW  0.074030585 0.032068384 2.097009e-02
## 10   LDL-C    APOA5 CisMVIVW -0.027062973 0.019160721 1.578265e-01
## 11   LDL-C    APOC3 CisMVIVW  0.009620662 0.017503576 5.825665e-01
## 12   LDL-C    PCSK9 CisMVIVW  0.075633356 0.022526899 7.865968e-04
## 13   LDL-C  ANGPTL3    PCGMM  0.018697820 0.012144358 1.236505e-01
## 14   LDL-C    APOA1    PCGMM  0.044886348 0.035932152 2.115929e-01
## 15   LDL-C    APOC1    PCGMM  0.132132926 0.058640206 2.424137e-02
## 16   LDL-C    APOA5    PCGMM -0.061735551 0.033504816 6.538928e-02
## 17   LDL-C    APOC3    PCGMM  0.029837444 0.028822053 3.005617e-01
## 18   LDL-C    PCSK9    PCGMM  0.138514352 0.040765501 6.792194e-04
```

The parallel analyses for HDL-C and TG are are as follows:

```
by=ZY[,"HDL"]/sqrt(NY[,"HDL"])
byse=1/sqrt(NY[,"HDL"])
bX=ZX/sqrt(NX)
bXse=1/sqrt(NX)
NAM=c(colnames(bX),"HDL")
MVINPUT=mr_mvinput(bx=bX,by=by,bxse=bXse,byse=byse,correlation=R)
fitMRBEE=MRBEEX::CisMRBEEX(causal.pip.thres=0.2,by,bX,byse,bXse,LD=R,Rxy=Rxy[NAM,NAM],
                           reliability.thres=0.75,xQTL.max.L=15,
                           xQTL.pip.thres=0.3,xQTL.Nvec=colMeans(NX),
                           tauvec=seq(4.5,30,1.5),susie.iter=500,
                           ridge.diff=100,ebic.gamma=0)
```

```
## Please standardize data such that BETA = Zscore/sqrt n and SE = 1/sqrt n
## Sparse prediction ends: 2.193 secs
## Causal effect estimation ends: 3.011 secs
```

```
fitCisIVW=mr_mvivw(MVINPUT,correl=T)
fitPCGMM=mr_mvpcgmm(MVINPUT,nx=colMeans(NX),ny=mean(NY[,"HDL"]),thres=0.999)
ANGPTL3_HDL=list(fitMRBEE=fitMRBEE,fitCisIVW=fitCisIVW,fitPCGMM=fitPCGMM)
HDL=data.frame(
Estimate=c(ANGPTL3_HDL$fitMRBEE$theta,ANGPTL3_HDL$fitCisIVW@Estimate,ANGPTL3_HDL$fitPCGMM@Estimate),
SE=c(ANGPTL3_HDL$fitMRBEE$theta.se,ANGPTL3_HDL$fitCisIVW@StdError,ANGPTL3_HDL$fitPCGMM@StdError),
Exposure=colnames(ZX),
Method=c(rep("CisMRBEE",6),rep("CisMVIVW",6),rep("PCGMM",6)))
HDL$P=pchisq(HDL$Estimate^2/HDL$SE^2,1,lower.tail=F);HDL$P[is.na(HDL$P)]=1
HDL$Outcome="HDL-C"
HDL=dplyr::select(HDL,Outcome,Exposure,Method,Estimate,SE,P)
print(HDL)
```

```
##    Outcome Exposure   Method     Estimate          SE            P
## 1    HDL-C  ANGPTL3 CisMRBEE  0.0000000000 0.000000000 1.000000e+00
## 2    HDL-C    APOA1 CisMRBEE  0.0883027507 0.014102926 3.817640e-10
## 3    HDL-C    APOC1 CisMRBEE  0.1004548355 0.016040859 3.790121e-10
## 4    HDL-C    APOA5 CisMRBEE  0.0000000000 0.000000000 1.000000e+00
## 5    HDL-C    APOC3 CisMRBEE  0.0000000000 0.000000000 1.000000e+00
## 6    HDL-C    PCSK9 CisMRBEE  0.1027545187 0.016401222 3.726991e-10
```

```
## 7    HDL-C  ANGPTL3 CisMVIVW  0.0168719040 0.005655826 2.853434e-03
## 8    HDL-C    APOA1 CisMVIVW -0.0001636759 0.014780375 9.911645e-01
## 9    HDL-C    APOC1 CisMVIVW  0.0970515268 0.024452813 7.219578e-05
## 10   HDL-C    APOA5 CisMVIVW  0.0003542207 0.014631370 9.806854e-01
## 11   HDL-C    APOC3 CisMVIVW -0.0249734536 0.013362636 6.163649e-02
## 12   HDL-C    PCSK9 CisMVIVW -0.0225183737 0.017188313 1.901627e-01
## 13   HDL-C  ANGPTL3    PCGMM  0.0432832491 0.012507948 5.392542e-04
## 14   HDL-C    APOA1    PCGMM  0.0376825895 0.041690667 3.660688e-01
## 15   HDL-C    APOC1    PCGMM  0.2357960189 0.067000401 4.326504e-04
## 16   HDL-C    APOA5    PCGMM -0.1151982458 0.040696198 4.644798e-03
## 17   HDL-C    APOC3    PCGMM  0.0468014849 0.034303293 1.724594e-01
## 18   HDL-C    PCSK9    PCGMM -0.3800995655 0.046671606 3.820225e-16
```

```
################################################################################
by=ZY[,"TG"]/sqrt(NY[,"TG"])
byse=1/sqrt(NY[,"TG"])
bX=ZX/sqrt(NX)
bXse=1/sqrt(NX)
NAM=c(colnames(bX),"TG")
MVINPUT=mr_mvinput(bx=bX,by=by,bxse=bXse,byse=byse,correlation=R)
fitMRBEE=MRBEEX::CisMRBEEX(causal.pip.thres=0.2,by,bX,byse,bXse,LD=R,Rxy=Rxy[NAM,NAM],
                           reliability.thres=0.75,xQTL.max.L=15,
                           xQTL.pip.thres=0.3,xQTL.Nvec=colMeans(NX),
                           tauvec=seq(4.5,30,1.5),susie.iter=500,
                           ridge.diff=100,ebic.gamma=0)
```

```
## Please standardize data such that BETA = Zscore/sqrt n and SE = 1/sqrt n
## Sparse prediction ends: 2.289 secs
## Causal effect estimation ends: 4.592 secs
```

```
fitCisIVW=mr_mvivw(MVINPUT,correl=T)
fitPCGMM=mr_mvpcgmm(MVINPUT,nx=colMeans(NX),ny=mean(NY[,"TG"]),thres=0.999)
ANGPTL3_TG=list(fitMRBEE=fitMRBEE,fitCisIVW=fitCisIVW,fitPCGMM=fitPCGMM)
TG=data.frame(
Estimate=c(ANGPTL3_TG$fitMRBEE$theta,ANGPTL3_TG$fitCisIVW@Estimate,ANGPTL3_TG$fitPCGMM@Estimate),
SE=c(ANGPTL3_TG$fitMRBEE$theta.se,ANGPTL3_TG$fitCisIVW@StdError,ANGPTL3_TG$fitPCGMM@StdError),
Exposure=colnames(ZX),
Method=c(rep("CisMRBEE",6),rep("CisMVIVW",6),rep("PCGMM",6)))
TG$P=pchisq(TG$Estimate^2/TG$SE^2,1,lower.tail=F);TG$P[is.na(TG$P)]=1
TG$Outcome="TG"
TG=dplyr::select(TG,Outcome,Exposure,Method,Estimate,SE,P)
print(TG)
```

```
##    Outcome Exposure   Method    Estimate         SE             P
## 1       TG  ANGPTL3 CisMRBEE  0.00000000 0.00000000  1.000000e+00
## 2       TG    APOA1 CisMRBEE  0.59515762 0.02168543 7.960402e-166
## 3       TG    APOC1 CisMRBEE  0.67693005 0.02466547 8.093741e-166
## 4       TG    APOA5 CisMRBEE  0.00000000 0.00000000  1.000000e+00
## 5       TG    APOC3 CisMRBEE  0.00000000 0.00000000  1.000000e+00
## 6       TG    PCSK9 CisMRBEE  0.69210856 0.02521962 8.360765e-166
## 7       TG  ANGPTL3 CisMVIVW  0.09936909 0.01088816  7.085000e-20
## 8       TG    APOA1 CisMVIVW  0.04819148 0.02839766  8.969227e-02
## 9       TG    APOC1 CisMVIVW  0.08455439 0.04700119  7.202123e-02
## 10      TG    APOA5 CisMVIVW -0.09278218 0.02807592  9.508192e-04
## 11      TG    APOC3 CisMVIVW  0.05224150 0.02564776  4.166173e-02
```

```
## 12      TG    PCSK9 CisMVIVW  0.08322063 0.03299576  1.166361e-02
## 13      TG  ANGPTL3    PCGMM  0.06592140 0.01780126  2.129067e-04
## 14      TG    APOA1    PCGMM  0.03337197 0.05142052  5.163377e-01
## 15      TG    APOC1    PCGMM  0.16494139 0.08419368  5.010449e-02
## 16      TG    APOA5    PCGMM -0.13517211 0.04742082  4.365326e-03
## 17      TG    APOC3    PCGMM  0.06419960 0.04107333  1.180412e-01
## 18      TG    PCSK9    PCGMM  0.17398527 0.05810955  2.752640e-03
```

Finally, we generate the visualization as shown below. It should be noted that we use the Bonferroni correction to calculate the confidence intervals here: that is, the width of the confidence interval is \sqrt{\text{qchisq}(0.05/p,1,\text{lower.tail=F})}\times\text{SE} instead of 2SE:

```
library(ggplot2)
library(corrplot)
Method=c("CisMRBEE","CisMVIVW","PCGMM")
LDLplot=data.frame(by=ZY[,"LDL"]/sqrt(NY[,"LDL"]),
          hatby=ANGPTL3_LDL$fitMRBEE$bXest%*%ANGPTL3_LDL$fitMRBEE$theta,
      pleiotropy=ifelse(ANGPTL3_LDL$fitMRBEE$gamma!=0,
                        names(ANGPTL3_LDL$fitMRBEE$gamma!=0),NA),
      Type="Marginal Effect",
      LD2=R[,which(ANGPTL3_LDL$fitMRBEE$gamma!=0)]^2)
HDLplot=data.frame(by=ZY[,"HDL"]/sqrt(NY[,"HDL"]),
          hatby=ANGPTL3_HDL$fitMRBEE$bXest%*%ANGPTL3_HDL$fitMRBEE$theta,
      pleiotropy=ifelse(ANGPTL3_HDL$fitMRBEE$gamma!=0,
                        names(ANGPTL3_HDL$fitMRBEE$gamma!=0),NA),
      Type="Marginal Effect",
      LD2=R[,which(ANGPTL3_HDL$fitMRBEE$gamma!=0)]^2)
TGplot=data.frame(by=ZY[,"TG"]/sqrt(NY[,"TG"]),
          hatby=ANGPTL3_TG$fitMRBEE$bXest%*%ANGPTL3_TG$fitMRBEE$theta,
      pleiotropy=ifelse(ANGPTL3_TG$fitMRBEE$gamma!=0,
                        names(ANGPTL3_TG$fitMRBEE$gamma!=0),NA),
      Type="Marginal Effect",
      LD2=R[,which(ANGPTL3_TG$fitMRBEE$gamma!=0)]^2)
LDL$Trait="LDL Cholesterol"
HDL$Trait="HDL Cholesterol"
TG$Trait="Triglycerides"
LDLplot$Trait="LDL Cholesterol"
HDLplot$Trait="HDL Cholesterol"
TGplot$Trait="Triglycerides"

DF1=do.call(rbind,list(LDL,HDL,TG))
DF2=do.call(rbind,list(LDLplot,HDLplot,TGplot))
DF1$Trait=ordered(DF1$Trait,levels=c("LDL Cholesterol","HDL Cholesterol","Triglycerides"))
DF2$Trait=ordered(DF2$Trait,levels=c("LDL Cholesterol","HDL Cholesterol","Triglycerides"))
DF1$Method=ordered(DF1$Method,levels=Method)

ggplot(DF1,aes(y=Exposure,x=Estimate,fill=Method)) +
geom_bar(stat="identity",position=position_dodge(width=0.9),width=0.7,color="black") +
geom_errorbar(aes(xmin=Estimate-2.638257*SE,xmax=Estimate+2.638257*SE),
position=position_dodge(width=0.9),width=0.25) +
geom_vline(xintercept=0,linetype="solid",color="black")+
scale_fill_manual(values=c("#ee2560","#f9d423","#45d9fd"))+
facet_grid(Trait~.)+
labs(y="causal effect estimate", fill=NULL)+
theme(axis.title.y=element_blank(),
```

```
legend.position=c(0.01, 0.99),
legend.justification=c(0.01, 0.99),
legend.direction="horizontal",
panel.background=element_blank(),
panel.border=element_rect(colour="black",fill=NA),
panel.grid=element_blank())+
ggtitle("A. Causal Effect Estimate of Multivariable Cis-Mendelian Randomization for Lipid Tratis")
```

```
## Warning: A numeric `legend.position` argument in `theme()` was deprecated in ggplot2
## 3.5.0.
## i Please use the `legend.position.inside` argument of `theme()` instead.
## This warning is displayed once every 8 hours.
## Call `lifecycle::last_lifecycle_warnings()` to see where this warning was
## generated.
```

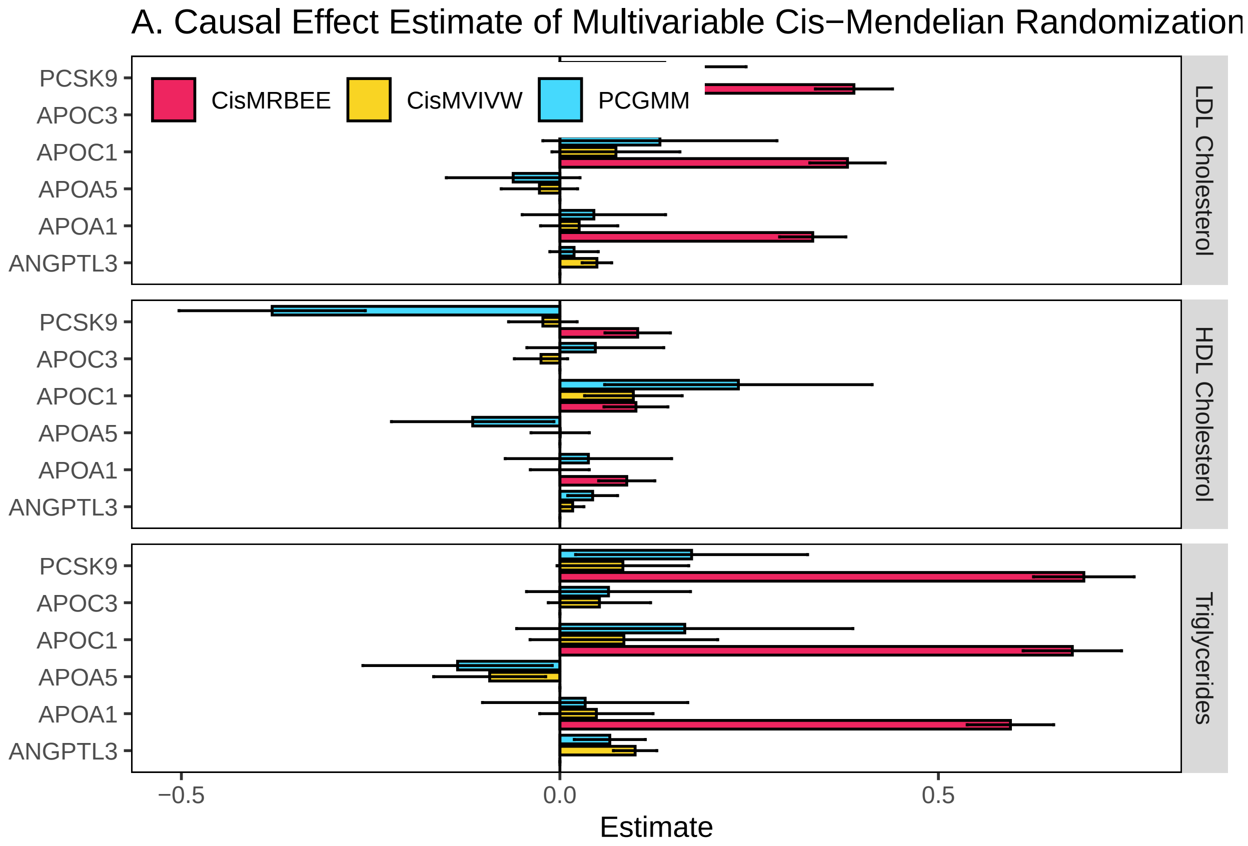


```
ggplot(DF2, aes(x = hatby, y = by, fill = sqrt(LD2))) +
geom_point(shape=21,color="grey70",size=4) +
facet_grid(~Trait)+
labs(x = "linear predictor of outcome GWAS effect", y = "outcome GWAS effect", fill ="absolute value of
theme_bw() +
scale_fill_gradient(low="#F7FBFF",high=corrplot::COL1("Blues"))+
theme(legend.position = "bottom",
legend.direction = "horizontal",
panel.background = element_blank(),
panel.border = element_rect(colour = "black", fill = NA),
panel.grid = element_blank())+
```

```
scale_x_continuous(limits=c(-0.03,0.02),breaks=seq(-0.03,0.02,0.01))+
ggtitle("B. Model Fitting of Multivariable Cis-Mendelian Randomization for Lipid Tratis")+
guides(size="none")+
geom_text(
data = DF2[!is.na(DF2$pleiotropy), ],
aes(label = pleiotropy),
hjust = -0.1, vjust = 0.5, size = 4, color = "black"
)
```

```
## Warning: Removed 8 rows containing missing values or values outside the scale range
## (`geom_point()`).
```

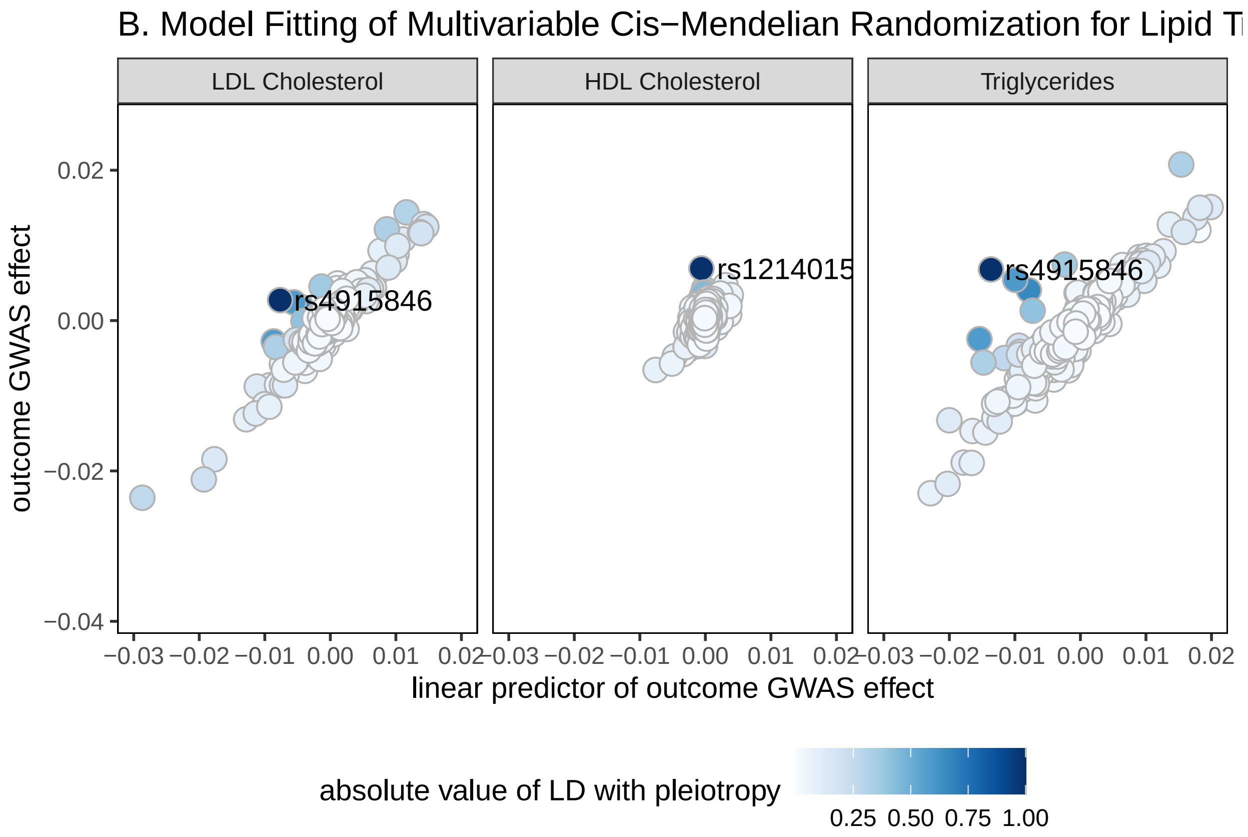

1.3 Step-by-step analysis of CR1

Next, we repeat the analysis for CR1. We skip the steps that are identical to those performed for ANGPTL3 (only presenting the codes), and only highlight the steps that differ from the ANGPTL3 analysis.

```
variant=readRDS("RDS/CR1_variant.rds")%>%as.data.frame(.)
datalist=readRDS("RDS/CR1_datalist.rds")
ZMatrix=matrix(0,dim(datalist[[1]])[1],length(datalist))
NMatrix=matrix(0,dim(datalist[[1]])[1],length(datalist))
for(i in 1:length(datalist)){
ZMatrix[,i]=datalist[[i]]$Zscore
NMatrix[,i]=datalist[[i]]$N
}
rownames(ZMatrix)=rownames(NMatrix)=datalist[[1]]$SNP
```

```
colnames(ZMatrix)=colnames(NMatrix)=names(datalist)
```

Since genome-wide eQTL and sceQTL summary data were unavailable, we used non-significant SNPs within the locus to estimate the covariance matrix of the estimation error. We defined non-significance more strictly, as having an absolute Z-score below 1.5, and relaxed the resampling proportion to 0.2.

```
Rxy=MRBEE::errorCov(ZMatrix,Zscore.cutoff=1.5,subsampling.ratio=0.2,subsampling.time=100)
```

```
##   |                                                                              |
```

```
colnames(Rxy)=rownames(Rxy)=names(datalist)
```

Next, we loaded the pre-stored IVs selected under the same conditions as ANGPTL3.

```
UKBBGenotype=readRDS("RDS/CR1_LDref.rds")
IVlist=readRDS("RDS/CR1_IVlist.rds")
R=cor(UKBBGenotype)
R[is.na(R)]=0;diag(R)=1
R=TGVIS::poet_shrinkage(R)
R=(t(R)+R)/2
genosnp=colnames(UKBBGenotype)
rownames(R)=colnames(R)=genosnp
ZY=ZMatrix[genosnp,1:3]
ZX=ZMatrix[genosnp,-c(1:3)]
NY=NMatrix[genosnp,1:3]
NX=NMatrix[genosnp,-c(1:3)]
```

The subsequent analysis followed the same approach as that for ANGPTL3.

```
by=ZY[,"AD"]/sqrt(NY[,"AD"])
byse=1/sqrt(NY[,"AD"])
bX=ZX/sqrt(NX)
bXse=1/sqrt(NX)
NAM=c(colnames(bX),"AD")
MVINPUT=mr_mvinput(bx=bX,by=by,bxse=bXse,byse=byse,correlation=R)
fitMRBEE=MRBEEX::CisMRBEEX(causal.pip.thres=0.2,by,bX,byse,bXse,LD=R,Rxy=Rxy[NAM,NAM],
                           reliability.thres=0.8,xQTL.max.L=15,xQTL.pip.min=0.2,
                           xQTL.pip.thres=0.3,xQTL.Nvec=colMeans(NX),
                           tauvec=seq(4.5,30,1.5),susie.iter=500,ridge.diff=100)
```

```
## Please standardize data such that BETA = Zscore/sqrt n and SE = 1/sqrt n
## Sparse prediction ends: 0.569 secs
## Causal effect estimation ends: 0.609 secs
```

```
fitCisIVW=mr_mvivw(MVINPUT,correl=T)
fitPCGMM=mr_mvpcgmm(MVINPUT,nx=colMeans(NX),ny=mean(NY[,"AD"]),thres=0.99)
CR1_AD=list(fitMRBEE=fitMRBEE,fitCisIVW=fitCisIVW,fitPCGMM=fitPCGMM)
AD=data.frame(
Estimate=c(CR1_AD$fitMRBEE$theta,CR1_AD$fitCisIVW@Estimate,CR1_AD$fitPCGMM@Estimate),
SE=c(CR1_AD$fitMRBEE$theta.se,CR1_AD$fitCisIVW@StdError,CR1_AD$fitPCGMM@StdError),
Exposure=colnames(ZX),
Method=c(rep("CisMRBEE",9),rep("CisMVIVW",9),rep("PCGMM",9)))
AD$P=pchisq(AD$Estimate^2/AD$SE^2,1,lower.tail=F);AD$P[is.na(AD$P)]=1
AD$Outcome="AD"
AD=dplyr::select(AD,Outcome,Exposure,Method,Estimate,SE,P)
print(AD)
```

```
##    Outcome        Exposure   Method     Estimate          SE            P
## 1       AD     Endothelial CisMRBEE  0.000000000 0.000000000 1.000000e+00
## 2       AD       Microglia CisMRBEE  0.000000000 0.000000000 1.000000e+00
## 3       AD Oligodendrocytes CisMRBEE  0.017005194 0.001807402 5.025958e-21
## 4       AD       Pericytes CisMRBEE  0.000000000 0.000000000 1.000000e+00
## 5       AD          Cortex CisMRBEE  0.000000000 0.000000000 1.000000e+00
## 6       AD      Cerebellum CisMRBEE  0.000000000 0.000000000 1.000000e+00
## 7       AD     Hippocampus CisMRBEE  0.028133744 0.002992837 5.437665e-21
## 8       AD      Spinalcord CisMRBEE  0.000000000 0.000000000 1.000000e+00
## 9       AD    Basalganglia CisMRBEE  0.022894079 0.002434598 5.270909e-21
## 10      AD     Endothelial CisMVIVW  0.006291119 0.007203112 3.824511e-01
## 11      AD       Microglia CisMVIVW  0.001706126 0.006554807 7.946429e-01
## 12      AD Oligodendrocytes CisMVIVW -0.006426913 0.005439402 2.373852e-01
## 13      AD       Pericytes CisMVIVW -0.001364303 0.006460514 8.327502e-01
## 14      AD          Cortex CisMVIVW  0.060924484 0.011734937 2.083626e-07
## 15      AD      Cerebellum CisMVIVW -0.006325998 0.010938023 5.630283e-01
## 16      AD     Hippocampus CisMVIVW  0.003083597 0.007200469 6.684696e-01
## 17      AD      Spinalcord CisMVIVW  0.009181371 0.004359289 3.519021e-02
## 18      AD    Basalganglia CisMVIVW -0.002331931 0.006842095 7.332382e-01
## 19      AD     Endothelial    PCGMM  0.022819969 0.038809443 5.565320e-01
## 20      AD       Microglia    PCGMM  0.016490882 0.027569589 5.497366e-01
## 21      AD Oligodendrocytes    PCGMM -0.026743892 0.037353096 4.740066e-01
## 22      AD       Pericytes    PCGMM  0.049571581 0.050197627 3.233837e-01
## 23      AD          Cortex    PCGMM  0.048748146 0.064249627 4.480138e-01
## 24      AD      Cerebellum    PCGMM  0.004188827 0.070594955 9.526844e-01
## 25      AD     Hippocampus    PCGMM  0.038591027 0.057859558 5.047865e-01
## 26      AD      Spinalcord    PCGMM -0.003447299 0.032887480 9.165178e-01
## 27      AD    Basalganglia    PCGMM -0.018551303 0.039073876 6.349473e-01
```

```r
summary(fitMRBEE$susie.theta)
```

```
##
## Variables in credible sets:
##
##  variable variable_prob cs
##         9     0.3480059  1
##         7     0.3341938  1
##         3     0.3099593  1
##
## Credible sets summary:
##
##  cs cs_log10bf cs_avg_r2 cs_min_r2 variable
##   1   26.12152 0.9999388 0.9998922    3,7,9
```

```r
by=ZY[,"ABETA"]/sqrt(NY[,"ABETA"])
byse=1/sqrt(NY[,"ABETA"])
bX=ZX/sqrt(NX)
bXse=1/sqrt(NX)
NAM=c(colnames(bX),"ABETA")
MVINPUT=mr_mvinput(bx=bX,by=by,bxse=bXse,byse=byse,correlation=R)
fitMRBEE=MRBEEX::CisMRBEEX(causal.pip.thres=0.2,by,bX,byse,bXse,LD=R,Rxy=Rxy[NAM,NAM],
                           reliability.thres=0.8,xQTL.max.L=15,xQTL.pip.min=0.2,
                           xQTL.pip.thres=0.3,xQTL.Nvec=colMeans(NX),
                           tauvec=seq(4.5,30,1.5),susie.iter=500,ridge.diff=100)
```

```
## Please standardize data such that BETA = Zscore/sqrt n and SE = 1/sqrt n
## Sparse prediction ends: 0.673 secs
## Causal effect estimation ends: 0.624 secs
```

```
fitCisIVW=mr_mvivw(MVINPUT,correl=T)
fitPCGMM=mr_mvpcgmm(MVINPUT,nx=colMeans(NX),ny=mean(NY[,"ABETA"]),thres=0.99)
```

```
## overdispersion parameter could not be estimated by uniroot. Default value of 0 selected, and unrobust
```

```
CR1_ABETA=list(fitMRBEE=fitMRBEE,fitCisIVW=fitCisIVW,fitPCGMM=fitPCGMM)
ABETA=data.frame(
Estimate=c(CR1_ABETA$fitMRBEE$theta,CR1_ABETA$fitCisIVW@Estimate,
CR1_ABETA$fitPCGMM@Estimate),
SE=c(CR1_ABETA$fitMRBEE$theta.se,CR1_ABETA$fitCisIVW@StdError,
CR1_ABETA$fitPCGMM@StdError),
Exposure=colnames(ZX),
Method=c(rep("CisMRBEE",9),rep("CisMVIVW",9),rep("PCGMM",9)))
ABETA$P=pchisq(ABETA$Estimate^2/ABETA$SE^2,1,lower.tail=F);ABETA$P[is.na(ABETA$P)]=1
ABETA$Outcome="ABeta42"
ABETA=dplyr::select(ABETA,Outcome,Exposure,Method,Estimate,SE,P)
print(ABETA)
```

```
##    Outcome         Exposure   Method     Estimate          SE            P
## 1  ABeta42      Endothelial CisMRBEE  0.000000000 0.000000000 1.000000e+00
## 2  ABeta42        Microglia CisMRBEE  0.000000000 0.000000000 1.000000e+00
## 3  ABeta42 Oligodendrocytes CisMRBEE -0.039143195 0.005638254 3.854075e-12
## 4  ABeta42        Pericytes CisMRBEE  0.000000000 0.000000000 1.000000e+00
## 5  ABeta42           Cortex CisMRBEE  0.000000000 0.000000000 1.000000e+00
## 6  ABeta42       Cerebellum CisMRBEE  0.000000000 0.000000000 1.000000e+00
## 7  ABeta42      Hippocampus CisMRBEE -0.064874190 0.009341174 3.785355e-12
## 8  ABeta42       Spinalcord CisMRBEE  0.000000000 0.000000000 1.000000e+00
## 9  ABeta42     Basalganglia CisMRBEE -0.052745940 0.007598167 3.867812e-12
## 10 ABeta42      Endothelial CisMVIVW  0.030059686 0.030459305 3.237014e-01
## 11 ABeta42        Microglia CisMVIVW -0.002302069 0.029304068 9.373842e-01
## 12 ABeta42 Oligodendrocytes CisMVIVW  0.030712527 0.024393823 2.080199e-01
## 13 ABeta42        Pericytes CisMVIVW -0.038424777 0.028569789 1.786432e-01
## 14 ABeta42           Cortex CisMVIVW -0.072683390 0.050295027 1.484184e-01
## 15 ABeta42       Cerebellum CisMVIVW -0.051272503 0.048675806 2.921821e-01
## 16 ABeta42      Hippocampus CisMVIVW -0.001982042 0.032259391 9.510082e-01
## 17 ABeta42       Spinalcord CisMVIVW -0.021579835 0.018414913 2.412503e-01
## 18 ABeta42     Basalganglia CisMVIVW -0.021550767 0.031007045 4.870377e-01
## 19 ABeta42      Endothelial    PCGMM  0.511271756 2.409544915 8.319619e-01
## 20 ABeta42        Microglia    PCGMM -0.433629094 1.725298461 8.015546e-01
## 21 ABeta42 Oligodendrocytes    PCGMM  0.388190774 1.294388341 7.642512e-01
## 22 ABeta42        Pericytes    PCGMM  0.317197623 1.981255500 8.728029e-01
## 23 ABeta42           Cortex    PCGMM  0.528639254 2.980903478 8.592398e-01
## 24 ABeta42       Cerebellum    PCGMM -0.818813022 3.890964493 8.333246e-01
## 25 ABeta42      Hippocampus    PCGMM -0.775326733 3.181046722 8.074375e-01
## 26 ABeta42       Spinalcord    PCGMM  0.599011352 2.775205649 8.291094e-01
## 27 ABeta42     Basalganglia    PCGMM -0.219769950 1.148280193 8.482196e-01
```

```
summary(fitMRBEE$susie.theta)
```

```
##
## Variables in credible sets:
##
```

```
##  variable variable_prob cs
##         9     0.3331018  1
##         7     0.3200418  1
##         3     0.3071714  1
##
## Credible sets summary:
##
##  cs cs_log10bf cs_avg_r2 cs_min_r2 variable
##   1   10.03463 0.9999703 0.9999478    3,7,9
```

```
by=ZY[,"Tau"]/sqrt(NY[,"Tau"])
byse=1/sqrt(NY[,"Tau"])
bX=ZX/sqrt(NX)
bXse=1/sqrt(NX)
NAM=c(colnames(bX),"Tau")
MVINPUT=mr_mvinput(bx=bX,by=by,bxse=bXse,byse=byse,correlation=R)
fitMRBEE=MRBEEX::CisMRBEEX(causal.pip.thres=0.2,by,bX,byse,bXse,LD=R,Rxy=Rxy[NAM,NAM],
                           reliability.thres=0.8,xQTL.max.L=15,xQTL.pip.min=0.2,
                           xQTL.pip.thres=0.3,xQTL.Nvec=colMeans(NX),
                           tauvec=seq(4.5,30,1.5),susie.iter=500,ridge.diff=100)
```

```
## Please standardize data such that BETA = Zscore/sqrt n and SE = 1/sqrt n
## Sparse prediction ends: 0.578 secs
## Causal effect estimation ends: 0.969 secs
```

```
fitCisIVW=mr_mvivw(MVINPUT,correl=T)
fitPCGMM=mr_mvpcgmm(MVINPUT,nx=colMeans(NX),ny=mean(NY[,"Tau"]),thres=0.99)
CR1_Tau=list(fitMRBEE=fitMRBEE,fitCisIVW=fitCisIVW,fitPCGMM=fitPCGMM)
Tau=data.frame(
Estimate=c(CR1_Tau$fitMRBEE$theta,CR1_Tau$fitCisIVW@Estimate,CR1_Tau$fitPCGMM@Estimate),
SE=c(CR1_Tau$fitMRBEE$theta.se,CR1_Tau$fitCisIVW@StdError,CR1_Tau$fitPCGMM@StdError),
Exposure=colnames(ZX),
Method=c(rep("CisMRBEE",9),rep("CisMVIVW",9),rep("PCGMM",9)))
Tau$P=pchisq(Tau$Estimate^2/Tau$SE^2,1,lower.tail=F);Tau$P[is.na(Tau$P)]=1
Tau$Outcome="pTau"
Tau=dplyr::select(Tau,Outcome,Exposure,Method,Estimate,SE,P)
print(Tau)
```

```
##    Outcome        Exposure   Method      Estimate          SE            P
## 1     pTau      Endothelial CisMRBEE  0.0000000000 0.000000000 1.000000e+00
## 2     pTau        Microglia CisMRBEE  0.0000000000 0.000000000 1.000000e+00
## 3     pTau Oligodendrocytes CisMRBEE  0.0397158514 0.007098682 2.208501e-08
## 4     pTau        Pericytes CisMRBEE  0.0000000000 0.000000000 1.000000e+00
## 5     pTau           Cortex CisMRBEE  0.0000000000 0.000000000 1.000000e+00
## 6     pTau       Cerebellum CisMRBEE  0.0000000000 0.000000000 1.000000e+00
## 7     pTau      Hippocampus CisMRBEE  0.0657396633 0.011755542 2.241749e-08
## 8     pTau       Spinalcord CisMRBEE  0.0000000000 0.000000000 1.000000e+00
## 9     pTau     Basalganglia CisMRBEE  0.0535709089 0.009562127 2.113976e-08
## 10    pTau      Endothelial CisMVIVW -0.0383022390 0.025365462 1.310395e-01
## 11    pTau        Microglia CisMVIVW  0.0007226599 0.024414571 9.763864e-01
## 12    pTau Oligodendrocytes CisMVIVW -0.0204492971 0.020304927 3.138819e-01
## 13    pTau        Pericytes CisMVIVW  0.0101075019 0.023665103 6.693023e-01
## 14    pTau           Cortex CisMVIVW  0.1440026298 0.043978031 1.058784e-03
## 15    pTau       Cerebellum CisMVIVW -0.0143293888 0.040736742 7.250213e-01
## 16    pTau      Hippocampus CisMVIVW -0.0127357339 0.026774531 6.343124e-01
```

```
## 17    pTau       Spinalcord CisMVIVW  0.0411157146 0.015296660 7.190544e-03
## 18    pTau     Basalganglia CisMVIVW -0.0384174241 0.025819991 1.367787e-01
## 19    pTau      Endothelial    PCGMM  0.0434217545 0.102701723 6.724450e-01
## 20    pTau        Microglia    PCGMM -0.0390425304 0.071968244 5.874764e-01
## 21    pTau Oligodendrocytes    PCGMM -0.1166911468 0.088500517 1.873242e-01
## 22    pTau        Pericytes    PCGMM  0.0605954577 0.127857713 6.355507e-01
## 23    pTau           Cortex    PCGMM  0.2083212716 0.171002444 2.231344e-01
## 24    pTau       Cerebellum    PCGMM -0.0381126292 0.186921281 8.384342e-01
## 25    pTau      Hippocampus    PCGMM -0.0427898471 0.151992623 7.783071e-01
## 26    pTau       Spinalcord    PCGMM  0.0568049139 0.087466161 5.160474e-01
## 27    pTau     Basalganglia    PCGMM  0.0103258977 0.101310475 9.188176e-01
```

```
summary(fitMRBEE$susie.theta)
```

```
##
## Variables in credible sets:
##
##  variable variable_prob cs
##         9     0.2736355  1
##         3     0.2604002  1
##         7     0.2573103  1
##         5     0.1926711  1
##
## Credible sets summary:
##
##  cs cs_log10bf cs_avg_r2 cs_min_r2 variable
##   1    8.19166 0.9591113 0.9190974  3,5,7,9
```

2 Simulation

First, we introduce the basic setup of our simulation. We define two functions to generate the genetic correlation matrix for effect sizes and the estimation error:

```
rm(list=ls())
library(MendelianRandomization)
ARcov=function(p,rho){
s=c(1:p)
for(i in 1:p){
s[i]=rho^(i-1)
}
return(toeplitz(s))
}
CScov=function(p,rho){
return(matrix(rho,p,p)+(1-rho)*diag(p))
}
```

The other settings with brief descriptions are as follows:

```
m=200 # number of IVs
p=10 # number of exposure
n1=30000 # exposure sample size
n0=0.5e6 # outcome sample size
pca.thres=0.999 # PCGMM threshold
pip.thres=0.3 # PIP threshold of SuSiE in cisMRBEE
ridge=0
```

```
Rbb=ARcov(p,-0.5) # exposure covariance
Ruv=ARcov(p+1,-0.3) # estimation error covariance
Nxy=c(rep(n1,p),n0) # sample size vector
Hxy=c(rep(.3,p),.001) # H2 vector for expsoures and outcome
Rnn=matrix(1,p+1,p+1) # sample overlap matrix
#We assume the cohorts of exposures are the subset of the cohort of outcome
theta0=c(1,0,-0.5,rep(0,p-3)) # true causal effect vector
UHP.var=1 # var(exposure)/var(UHP)=UHP.var
UHP.frac=0.01 # generate m x UHP.frac number of UHP
xQTL.pip.thres=0.5 # the PIP threshold of xQTL effect estimate
```

We selected the top 10 regions from the Height GWAS, with each region centered on the most significant Height signal and a 1 MB radius. SNPs were extracted using `--clump-kb 1000 --clump-p1 5e-5 --clump-p2 5e-5 --clump-r2 0.81`, ensuring they are moderately significant and not highly correlated. Each block contains approximately 200 IVs, resulting in a total dimension of 2083. For each simulation, 200 contiguous IVs are randomly selected by index. These 200-dimensional IVs are used as the LD matrix for the corresponding cis region:

```
LD0=readRDS("RDS/LD.rds")%>%do.call(Matrix::bdiag,.)%>%as.matrix(.)
ldindex=sample(floor(2083/m),1,replace=F)
ldindex=c(((ldindex-1)*m+1):(ldindex*m))
LD1=LD0;diag(LD1)=0;
print(c(max(LD1),min(LD1)))
```

```
## [1]  0.8980549 -0.9008342
```

```
# The maximum correlation is 0.9
LD=LD0[ldindex,ldindex]*0.75+0.25*diag(m)
# As the correlation may be very high, we consider a linear shrinkage
# of the true LD matrix
```

We first use the `summary_generation` included in `MRBEEX` to generate the GWAS summary data directly. We do not consider the CHP here and hence put `CHP.frac=0`.

```
A=MRBEEX::summary_generation(theta=theta0,m=m,Rbb=Rbb,Ruv=Ruv,Rnn=Rnn,LD=LD,
                   Nxy=Nxy,non.zero.frac=rep(0.005,p),UHP.frac=UHP.frac,
                   CHP.frac=0,UHP.var=UHP.var,Hxy=Hxy,UHP.dis="normal")
bX=A$bX
by=A$by
if(sum(is.na(by))>0){
next
}
bXse=A$bXse
byse=A$byse
Rxy=Rnn*Ruv
```

Then we implement cis-MVIVW, PCGMM, MRBEE-IPOD, cis-MRBEE, and cis-MVIVW(adjusted) sequentially:

```
MVINPUT=mr_mvinput(bx=bX,bxse=bXse,by=by,byse=byse,correlation=LD)
t1=Sys.time()
fit.ivw=mr_mvivw(MVINPUT,correl=T)
t2=Sys.time()
ivw.time=difftime(t2, t1, units = "secs")

t1=Sys.time()
```

```r
fit.pca=mr_mvpcgmm(MVINPUT,ny=n0,nx=rep(n1,p),thres=pca.thres)
t2=Sys.time()
pca.time=difftime(t2, t1, units = "secs")

t1=Sys.time()
fit.cismrbee=CisMRBEEX(by=by,bX=bX,byse=byse,bXse=bXse,xQTL.max.L=5,
                       xQTL.pip.thres=xQTL.pip.thres,xQTL.Nvec=rep(n1,p),LD=LD,
                       Rxy=Rnn*Ruv,reliability.thres=0.9,tauvec=seq(2,15,1),
                       ebic.gamma=2,ebic.theta=0,ridge=ridge,
                       causal.pip.thres=pip.thres)
```

```
## Please standardize data such that BETA = Zscore/sqrt n and SE = 1/sqrt n
## Sparse prediction ends: 1.308 secs
## Causal effect estimation ends: 2.139 secs
```

```r
t2=Sys.time()
susie.twas=difftime(t2, t1, units = "secs")

t1=Sys.time()
fit.ipod=Cis_MRBEE_IPOD(by=by,bX=bX,byse=byse,bXse=bXse,LD=LD,Rxy=Rnn*Ruv,
                      reliability.thres=0.9,tauvec=seq(2,15,1),
                      ebic.gamma=2)
t2=Sys.time()
ipod.time=difftime(t2, t1, units = "secs")

MVINPUT=mr_mvinput(bx=fit.cismrbee$bXest,bxse=fit.cismrbee$bXestse,by=by,
                   byse=byse,correlation=LD)
t1=Sys.time()
twas.ivw=mr_mvivw(MVINPUT,correl=T)
t2=Sys.time()
ivw.twas=difftime(t2, t1, units = "secs")

multiplefactor=colMeans(bXse)/mean(byse)
fittgfm=TGVIS::tgfm(by=by/byse,bX=bX/bXse,LD=LD,Nvec=c(rep(n1,p),n0),L.eqtl=5,
                    L.causal=5,eqtl.sampling.time=25,causal.sampling.time=50)

fittgvis=TGVIS::tgvis(by=by/byse,bXest=fit.cismrbee$bXest0/bXse,LD=LD,Noutcome=n0,
                    L.causal.vec=c(1:4),eigen.thres=1,pv.thres=5e-3,
                    ebic.beta=0,susie.iter=500)

BETAEst=cbind(fit.cismrbee$theta,fit.ipod$theta,fit.ivw@Estimate,fit.pca@Estimate,
              fit.ivw@Estimate,twas.ivw@Estimate,fittgfm$theta/multiplefactor,
              fittgvis$theta/multiplefactor)
BETASE=cbind(fit.cismrbee$theta.se,fit.ipod$theta.se,fit.ivw@StdError,
             fit.pca@StdError,twas.ivw@StdError)
BETAPIP=cbind(fit.cismrbee$theta.pip,fittgfm$theta.pip,fittgvis$theta.pip)
print(BETAEst)
```

```
##             [,1]         [,2]         [,3]         [,4]         [,5]
## [1,]  1.0198422  1.008451145  0.988137025  1.021855312  0.988137025
## [2,]  0.0000000 -0.010716547 -0.107886467 -0.118215193 -0.107886467
## [3,] -0.4556495 -0.485427985 -0.482324060 -0.497262492 -0.482324060
## [4,]  0.0000000 -0.005210742 -0.001961802  0.005002905 -0.001961802
## [5,]  0.0000000  0.015377045 -0.085682182 -0.107587208 -0.085682182
```

```
##  [6,]  0.0000000 -0.022714642 -0.026551083 -0.015613525 -0.026551083
##  [7,]  0.0000000  0.037406268  0.146783110  0.075804949  0.146783110
##  [8,]  0.0000000 -0.083824641 -0.079062467 -0.095918519 -0.079062467
##  [9,]  0.0000000  0.026320202  0.015127603  0.012049740  0.015127603
## [10,]  0.0000000 -0.094167947 -0.142991134 -0.163718240 -0.142991134
##               [,6]       [,7]       [,8]
##  [1,]  1.012794278  1.1183534  0.9689447
##  [2,] -0.111485024  0.0000000  0.0000000
##  [3,] -0.465730700 -0.4886888 -0.4473305
##  [4,] -0.005397643  0.0000000  0.0000000
##  [5,] -0.058224263  0.0000000  0.0000000
##  [6,] -0.019889496  0.0000000  0.0000000
##  [7,]  0.166366723  0.0000000  0.0000000
##  [8,] -0.095206883  0.0000000  0.0000000
##  [9,]  0.050142301  0.0000000  0.0000000
## [10,] -0.123403369  0.0000000  0.0000000
```

```
print(BETASE)
```

```
##             [,1]       [,2]       [,3]       [,4]       [,5]
##  [1,] 0.06609084 0.06915689 0.10504641 0.11656153 0.10576688
##  [2,] 0.00000000 0.06448137 0.09768900 0.09422663 0.09803374
##  [3,] 0.06370095 0.07235107 0.11002219 0.10543667 0.11114150
##  [4,] 0.00000000 0.07241489 0.11006751 0.10997339 0.11066977
##  [5,] 0.00000000 0.06590598 0.09990425 0.10089984 0.10018178
##  [6,] 0.00000000 0.06813812 0.10345906 0.11463306 0.10419063
##  [7,] 0.00000000 0.06668659 0.10065608 0.09515952 0.10105241
##  [8,] 0.00000000 0.06806541 0.10352618 0.09829193 0.10392125
##  [9,] 0.00000000 0.06622615 0.10110480 0.09368762 0.10114190
## [10,] 0.00000000 0.06585468 0.10047073 0.11140954 0.10114168
```

```
print(BETAPIP)
```

```
##               [,1]      [,2]         [,3]
##  [1,] 1.000000e+00 0.9664620 9.509519e-01
##  [2,] 5.116796e-12 0.0000000 6.415535e-12
##  [3,] 1.000000e+00 0.9556709 1.000000e+00
##  [4,] 6.460791e-10 0.0000000 7.821209e-10
##  [5,] 6.192269e-12 0.0000000 7.571388e-12
##  [6,] 7.130074e-12 0.0000000 9.569345e-12
##  [7,] 6.089018e-12 0.0000000 7.427392e-12
##  [8,] 4.213674e-11 0.0000000 5.223033e-11
##  [9,] 9.102052e-12 0.0000000 1.141465e-11
## [10,] 1.001621e-11 0.0000000 1.327216e-11
```

2.1 Examination of discrete differential penalty

We next evaluate whether the proposed discrete differential penalty effectively handles the estimation of causal effects for likely duplicated exposures within the same credible set. The setup is similar to the main simulation described above, but we consider only $\boldsymbol{\theta} = c(1, 0, \ldots, 0)^\top$, meaning that only the first variable has a causal effect.

```
m=200
p=10
n1=30000
pip.thres=0.3
```

```r
ridge=0
n0=0.5e6
Rbb=ARcov(p,-0.5)
Ruv=ARcov(p+1,-0.3)
Nxy=c(rep(n1,p),n0)
Hxy=c(rep(.3,p),.001)
Rnn=matrix(1,p+1,p+1)
theta0=c(1,0,rep(0,p-2))
UHP.var=1
UHP.frac=0.00
xQTL.pip.thres=0.5
LD0=readRDS("RDS/LD.rds")%>%do.call(Matrix::bdiag,.)%>%as.matrix(.)
ldindex=sample(floor(2083/m),1,replace=F)
ldindex=c(((ldindex-1)*m+1):(ldindex*m))
LD=LD0[ldindex,ldindex]*0.75+0.25*diag(m)
A=MRBEEX::summary_generation(theta=theta0,m=m,Rbb=Rbb,Ruv=Ruv,Rnn=Rnn,LD=LD,
                             Nxy=Nxy,non.zero.frac=rep(0.015,p),UHP.frac=UHP.frac,
                             CHP.frac=0,UHP.var=UHP.var,Hxy=Hxy,UHP.dis="normal")
bX=A$bX
by=A$by
if(sum(is.na(by))>0){
next
}
bXse=A$bXse
byse=A$byse
Rxy=Rnn*Ruv
```

We consider three scenarios: (1) the first two exposures, (2) the first three exposures, and (3) the first four exposures share the same underlying genetic component but differ in their estimation errors. We apply cis-MRBEE to these three scenarios:

```r
bX[,2]=LD%*%A$bX0[,1]+(A$bX[,2]-LD%*%A$bX0[,2])
fit.cismrbee1=CisMRBEEX(by=by,bX=bX,byse=byse,bXse=bXse,xQTL.max.L=5,
                        xQTL.pip.thres=xQTL.pip.thres,xQTL.Nvec=rep(n1,p),LD=LD,
                        Rxy=Rnn*Ruv,reliability.thres=0.9,tauvec=seq(3,15,1.5),
                        ebic.gamma=2,causal.pip.thres=0.2,ridge.diff=10)
```

```
## Please standardize data such that BETA = Zscore/sqrt n and SE = 1/sqrt n
## Sparse prediction ends: 1.108 secs
## Causal effect estimation ends: 0.562 secs
```

```r
bX[,3]=LD%*%A$bX0[,1]+(A$bX[,3]-LD%*%A$bX0[,3])
fit.cismrbee2=CisMRBEEX(by=by,bX=bX,byse=byse,bXse=bXse,xQTL.max.L=5,
                        xQTL.pip.thres=xQTL.pip.thres,xQTL.Nvec=rep(n1,p),LD=LD,
                        Rxy=Rnn*Ruv,reliability.thres=0.9,tauvec=seq(3,15,1.5),
                        ebic.gamma=2,causal.pip.thres=0.2,ridge.diff=10)
```

```
## Please standardize data such that BETA = Zscore/sqrt n and SE = 1/sqrt n
## Sparse prediction ends: 1.007 secs
## Causal effect estimation ends: 0.545 secs
```

```r
bX[,4]=LD%*%A$bX0[,1]+(A$bX[,4]-LD%*%A$bX0[,4])
fit.cismrbee3=CisMRBEEX(by=by,bX=bX,byse=byse,bXse=bXse,xQTL.max.L=5,
                        xQTL.pip.thres=xQTL.pip.thres,xQTL.Nvec=rep(n1,p),LD=LD,
                        Rxy=Rnn*Ruv,reliability.thres=0.9,tauvec=seq(3,15,1.5),
                        ebic.gamma=2,causal.pip.thres=0.2,ridge.diff=10)
```

```
## Please standardize data such that BETA = Zscore/sqrt n and SE = 1/sqrt n
## Sparse prediction ends: 0.97 secs
## Causal effect estimation ends: 0.692 secs
```

```
print(cbind(fit.cismrbee1$theta,fit.cismrbee2$theta,fit.cismrbee3$theta))
```

```
##             [,1]      [,2]      [,3]
##  [1,] 0.5409323 0.3606404 0.2705648
##  [2,] 0.5370581 0.3580508 0.2686251
##  [3,] 0.0000000 0.3631818 0.2724699
##  [4,] 0.0000000 0.0000000 0.2644317
##  [5,] 0.0000000 0.0000000 0.0000000
##  [6,] 0.0000000 0.0000000 0.0000000
##  [7,] 0.0000000 0.0000000 0.0000000
##  [8,] 0.0000000 0.0000000 0.0000000
##  [9,] 0.0000000 0.0000000 0.0000000
## [10,] 0.0000000 0.0000000 0.0000000
```

We next examine the significance of single effects represented by each credible set, rather than the significance of individual causal effects. Specifically, we consider the following single effects:

$$\hat{\theta}_{\text{scenario}(1)} = \sum_{j=1}^{2} \hat{\theta}_j, \ \hat{\theta}_{\text{scenario}(2)} = \sum_{j=1}^{3} \hat{\theta}_j, \ \hat{\theta}_{\text{scenario}(3)} = \sum_{j=1}^{4} \hat{\theta}_j.$$

In the simulations, the variance of exposures is standardized to 1, allowing us to directly sum the estimates. In real data applications, however, adjustments for exposure scales would be necessary. The variances of these three single effects are given by:

$$\text{var}(\hat{\theta}_{\text{scenario(k)}}) = \mathbf{1}_k^\top \text{cov}(\hat{\boldsymbol{\theta}}_k) \mathbf{1}_k, \quad k = 2, 3, 4,$$

where $\mathbf{1}_k$ is a vector of ones with length k, and $\text{cov}(\hat{\boldsymbol{\theta}}_k)$ is the covariance matrix of the first k components of $\hat{\boldsymbol{\theta}}$.

```
a=c(1,1)
b=c(1,1,1)
d=c(1,1,1,1)
theta1=sum(fit.cismrbee1$theta[1:2]);theta1.se=sqrt(sum(a*(fit.cismrbee1$theta.cov[1:2,1:2])%*%a))
theta2=sum(fit.cismrbee2$theta[1:3]);theta2.se=sqrt(sum(b*(fit.cismrbee2$theta.cov[1:3,1:3])%*%b))
theta3=sum(fit.cismrbee3$theta[1:4]);theta3.se=sqrt(sum(d*(fit.cismrbee3$theta.cov[1:4,1:4])%*%d))
print(cbind(c(theta1,theta2,theta3),c(theta1.se,theta2.se,theta3.se)))
```

```
##          [,1]       [,2]
## [1,] 1.077990 0.06350216
## [2,] 1.081873 0.06372425
## [3,] 1.076092 0.06336703
```

2.2 Simulations of Maximum correlation of LD

We investigate the impact of different LD correlations on cis-MRBEE. By introducing a shrinkage coefficient a, we define the adjusted LD matrix as $\mathbf{R}\text{actual} = a\mathbf{R}\text{original} + (1-a)\mathbf{I}$. This allows us to study the dynamic changes in cis-MRBEE estimates as LD decreases. The general settings are the same as the previous ones:

```
m=200
p=10
n1=30000
```

```
pip.thres=0.3
ridge=0
n0=0.5e6
Rbb=ARcov(p,-0.5)
Ruv=ARcov(p+1,-0.3)
Nxy=c(rep(n1,p),n0)
Hxy=c(rep(.3,p),.001)
Rnn=matrix(1,p+1,p+1)
theta0=c(1,0,0.5,rep(0,p-3))
UHP.var=1
UHP.frac=0.00
xQTL.pip.thres=0.5
```

We change the shrinkage coefficient a from 1 to 0.5 and perform cis-MRBEE on the corresponding data:

```
ldindex=sample(floor(2083/m),1,replace=F)
ldindex=c(((ldindex-1)*m+1):(ldindex*m))

LD1=LD0[ldindex,ldindex]
A=MRBEEX::summary_generation(theta=theta0,m=m,Rbb=Rbb,Ruv=Ruv,Rnn=Rnn,
                             LD=LD1,Nxy=Nxy,non.zero.frac=rep(0.015,p),
                             UHP.frac=UHP.frac,CHP.frac=0,UHP.var=UHP.var,
                             Hxy=Hxy,UHP.dis="normal")
bX=A$bX
by=A$by
if(sum(is.na(by))>0){
next
}
bXse=A$bXse
byse=A$byse
Rxy=Rnn*Ruv

fit.cismrbee1=CisMRBEEX(by=by,bX=bX,byse=byse,bXse=bXse,xQTL.max.L=5,
                      xQTL.Nvec=rep(n1,p),LD=LD1,Rxy=Rnn*Ruv,
                      reliability.thres=0.9,tauvec=seq(2,15,1),
                      ebic.gamma=2,ebic.theta=0,ridge=ridge,causal.pip.thres=pip.thres)
```

```
## Please standardize data such that BETA = Zscore/sqrt n and SE = 1/sqrt n
## Sparse prediction ends: 8.172 secs
## Causal effect estimation ends: 2.131 secs
```

```
t2=Sys.time()


LD2=LD0[ldindex,ldindex]*0.9+diag(m)*0.1
A=MRBEEX::summary_generation(theta=theta0,m=m,Rbb=Rbb,Ruv=Ruv,Rnn=Rnn,
                             LD=LD2,Nxy=Nxy,non.zero.frac=rep(0.015,p),
                             UHP.frac=UHP.frac,CHP.frac=0,UHP.var=UHP.var,
                             Hxy=Hxy,UHP.dis="normal")
bX=A$bX
by=A$by
if(sum(is.na(by))>0){
  next
}
bXse=A$bXse
```

```r
byse=A$byse
Rxy=Rnn*Ruv

fit.cismrbee2=CisMRBEEX(by=by,bX=bX,byse=byse,bXse=bXse,xQTL.max.L=5,
                        xQTL.Nvec=rep(n1,p),LD=LD2,Rxy=Rnn*Ruv,
                        reliability.thres=0.9,tauvec=seq(2,15,1),
                        ebic.gamma=2,ebic.theta=0,ridge=ridge,causal.pip.thres=pip.thres)
```

```
## Please standardize data such that BETA = Zscore/sqrt n and SE = 1/sqrt n
## Sparse prediction ends: 2.199 secs
## Causal effect estimation ends: 2.003 secs
```

```r
t2=Sys.time()

LD3=LD0[ldindex,ldindex]*0.8+diag(m)*0.2
A=MRBEEX::summary_generation(theta=theta0,m=m,Rbb=Rbb,Ruv=Ruv,Rnn=Rnn,
                             LD=LD3,Nxy=Nxy,non.zero.frac=rep(0.015,p),
                             UHP.frac=UHP.frac,CHP.frac=0,UHP.var=UHP.var,
                             Hxy=Hxy,UHP.dis="normal")
bX=A$bX
by=A$by
if(sum(is.na(by))>0){
  next
}
bXse=A$bXse
byse=A$byse
Rxy=Rnn*Ruv

fit.cismrbee3=CisMRBEEX(by=by,bX=bX,byse=byse,bXse=bXse,xQTL.max.L=5,
                        xQTL.Nvec=rep(n1,p),LD=LD3,Rxy=Rnn*Ruv,
                        reliability.thres=0.9,tauvec=seq(2,15,1),
                        ebic.gamma=2,ebic.theta=0,ridge=ridge,causal.pip.thres=pip.thres)
```

```
## Please standardize data such that BETA = Zscore/sqrt n and SE = 1/sqrt n
## Sparse prediction ends: 3.751 secs
## Causal effect estimation ends: 2.022 secs
```

```r
t2=Sys.time()

LD4=LD0[ldindex,ldindex]*0.7+diag(m)*0.3
A=MRBEEX::summary_generation(theta=theta0,m=m,Rbb=Rbb,Ruv=Ruv,Rnn=Rnn,
                             LD=LD4,Nxy=Nxy,non.zero.frac=rep(0.015,p),
                             UHP.frac=UHP.frac,CHP.frac=0,UHP.var=UHP.var,
                             Hxy=Hxy,UHP.dis="normal")
bX=A$bX
by=A$by
if(sum(is.na(by))>0){
  next
}
bXse=A$bXse
byse=A$byse
Rxy=Rnn*Ruv

fit.cismrbee4=CisMRBEEX(by=by,bX=bX,byse=byse,bXse=bXse,xQTL.max.L=5,
                        xQTL.Nvec=rep(n1,p),LD=LD4,Rxy=Rnn*Ruv,
```

```r
                         reliability.thres=0.9,tauvec=seq(2,15,1),
                         ebic.gamma=2,ebic.theta=0,ridge=ridge,causal.pip.thres=pip.thres)
```

```
## Please standardize data such that BETA = Zscore/sqrt n and SE = 1/sqrt n
## Sparse prediction ends: 0.798 secs
## Causal effect estimation ends: 1.891 secs
```

```r
t2=Sys.time()

LD5=LD0[ldindex,ldindex]*0.6+diag(m)*0.4
A=MRBEEX::summary_generation(theta=theta0,m=m,Rbb=Rbb,Ruv=Ruv,Rnn=Rnn,
                             LD=LD5,Nxy=Nxy,non.zero.frac=rep(0.015,p),
                             UHP.frac=UHP.frac,CHP.frac=0,UHP.var=UHP.var,
                             Hxy=Hxy,UHP.dis="normal")
bX=A$bX
by=A$by
if(sum(is.na(by))>0){
  next
}
bXse=A$bXse
byse=A$byse
Rxy=Rnn*Ruv

fit.cismrbee5=CisMRBEEX(by=by,bX=bX,byse=byse,bXse=bXse,xQTL.max.L=5,
                         xQTL.Nvec=rep(n1,p),LD=LD5,Rxy=Rnn*Ruv,
                         reliability.thres=0.9,tauvec=seq(2,15,1),
                         ebic.gamma=2,ebic.theta=0,ridge=ridge,causal.pip.thres=pip.thres)
```

```
## Please standardize data such that BETA = Zscore/sqrt n and SE = 1/sqrt n
## Sparse prediction ends: 1.677 secs
## Causal effect estimation ends: 1.69 secs
```

```r
t2=Sys.time()

LD6=LD0[ldindex,ldindex]*0.5+diag(m)*0.5
A=MRBEEX::summary_generation(theta=theta0,m=m,Rbb=Rbb,Ruv=Ruv,Rnn=Rnn,
                             LD=LD6,Nxy=Nxy,non.zero.frac=rep(0.015,p),
                             UHP.frac=UHP.frac,CHP.frac=0,UHP.var=UHP.var,
                             Hxy=Hxy,UHP.dis="normal")
bX=A$bX
by=A$by
if(sum(is.na(by))>0){
  next
}
bXse=A$bXse
byse=A$byse
Rxy=Rnn*Ruv

fit.cismrbee6=CisMRBEEX(by=by,bX=bX,byse=byse,bXse=bXse,xQTL.max.L=5,
                         xQTL.Nvec=rep(n1,p),LD=LD6,Rxy=Rnn*Ruv,
                         reliability.thres=0.9,tauvec=seq(2,15,1),
                         ebic.gamma=2,ebic.theta=0,ridge=ridge,causal.pip.thres=pip.thres)
```

```
## Please standardize data such that BETA = Zscore/sqrt n and SE = 1/sqrt n
## Sparse prediction ends: 0.569 secs
```

```
## Causal effect estimation ends: 1.474 secs
```

```r
t2=Sys.time()

print(cbind(fit.cismrbee1$theta,fit.cismrbee2$theta,fit.cismrbee3$theta,
            fit.cismrbee4$theta,fit.cismrbee5$theta,fit.cismrbee6$theta))
```

```
##            [,1]      [,2]      [,3]      [,4]      [,5]      [,6]
##  [1,] 1.043598 1.0742756 1.0184689 1.1512381 1.0671830 1.1147968
##  [2,] 0.000000 0.0000000 0.0000000 0.0000000 0.0000000 0.0000000
##  [3,] 0.642014 0.4986758 0.4040942 0.5192803 0.4418829 0.3910218
##  [4,] 0.000000 0.0000000 0.0000000 0.0000000 0.0000000 0.0000000
##  [5,] 0.000000 0.0000000 0.0000000 0.0000000 0.0000000 0.0000000
##  [6,] 0.000000 0.0000000 0.0000000 0.0000000 0.0000000 0.0000000
##  [7,] 0.000000 0.0000000 0.0000000 0.0000000 0.0000000 0.0000000
##  [8,] 0.000000 0.0000000 0.0000000 0.0000000 0.0000000 0.0000000
##  [9,] 0.000000 0.0000000 0.0000000 0.0000000 0.0000000 0.0000000
## [10,] 0.000000 0.0000000 0.0000000 0.0000000 0.0000000 0.0000000
```

```r
print(cbind(fit.cismrbee1$theta.se,fit.cismrbee2$theta.se,fit.cismrbee3$theta.se,
            fit.cismrbee4$theta.se,fit.cismrbee5$theta.se,fit.cismrbee6$theta.se))
```

```
##              [,1]       [,2]       [,3]       [,4]       [,5]       [,6]
##  [1,] 0.09043391 0.08568503 0.08322462 0.08053242 0.07837716 0.08201090
##  [2,] 0.00000000 0.00000000 0.00000000 0.00000000 0.00000000 0.00000000
##  [3,] 0.09257707 0.08096281 0.07828512 0.08102727 0.08020104 0.07734096
##  [4,] 0.00000000 0.00000000 0.00000000 0.00000000 0.00000000 0.00000000
##  [5,] 0.00000000 0.00000000 0.00000000 0.00000000 0.00000000 0.00000000
##  [6,] 0.00000000 0.00000000 0.00000000 0.00000000 0.00000000 0.00000000
##  [7,] 0.00000000 0.00000000 0.00000000 0.00000000 0.00000000 0.00000000
##  [8,] 0.00000000 0.00000000 0.00000000 0.00000000 0.00000000 0.00000000
##  [9,] 0.00000000 0.00000000 0.00000000 0.00000000 0.00000000 0.00000000
## [10,] 0.00000000 0.00000000 0.00000000 0.00000000 0.00000000 0.00000000
```

```r
print(cbind(fit.cismrbee1$theta.pip,fit.cismrbee2$theta.pip,fit.cismrbee3$theta.pip,
            fit.cismrbee4$theta.pip,fit.cismrbee5$theta.pip,fit.cismrbee6$theta.pip))
```

```
##               [,1]         [,2]         [,3]         [,4]         [,5]
##  [1,] 1.000000e+00 1.000000e+00 1.000000e+00 1.000000e+00 1.000000e+00
##  [2,] 3.330669e-15 5.324726e-10 1.815107e-06 8.884343e-10 4.781192e-07
##  [3,] 1.000000e+00 1.000000e+00 9.995951e-01 9.999999e-01 9.999971e-01
##  [4,] 3.141931e-14 1.146205e-09 1.984997e-06 6.496448e-10 2.446098e-07
##  [5,] 1.432188e-13 7.647774e-10 3.863765e-04 1.062721e-09 2.527178e-07
##  [6,] 3.885781e-15 5.376578e-10 1.896820e-06 1.353479e-09 3.817994e-07
##  [7,] 2.668288e-11 8.751821e-10 2.174114e-06 6.122599e-10 2.531580e-07
##  [8,] 4.329870e-15 1.517000e-09 3.242546e-06 1.185645e-09 3.085543e-07
##  [9,] 2.997602e-15 1.187021e-09 3.831697e-06 5.764904e-08 3.561409e-07
## [10,] 1.110223e-14 5.415373e-10 1.637805e-06 1.125790e-09 3.334636e-07
##               [,6]
##  [1,] 1.000000e+00
##  [2,] 3.112250e-06
##  [3,] 9.999561e-01
##  [4,] 5.882475e-06
##  [5,] 2.889752e-06
##  [6,] 3.039936e-06
##  [7,] 2.891311e-06
```

```
##  [8,] 5.941737e-06
##  [9,] 2.960786e-06
## [10,] 1.416154e-05
```

2.3 Simulations of univariable cis-Mendelian randomization

Finally, we evaluate whether cis-MRBEE outperforms existing methods in cis-UVMR. We first generate the data using the same settings as examination of discrete differential penalty.

```
library(MRAID)
library(cisMRcML)
library(MESuSiE)
m=200
p=10
n1=30000
pip.thres=0.3
ridge=0
n0=0.5e6
Rbb=ARcov(p,-0.5)
Ruv=ARcov(p+1,-0.3)
Nxy=c(rep(n1,p),n0)
Hxy=c(rep(.3,p),.001)
Rnn=matrix(1,p+1,p+1)
theta0=c(1,0,rep(0,p-2))
UHP.var=1
UHP.frac=0.00
xQTL.pip.thres=0.5
LD0=readRDS("RDS/LD.rds")%>%do.call(Matrix::bdiag,.)%>%as.matrix(.)
ldindex=sample(floor(2083/m),1,replace=F)
ldindex=c(((ldindex-1)*m+1):(ldindex*m))
LD=LD0[ldindex,ldindex]*0.75+0.25*diag(m)
A=MRBEEX::summary_generation(theta=theta0,m=m,Rbb=Rbb,Ruv=Ruv,Rnn=Rnn,LD=LD,
                             Nxy=Nxy,non.zero.frac=rep(0.015,p),UHP.frac=UHP.frac,
                             CHP.frac=0,UHP.var=UHP.var,Hxy=Hxy,UHP.dis="normal")
bX=A$bX[,1]
by=A$by
if(sum(is.na(by))>0){
next
}
bXse=A$bXse[,1]
byse=A$byse
Rxy=Rnn*Ruv
Rxy=Rxy[c(1,11),c(1,11)]
```

We compare cis-MRBEE with cis-IVW, PC-GMM, MRAID (Yuan et al., 2022), cis-MRcML-DP, and cis-MRcML-BIC (Lin and Pan, 2024). Among these, MRAID is conceptually similar to cis-MRBEE: MRAID employs a slab-and-spike-like prior distribution for xQTL effects and horizontal pleiotropy and estimates these effects along with the causal effect within a single model. In contrast, cis-MRBEE uses SuSiE to estimate xQTL effects first, followed by MRBEE-IPOD to estimate the causal effect and horizontal pleiotropy. (Since the scale of causal effect estimate of MRAID is based on the variance of exposure and outcome are all 1, we re-scale the causal effect estimate of MRAID.)

```
MVINPUT=mr_input(bx=bX,bxse=bXse,by=by,byse=byse,correlation=LD)
t1=Sys.time()
fit.ivw=mr_ivw(MVINPUT,correl=T)
```

```
t2=Sys.time()
ivw.time=difftime(t2, t1, units = "secs")

t1=Sys.time()
fit.pca=mr_pcgmm(MVINPUT,ny=n0,nx=n1,thres=pca.thres)
t2=Sys.time()
pca.time=difftime(t2, t1, units = "secs")

t1=Sys.time()
fit.susie=CisMRBEE_UV(by,bX,byse,bXse,LD=LD,Rxy=Rxy,xQTL.N=n1,xQTL.max.L=5,
                      xQTL.pip.thres=xQTL.pip.thres,reliability.thres=0.8)
t2=Sys.time()
susie.time=difftime(t2, t1, units = "secs")

t1=Sys.time()
fit.aid=MRAID(Zscore_1=bX/bXse,Zscore_2=by/byse,Sigma1sin=LD,Sigma2sin=LD,
              samplen1=n1,samplen2=n0,Gibbsnumber=5000,burninproportion=0.1)
fit.aid$causal_effect=fit.aid$causal_effect*sqrt(A$Syy)
t2=Sys.time()
aid.time=difftime(t2, t1, units = "secs")
```

On the other hand, the cis-MRcML methods require pre-selecting IVs that are informative for exposures, outcomes, or both before performing causal effect estimation. The authors of cis-MRcML recommend using COJO (Yang et al., 2012) for IV selection. In this study, we instead employ MESuSIE for IV selection. (Sometimes, cis-MRcML will return null estimate, which we set as 0 in simulation.)

```
AtLeastPIP=function(A){
  g=apply(1-A,1,prod)
  g=1-g
  return(g)
}
colnames(LD)=rownames(LD)=paste0("SNP",c(1:m))
X=data.frame(SNP=colnames(LD),Beta=bX,Se=bXse,Z=bX/bXse,N=n1);
Y=data.frame(SNP=colnames(LD),Beta=by,Se=byse,Z=by/byse,N=n0);
rownames(X)=X$SNP;rownames(Y)=Y$SNP
fit.mesusie=meSuSie_core(R_mat_list=list(X=LD,Y=LD),summary_stat_list=list(X=X,Y=Y),L=10)
```

```
## ****************************************************************
##
##   Multiple Ancestry Sum of Single Effect Model (MESuSiE)
##
##    Visit http://www.xzlab.org/software.html For Update
##
##              (C) 2022 Boran Gao, Xiang Zhou
##
##                GNU General Public License
##
## ****************************************************************
## # Start data processing for sufficient statistics
## # Create MESuSiE object
## # Start data analysis
##
## # Data analysis is done, and now generates result
##
```

```
## Potential causal SNPs with PIP > 0.5:  SNP14 SNP157 SNP171
##
## Credible sets for effects:
## $cs
## $cs$L1
## [1] 157
##
## $cs$L2
## [1] 171
##
## $cs$L3
## [1] 14
##
##
## $cs_category
##    L1    L2    L3
## "X_Y" "X_Y" "X_Y"
##
## $purity
##    min.abs.corr mean.abs.corr median.abs.corr
## L1            1             1               1
## L2            1             1               1
## L3            1             1               1
##
## $cs_index
## [1] 1 2 3
##
## $coverage
## [1] 1 1 1
##
## $requested_coverage
## [1] 0.95
##
##
##  Use MESuSiE_Plot() for visualization
## # Total time used for the analysis: 0.24 mins
```

```
SNPSelect=X$SNP[which(AtLeastPIP(fit.mesusie$pip_config)>0.3)]
if(length(SNPSelect)<=3){
SNPSelect=c(SNPSelect,sample(setdiff(colnames(LD),SNPSelect),3-length(SNPSelect)))
}
## Guarantee there is enough number of IVs
bXSelect=as.vector(solve(LD[SNPSelect,SNPSelect])%*%(X[SNPSelect,"Beta"]/X[SNPSelect,"Se"]))*X[SNPSelec
bXcovSelect=t(LD[SNPSelect,SNPSelect]*X[SNPSelect,"Se"])*X[SNPSelect,"Se"]
bXcovSelect=solve(LD[SNPSelect,SNPSelect])%*%bXcovSelect%*%solve(LD[SNPSelect,SNPSelect])
bySelect=as.vector(solve(LD[SNPSelect,SNPSelect])%*%(Y[SNPSelect,"Beta"]/Y[SNPSelect,"Se"]))*Y[SNPSelec
bycovSelect=t(LD[SNPSelect,SNPSelect]*Y[SNPSelect,"Se"])*Y[SNPSelect,"Se"]
bycovSelect=solve(LD[SNPSelect,SNPSelect])%*%bycovSelect%*%solve(LD[SNPSelect,SNPSelect])
fit.cisMRcML=cismr_cML_DP(b_exp=bXSelect,b_out=bySelect,Sig_exp_inv=bXcovSelect,
                          Sig_out_inv=bycovSelect,n=n1)
```

```
## Warning in min(BIC_vec, na.rm = T): no non-missing arguments to min; returning
## Inf
## Warning in min(BIC_vec, na.rm = T): no non-missing arguments to min; returning
## Inf
```

```
## Warning in min(BIC_vec, na.rm = T): no non-missing arguments to min; returning
## Inf
```

```
if(length(fit.cisMRcML$BIC_se)==0){
fit.cisMRcML$BIC_se=fit.cisMRcML$BIC_theta=0
}
print(cbind(c(fit.ivw@Estimate,fit.pca@Estimate,fit.aid$causal_effect,
              fit.cisMRcML$BIC_theta,fit.cisMRcML$BIC_DP_theta,fit.susie$theta),
            c(fit.ivw@StdError,fit.pca@StdError,
              fit.aid$causal_effect/sqrt(qchisq(fit.aid$causal_pvalue,1,lower.tail=F)),
              fit.cisMRcML$BIC_se,fit.cisMRcML$BIC_DP_se,fit.susie$theta.se)))
```

```
##            [,1]         [,2]
## [1,] 0.89493622   0.05727175
## [2,] 0.90143804   0.05885195
## [3,] 0.31128429   0.16914063
## [4,] 0.89663462 305.19325199
## [5,] 0.03746567   0.25255231
## [6,] 0.90049446   0.07483208
```

We also investigate the type-I error, where we set:

```
bX=A$bX[,10]
by=A$by
if(sum(is.na(by))>0){
next
}
bXse=A$bXse[,10]
byse=A$byse
Rxy=Rnn*Ruv
Rxy=Rxy[c(10,p+1),c(10,p+1)]
```

3 Supplementary Statistical Method

3.1 Multivariable cis-Mendelian randomization model

Recall the we define the following two statistical model in (1) and (2) in the manuscript:

$$y_i = \sum_{j=1}^{p} x_{ij}\theta_j + \sum_{m=1}^{M} g_{im}\gamma_m + \epsilon_{i0}, \tag{1}$$

$$x_{ij} = \sum_{s=1}^{m} g_{is}\beta_{sj} + \epsilon_{ij}, \quad 1 \le j \le p, \tag{2}$$

where y_i is the outcome, $x_{i1}, \dots, x_{ip}$ are p number of exposures, $g_{i1}, \dots, g_{im}$ are m number of IVs, $\beta_{1j}, \dots, \beta_{mj}$ are the corresponding genetic effects for the jth exposures, $\gamma_1, \dots, \gamma_m$ are the horizontal pleiotropy (also known as direct genetic effect) of the outcome, $\theta_1, \dots, \theta_p$ are the causal effects of the exposures, and $\epsilon_{i0}, \epsilon_{i1}, \dots, \epsilon_{ip}$ are the white noises. It is assumed that the IVs $g_{i1}, \dots, g_{im}$ are mutually independent and independent of the error terms $\epsilon_{i0}, \epsilon_{i1}, \dots, \epsilon_{ip}$. Additionally, in cis-MVMR, the exposures are usually some gene products (e.g., gene expressions and protein abundance), and the IVs are the corresponding cis-regulatory QTL (cis-xQTLs) of these gene products.

The principles of the traditional cis-MVMR method can be summarized as follows, although it may differ slightly from this description in practice. First, cis-MVMR estimates the genetic effects $\boldsymbol{\beta}_j = (\beta_{1j}, \dots, \beta_{mj})^\top$

using ordinary least square (OLS):

$$\hat{\boldsymbol{\beta}}_j^{\text{OLS}} = \arg\min_{\boldsymbol{\beta}_j \in \mathbb{R}^m} \left\{ \frac{1}{n} \sum_{i=1}^{n} (x_{ij} - \boldsymbol{g}_j^\top \boldsymbol{\beta}_j)^2 \right\}, \quad 1 \leq j \leq p, \tag{3}$$

where $\boldsymbol{g}_i = (g_{i1}, \ldots, g_{ip})^\top$. While GWAS of different exposures may be conducted in different samples in practice, for simplicity, we assume here that both the exposures and the outcome come from the same sample. Second, cis-MVMR predicts the exposures using the estimated effects: $\hat{x}_{ij}^{\text{OLS}} = \boldsymbol{g}_i^\top \hat{\boldsymbol{\beta}}_j^{\text{OLS}}$. Third, cis-MVMR estimate the causal effect vector $\boldsymbol{\theta} = (\theta_1, \ldots, \theta_p)^\top$ using the predicted exposures $\hat{\boldsymbol{x}}_i^{\text{OLS}} = (\hat{x}_{i1}^{\text{OLS}}, \ldots, \hat{x}_{ip}^{\text{OLS}})$ using OLS:

$$\hat{\boldsymbol{\theta}} = \arg\min_{\boldsymbol{\theta} \in \mathbb{R}^p} \left\{ \frac{1}{n} \sum_{i=1}^{n} (y_i - \hat{\boldsymbol{x}}_i^{\text{OLS}\top} \boldsymbol{\theta})^2 \right\}. \tag{4}$$

This sort of procedure is known as a two-stage least square (2SLS). Nevertheless, Lorincz-Comi et al. (2024) pointed out that the predicted exposure $\hat{\boldsymbol{x}}_{ij}^{\text{OLS}}$ includes measurement error $\boldsymbol{w}_{ij}^{\text{OLS}} = \boldsymbol{g}_i^\top (\hat{\boldsymbol{\beta}}_j^{\text{OLS}} - \boldsymbol{\beta}_j)$. As a result, the causal effect estimate $\hat{\boldsymbol{\theta}}$ is subject to estimation error bias according to the theory of measurement error analysis, even though the horizontal pleiotropy $\boldsymbol{\gamma} = (\gamma_1, \ldots, \gamma_p)^\top$ does not exist. In our opinion, estimation errors are the primary source of weak instrument bias, where the strength of the instrument varies dynamically, depending on the relative size of the true genetic effects and the estimation errors.

Motivated by this, Lorincz-Comi et al. (2024) proposed MRBEE that leverages the method of unbiased estimating function to remove the measurement error bias. Specifically, MV-IVW (Bowden et al., 2016), a fundamental method in MVMR, estimates $\boldsymbol{\theta}$ by:

$$\hat{\boldsymbol{\theta}}_{\text{IVW}} = \arg\min_{\boldsymbol{\theta}} \left\{ \frac{1}{2} \|\hat{\boldsymbol{\alpha}} - \hat{\mathbf{B}}\boldsymbol{\theta}\|_2^2 \right\}, \tag{5}$$

and equivalently, $\hat{\boldsymbol{\theta}}_{\text{IVW}}$ is yielded such that $\mathrm{U}_{\text{IVW}}(\hat{\boldsymbol{\theta}}_{\text{IVW}}) = 0$, where $\mathrm{U}_{\text{IVW}}(\boldsymbol{\theta}) = -\hat{\mathbf{B}}^\top(\hat{\boldsymbol{\alpha}} - \hat{\mathbf{B}}\boldsymbol{\theta})$ is the gradient function of the above minimization, $\hat{\mathbf{B}} = (\hat{\boldsymbol{b}}_1, \ldots, \hat{\boldsymbol{b}}_p)$, and $\mathbf{R}$ is required to be the identity matrix. Since $\hat{\mathbf{B}}$ are estimated and contains estimation error, $\hat{\boldsymbol{\theta}}_{\text{IVW}}$ is subject to the measurement error bias (Yi, 2016). In other words, (1) weak instrument bias originates from estimation errors in the GWAS effect estimates of exposures, and (2) the severity of this bias is influenced by the relative size of the true genetic effect compared to the estimation error (Lorincz-Comi et al., 2024). MRBEE estimates $\boldsymbol{\theta}$ by using the following bias-corrected estimating equation:

$$\mathrm{U}_{\text{BEE}}(\boldsymbol{\theta}) = \mathrm{U}_{\text{IVW}}(\hat{\boldsymbol{\theta}}_{\text{IVW}}) - \mathrm{E}(\mathrm{U}_{\text{IVW}}(\hat{\boldsymbol{\theta}}_{\text{IVW}})), \tag{6}$$

$$\mathrm{E}(\mathrm{U}_{\text{IVW}}(\hat{\boldsymbol{\theta}}_{\text{IVW}})) = \mathrm{E}\bigg((\hat{\mathbf{B}} - \mathbf{B})^\top (\hat{\mathbf{B}} - \mathbf{B})\boldsymbol{\theta} - (\hat{\mathbf{B}} - \mathbf{B})^\top (\hat{\boldsymbol{\alpha}} - \boldsymbol{\alpha}) \bigg), \tag{7}$$

which is essentially driven by the estimation errors of GWAS estimates and can be estimated by using statistically insignificant statistics(Lorincz-Comi et al., 2024).

However, extending MRBEE to the cis-MVMR setting posed challenges because MRBEE and other existing cis-MVMR methods overlook an important genetic architecture: in a cis-region, only a few causal variants exist, while most significantly associated variants are due to LD with causal variants. Statistically, this means that $\boldsymbol{\beta}_j$ is a sparse vector and $\text{cov}(\boldsymbol{g}_i) = \mathbf{R}$ is a correlation matrix with non-zero off-diagonal elements. In this case, IVs are often extremely weak, as most have true effects of zero. Methods in measurement error analysis generally perform poorly when the relative scale of measurement errors is large compared to the true effects (Yi, 2016). Consequently, directly extending MRBEE to cis-MVMR often identifies outcome-associated causal variants as horizontal pleiotropy while neglecting exposures.

3.2 Sparse prediction of GWAS effect

Cis-MRBEE is purely a summarized-statistics-based MVMR method, which relies on the counterpart of the equations (1) and (2) on summarized statistics (Zhu and Stephens, 2017):

$$\hat{\boldsymbol{a}} \sim \mathcal{N}\bigg(\mathbf{R}(\sum_{j=1}^{p}\beta_j\theta_j + \boldsymbol{\gamma}), \sigma_\alpha^2\mathbf{R}\bigg), \tag{8}$$

$$\hat{\boldsymbol{b}}_j \sim \mathcal{N}\bigg(\mathbf{R}\boldsymbol{\beta}_j, \sigma_{\beta_j}^2\mathbf{R}\bigg), \tag{9}$$

where $\hat{\boldsymbol{a}} = (\hat{a}_1, \dots, \hat{a}_m)^\top$ is the effect size vector of outcome GWAS, $\hat{\boldsymbol{b}}_j = (\hat{b}_{1j}, \dots, \hat{b}_{mj})^\top$ is the effect size vector of the jth exposure GWAS, with $\hat{a}_s = n_0^{-1}\sum_{i=1}^{n_0} y_i^{[0]} g_{is}^{[0]}$, $\hat{b}_{sj} = n_j^{-1}\sum_{i=1}^{n_j} x_{ij}^{[j]} g_{is}^{[j]}$, $y_i^{[0]}$, $x_{ij}^{[j]}$, and $g_{is}^{[j]}$ mean that they come from the GWAS cohort of the jth exposures and n_j is the related sample size, $0 \leq j \leq p$.

Cis-MRBEE proposes to first estimate $\boldsymbol{\beta}_j$ by a penalized least square (PLS):

$$\hat{\boldsymbol{\beta}}_j^{\text{PLS}} = \arg\min_{\boldsymbol{\beta}_j \in \mathbb{R}^m} \left\{\frac{1}{2}\boldsymbol{\beta}_j^\top\mathbf{R}\boldsymbol{\beta}_j - \hat{\boldsymbol{b}}_j^\top\boldsymbol{\beta}_j + p_{\boldsymbol{\lambda}}(\boldsymbol{\beta}_j)\right\}, \tag{10}$$

where $p_{\boldsymbol{\lambda}}(\cdot)$ is a penalty function with a vector of involved parameter $\boldsymbol{\lambda}$. From the Bayesian point of view, $p_{\boldsymbol{\lambda}}(\boldsymbol{\beta}_j) \propto -\log f_{\boldsymbol{\lambda}}(\boldsymbol{\beta}_j)$ where $f_{\boldsymbol{\lambda}}(\boldsymbol{\beta}_j)$ is the density function of the prior distribution of $\boldsymbol{\beta}_j$.

In cis-MRBEE, SuSiE is used to implement (10) which is the most popular fine-mapping method for the summarized-statistic-based model. Specifically, as described in Wang et al. (2020), SuSiE considers the following hierarchical Bayesian model:

$$\begin{aligned} y &= \boldsymbol{X}\boldsymbol{\beta} + \boldsymbol{e}, \\ \boldsymbol{e} &\sim \mathcal{N}(0, \sigma^2 I_n), \\ \boldsymbol{\beta} &= \sum_{l=1}^{L}\mathbf{b}_l, \\ \mathbf{b}_l &\sim \gamma_l b_l, \\ \gamma_l &\sim \text{Mult}(1, \pi), \\ b_l &\sim \mathcal{N}_p(0, \sigma^2\sigma_{0l}^2), \end{aligned} \tag{11}$$

It is challenging to explicitly specify the prior for the parameter $\boldsymbol{\beta}$. However, Wang et al. (2020) demonstrate in their supplementary materials that, for a fixed L, as the number of variables $p \to \infty$, SuSiE is equivalent to Bayesian variable selection regression (BVSR) based on a spiked-and-slab prior.

Causal variants are calibrated based on the PIPs estimated by SuSiE. In addition, the covariance matrix $\hat{\boldsymbol{\beta}}_j^{\text{PLS}}$ is constructed according to oracle property. Let $\mathcal{M}_j = \{s,\ \hat{\beta}_{js}^{\text{PLS}} \neq 0\}$ and $\boldsymbol{\Theta} = \mathbf{R}^{-1}$ is the inverse of the LD matrix. Then

$$\sqrt{n_j}(\hat{\boldsymbol{\beta}}_{j\mathcal{M}_j}^{\text{PLS}} - \boldsymbol{\beta}_{j\mathcal{M}_j}) \sim \mathcal{N}(\mathbf{0}, \boldsymbol{\Theta}_{\mathcal{M}_j\mathcal{M}_j}), \tag{12}$$

where $\hat{\boldsymbol{\beta}}_{j\mathcal{M}j}^{\text{PLS}}$ and $\boldsymbol{\beta}_{j\mathcal{M}j}$ are sub-vectors of $\hat{\boldsymbol{\beta}}_j$ and $\boldsymbol{\beta}_j$, indexed by $\mathcal{M}_j$, and $\boldsymbol{\Theta}_{\mathcal{M}_j\mathcal{M}_j} = \mathbf{R}_{\mathcal{M}_j\mathcal{M}_j}^{-1}$ ($\mathbf{R}_{\mathcal{M}_j\mathcal{M}_j}^{-1}$ is the sub-matrix of $\mathbf{R}$ corresponding to the rows and columns in $\mathcal{M}_j$). It is important to note that SuSiE may identify multiple variants within a single credible set. In such cases, we adopt the "top_K" principle, selecting the top K variants with the highest PIP values within the credible set as informative variants for sparse prediction, where the default value of K is 1. Additionally, if SuSiE does not identify any credible set, we select individual variants with PIP greater than a predefined threshold (xQTL.pip.thres) as informative variants.

Based on (12), the covariance matrix of $\hat{\boldsymbol{b}}_j^{\text{PLS}} = \mathbf{R}\hat{\boldsymbol{\beta}}_j^{\text{PLS}}$ is derived as follows. First, under the theoretical assumption that $\hat{\boldsymbol{\beta}}_j^{\text{PLS}}$ is re-ordered as $\hat{\boldsymbol{\beta}}_j^{\text{PLS}} = (\hat{\boldsymbol{\beta}}_{j\mathcal{M}j}^{\text{PLS}^\top}, \mathbf{0})^\top$, we can write $\mathbf{R} = (\mathbf{R}_{\mathcal{M}j}, \mathbf{R}_{\mathcal{M}_j^c})$, where $\mathbf{R}_{\mathcal{M}_j}$ is

a $p \times |\mathcal{M}_j|$ submatrix corresponding to the non-zero elements in $\hat{\boldsymbol{\beta}}_{j\mathcal{M}_j}$, and $\mathcal{M}_j^c$ is the complement of $\mathcal{M}_j$. The covariance matrix of $\hat{\boldsymbol{b}}_j^{\mathrm{PLS}}$ is then given by:

$$\mathrm{cov}(\hat{\boldsymbol{b}}_j^{\mathrm{PLS}}) = \frac{1}{n_j}\mathbf{R}\begin{pmatrix} \boldsymbol{\Theta}_{\mathcal{M}_j\mathcal{M}_j} & \mathbf{0} \\ \mathbf{0} & \mathbf{0} \end{pmatrix}\mathbf{R} = \frac{1}{n_j}\begin{pmatrix} \mathbf{R}_{\mathcal{M}_j\mathcal{M}_j} & \mathbf{0} \\ \mathbf{0} & \mathbf{0} \end{pmatrix} \tag{13}$$

Then $\mathrm{se}(\hat{\boldsymbol{b}}_j^{\mathrm{PLS}})$ is the diagonal elements of the above matrix.

Based on the asymptotic distribution of $\hat{\boldsymbol{\beta}}_{j\mathcal{M}_j}^{\mathrm{PLS}}$, we can obtain

$$\mathrm{E}(\|\hat{\boldsymbol{\beta}}_{j\mathcal{M}_j}^{\mathrm{PLS}} - \boldsymbol{\beta}_{j\mathcal{M}_j}\|_2^2) = \frac{\mathrm{tr}(\boldsymbol{\Theta}_{\mathcal{M}_j\mathcal{M}_j})}{n_j} \asymp O(\frac{|\mathcal{M}_j|}{n_j}), \tag{14}$$

where $|\mathcal{M}_j|$ is the size of the set $\mathcal{M}_j$. Note that the entries outside the set $\mathcal{M}_j$ is zero. As for $\hat{\boldsymbol{\beta}}^{\mathrm{OLS}}$, which is defined as $\mathbf{R}^{-1}\hat{\boldsymbol{b}}$,

$$\sqrt{n_j}(\hat{\boldsymbol{\beta}}_j^{\mathrm{OLS}} - \boldsymbol{\beta}_j) \sim \mathcal{N}(\mathbf{0}, \mathbf{R}^{-1}), \tag{15}$$

and hence

$$\mathrm{E}(\|\hat{\boldsymbol{\beta}}_j^{\mathrm{OLS}} - \boldsymbol{\beta}_j\|_2^2) = \frac{\mathrm{tr}(\mathbf{R}^{-1})}{n_j} \asymp O(\frac{m}{n_j}). \tag{16}$$

In practice, $m >> |\mathcal{M}_j|$ and hence $\hat{\boldsymbol{\beta}}_j^{\mathrm{PLS}}$ is more accurate that $\hat{\boldsymbol{\beta}}_j^{\mathrm{OLS}}$ in terms of mean square error (MSE). As for prediction error (PE), we have

$$\begin{aligned}\mathrm{E}(\|\mathbf{R}(\hat{\boldsymbol{\beta}}_j^{\mathrm{PLS}} - \boldsymbol{\beta}_j)\|_2^2) &= \mathrm{tr}(\mathrm{cov}(\mathbf{R}(\hat{\boldsymbol{\beta}}_j^{\mathrm{PLS}}))) = \mathrm{tr}(\frac{1}{n_j}\mathbf{R}\begin{pmatrix} \boldsymbol{\Theta}_{\mathcal{M}_j\mathcal{M}_j} & \mathbf{0} \\ \mathbf{0} & \mathbf{0} \end{pmatrix}\mathbf{R}) = \mathrm{tr}(\frac{1}{n_j}\begin{pmatrix} \mathbf{R}_{\mathcal{M}_j\mathcal{M}_j} & \mathbf{0} \\ \mathbf{0} & \mathbf{0} \end{pmatrix}) \\ &= \frac{|\mathcal{M}_j|}{n_j},\end{aligned} \tag{17}$$

and

$$\mathrm{E}(\|\mathbf{R}(\hat{\boldsymbol{\beta}}_j^{\mathrm{OLS}} - \boldsymbol{\beta}_j)\|_2^2) = \mathrm{E}(\|\hat{\boldsymbol{b}}_j - \mathbf{R}\boldsymbol{\beta}_j\|_2^2) = \mathrm{tr}(\mathrm{cov}(\mathbf{R}(\hat{\boldsymbol{\beta}}_j^{\mathrm{OLS}}))) = \mathrm{tr}(\frac{1}{n_j}\mathbf{R}) = \frac{m}{n_j}. \tag{18}$$

Hence, in terms of PE, $\hat{\boldsymbol{\beta}}_j^{\mathrm{PLS}}$ is still more accurate that $\hat{\boldsymbol{\beta}}_j^{\mathrm{OLS}}$.

To validate our theoretical derivation, we designed a simulation study. We considered four scenarios for heritability (i.e., $\|\boldsymbol{\beta}_j\|_2^2$), specifically 0.05, 0.1, 0.2, and 0.3, and three different sample sizes (i.e., n_j) of 300, 3000, and 30,000. The size of $\mathcal{M}_j$ was fixed at 3, and for each simulation, we randomly selected $\mathcal{M}_j$, while keeping $\mathbf{R}$ consistent with the simulation setup in the main text. We used SuSiE as the variable selection method to identify $\mathcal{M}_j$ and estimate the corresponding $\hat{\boldsymbol{\beta}}_j^{\mathrm{PLS}}$. We conducted 500 simulations and visualized the log10-transformed Prediction Error (PE) and Mean Squared Error (MSE). The results clearly show that SuSiE consistently outperforms OLS in terms of PE and MSE across all scenarios. Although OLS improves with increasing sample size, particularly in terms of Prediction Errors, it still lags behind SuSiE in all cases.

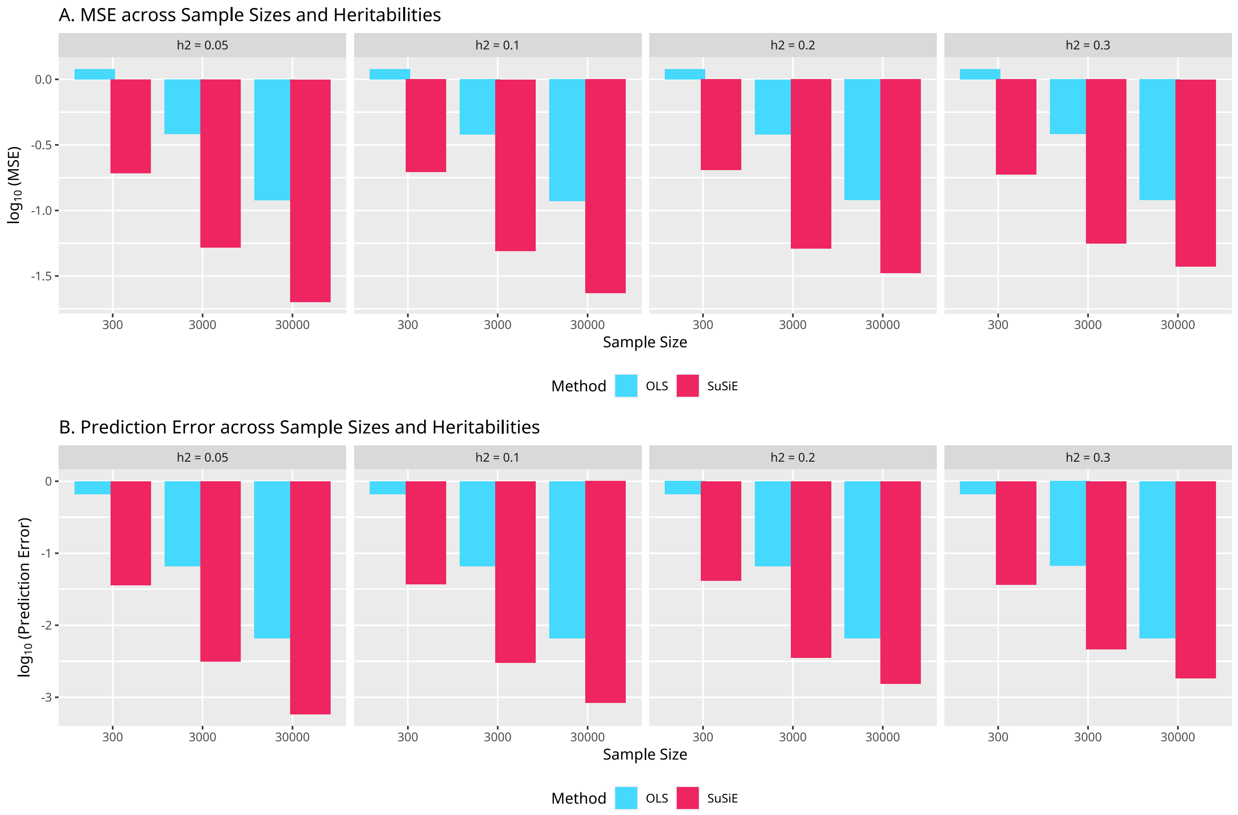

This figure shows the barplots of MSEs and PEs yielded by OLS estimate and PLE estimate, respectively. The Estimation Error in Equantion (13) showed in the main text is proportional to the PE.

3.3 Estimation of causal effect and horizontal pleiotropy

Cis-MRBEE turns to estimate the causal effect vector $\boldsymbol{\theta}$ and the horizontal pleiotropy vector $\boldsymbol{\gamma}$ by:

$$(\hat{\boldsymbol{\theta}}^\top, \hat{\boldsymbol{\gamma}}^\top)^\top = \arg\min_{\boldsymbol{\theta}\in\mathbb{R}^p, \boldsymbol{\gamma}\in\mathbb{R}^m} \left\{ \frac{1}{2}\left(\hat{\boldsymbol{a}} - \widehat{\mathbf{B}}^{\mathrm{PLS}}\boldsymbol{\theta} - \mathbf{R}\boldsymbol{\gamma}\right)^\top \mathbf{R}^{-1}\left(\hat{\boldsymbol{a}} - \widehat{\mathbf{B}}^{\mathrm{PLS}}\boldsymbol{\theta} - \mathbf{R}\boldsymbol{\gamma}\right) + p_{\boldsymbol{\lambda}_\theta}(\boldsymbol{\theta}) + q_{\boldsymbol{\lambda}_\gamma}(\boldsymbol{\gamma}) \right\}, \quad (19)$$

where $\widehat{\mathbf{B}}^{\mathrm{PLS}} = (\mathbf{R}\hat{\beta}_1^{\mathrm{PLS}}, \dots, \mathbf{R}\hat{\beta}_p^{\mathrm{PLS}})$ is the matrix of sparsely predicted GWAS effects, $p_{\boldsymbol{\lambda}_\theta}(\cdot)$ and $q_{\boldsymbol{\lambda}_\gamma}(\cdot)$ are two penalty functions with parameters $\boldsymbol{\lambda}_\theta$ and $\boldsymbol{\lambda}_\gamma$. Cis-MRBEE utilizes the profile-likelihood that updates $\boldsymbol{\theta}^{(t+1)}$ by:

$$\boldsymbol{\theta}^{(t+1)} = \arg\min_{\boldsymbol{\theta}\in\mathbb{R}^p} \left\{ \frac{1}{2}\left(\hat{\boldsymbol{a}} - \widehat{\mathbf{B}}^{\mathrm{PLS}}\boldsymbol{\theta} - \mathbf{R}\boldsymbol{\gamma}^{(t)}\right)^\top \mathbf{R}^{-1}\left(\hat{\boldsymbol{a}} - \widehat{\mathbf{B}}^{\mathrm{PLS}}\boldsymbol{\theta} - \mathbf{R}\boldsymbol{\gamma}^{(t)}\right) + p_{\boldsymbol{\lambda}_\theta}(\boldsymbol{\theta}) \right\}, \quad (20)$$

and then updates $\boldsymbol{\gamma}^{(t+1)}$ by:

$$\boldsymbol{\gamma}^{(t+1)} = \arg\min_{\boldsymbol{\gamma}\in\mathbb{R}^m} \left\{ \frac{1}{2}\left(\hat{\boldsymbol{a}} - \widehat{\mathbf{B}}^{\mathrm{PLS}}\boldsymbol{\theta}^{(t+1)} - \mathbf{R}\boldsymbol{\gamma}\right)^\top \mathbf{R}^{-1}\left(\hat{\boldsymbol{a}} - \widehat{\mathbf{B}}^{\mathrm{PLS}}\boldsymbol{\theta}^{(t+1)} - \mathbf{R}\boldsymbol{\gamma}\right) + q_{\boldsymbol{\lambda}_\gamma}(\boldsymbol{\gamma}) \right\}. \quad (21)$$

In the literature, this procedure is known as the IPOD algorithm (She and Owen, 2011).

We use SuSiE (susie_suff_stat(·)) to yield $\boldsymbol{\theta}^{(t+1)}$. Specifically, susie_suff_stat(·) requires the users to input three key statistics: XtX, Xty, yty, which are given by:

$$\mathrm{XtX} = \widehat{\mathbf{B}}^{\mathrm{PLS}\top}\mathbf{R}^{-1}\widehat{\mathbf{B}}^{\mathrm{PLS}}, \ \mathrm{Xty} = \widehat{\mathbf{B}}^{\mathrm{PLS}\top}\mathbf{R}^{-1}(\hat{\boldsymbol{a}} - \mathbf{R}\boldsymbol{\gamma}^{(t)}), \ \mathrm{yty} = (\hat{\boldsymbol{a}} - \mathbf{R}\boldsymbol{\gamma}^{(t)})^\top\mathbf{R}^{-1}(\hat{\boldsymbol{a}} - \mathbf{R}\boldsymbol{\gamma}^{(t)}). \quad (22)$$

As for L, we consider $L = 1, \ldots, L_{\max}$ and select the optimal one using EBIC. (In other words, λ_θ is just L in the update of $\boldsymbol{\theta}^{(t+1)}$.) Furthermore, susie_suff_stat(·) also requires to give the "sample size" n, where we use the number of IVs.

On the other hand, we employ the alternating direction method of multipliers (ADMM, Boyd et al. (2011)) to minimize (21). Specifically, we use the minimax concave penalty (MCP, Zhang (2010)) as $q_{\boldsymbol{\lambda}_\gamma}$, which is expressed as:

$$q_{\tau,a}^{\mathrm{MCP}}(x) = \tau \int_0^{|x|} \left(1 - \frac{|x|}{a\tau}\right)_+ dx \tag{23}$$

where $(x)+ = \max(x, 0)$ and $a > 1$ is an alternative tuning parameter that controls the concavity of $q_{\tau,a}^{\mathrm{MCP}}(\cdot)$. When we denote $q_{\tau,a}^{\mathrm{MCP}}(\boldsymbol{x})$ for a multivariate vector $\boldsymbol{x} = (x_1, \ldots, x_p)^\top$, it refers to

$$q_{\tau,a}^{\mathrm{MCP}}(\boldsymbol{x}) = \sum_{j=1}^{p} q_{\tau,a}^{\mathrm{MCP}}(x_j). \tag{24}$$

In this paper, we fix $a = 3$, as suggested by the original author, making τ the sole parameter in MCP. Second, the ADMM framework introduces an additional parameter, $\boldsymbol{\gamma}_1$, into (21), transforming it into

$$\begin{aligned} \boldsymbol{\gamma}^{(t+1)} = \arg\min_{\boldsymbol{\gamma}\in\mathbb{R}^m} &\left\{ \frac{1}{2}\left(\hat{\boldsymbol{a}} - \widehat{\mathbf{B}}^{\mathrm{PLS}}\boldsymbol{\theta}^{(t+1)} - \mathbf{R}\boldsymbol{\gamma}\right)^\top \mathbf{R}^{-1}\left(\hat{\boldsymbol{a}} - \widehat{\mathbf{B}}^{\mathrm{PLS}}\boldsymbol{\theta}^{(t+1)} - \mathbf{R}\boldsymbol{\gamma}\right) + q_\tau(\boldsymbol{\gamma}_1)\right\}, \\ &\text{subject to } \boldsymbol{\gamma} = \boldsymbol{\gamma}_1. \end{aligned} \tag{25}$$

Then ADMM uses the augmented Lagrangian method to handle it, which results in the following Q-function:

$$\begin{aligned} Q(\boldsymbol{\gamma}, \boldsymbol{\gamma}_1, \boldsymbol{u}) = &\frac{1}{2}\left(\hat{\boldsymbol{a}} - \widehat{\mathbf{B}}^{\mathrm{PLS}}\boldsymbol{\theta}^{(t+1)} - \mathbf{R}\boldsymbol{\gamma}\right)^\top \mathbf{R}^{-1}\left(\hat{\boldsymbol{a}} - \widehat{\mathbf{B}}^{\mathrm{PLS}}\boldsymbol{\theta}^{(t+1)} - \mathbf{R}\boldsymbol{\gamma}\right) + q_\tau(\boldsymbol{\gamma}_1) \\ &+ \boldsymbol{u}^\top(\boldsymbol{\gamma} - \boldsymbol{\gamma}_1) + \frac{\rho}{2}\|\boldsymbol{\gamma} - \boldsymbol{\gamma}_1\|_2^2, \end{aligned} \tag{26}$$

where $\boldsymbol{u}$ represents the augmented Lagrangian multiplier and ρ is an additional tuning parameter introduced in ADMM. The ADMM algorithm updates $\boldsymbol{\gamma}$, $\boldsymbol{\gamma}_1$, and $\boldsymbol{u}$ sequentially as follows:

$$\boldsymbol{\gamma}^{(t+1)} = \arg\min_{\boldsymbol{\gamma}} \; Q(\boldsymbol{\gamma}, \boldsymbol{\gamma}_1^{(t)}, \boldsymbol{u}^{(t)}), \tag{27}$$

$$\boldsymbol{\gamma}_1^{(t+1)} = \arg\min_{\boldsymbol{\gamma}_1} \; Q(\boldsymbol{\gamma}^{(t+1)}, \boldsymbol{\gamma}_1, \boldsymbol{u}^{(t)}), \tag{28}$$

$$\boldsymbol{u}^{(t+1)} = \arg\min_{\boldsymbol{u}} \; Q(\boldsymbol{\gamma}^{(t+1)}, \boldsymbol{\gamma}_1^{(t+1)}, \boldsymbol{u}). \tag{29}$$

Each update has a close-form solution. For (27), the score function is

$$\hat{\boldsymbol{a}} - \widehat{\mathbf{B}}^{\mathrm{PLS}}\boldsymbol{\theta}^{(t+1)} + \mathbf{R}\boldsymbol{\gamma}^{(t+1)} + \boldsymbol{u}^{(t)} + \rho(\boldsymbol{\gamma}^{(t+1)} - \boldsymbol{\gamma}_1^{(t)}) = 0, \tag{30}$$

and hence

$$\boldsymbol{\gamma}^{(t+1)} = \left(\mathbf{R} + \rho\mathbf{I}\right)^{-1}\left(\hat{\boldsymbol{a}} - \widehat{\mathbf{B}}^{\mathrm{PLS}}\boldsymbol{\theta}^{(t+1)} - \boldsymbol{u}^{(t)} + \rho\boldsymbol{\gamma}_1^{(t)}\right). \tag{31}$$

As for (28), the score function is

$$q'_\tau(\boldsymbol{\gamma}_1^{t+1}) + \boldsymbol{u}^{(t)} + \rho(\boldsymbol{\gamma}_1^{(t+1)} - \boldsymbol{\gamma}^{(t+1)}) = 0, \tag{32}$$

and hence

$$\boldsymbol{\gamma}_1^{t+1} = \mathrm{mcp}_{\rho^{-1}\tau}(\boldsymbol{\gamma}^{(t+1)} + \rho^{-1}\boldsymbol{u}^{(t)}), \tag{33}$$

where

$$\mathrm{mcp}_\tau(x) = \begin{cases} \frac{a}{a-1}\mathtt{soft}_\tau(x), & \text{if } |x| \le \tau a, \\ x, & \text{if } |x| > \tau a, \end{cases} \tag{34}$$

where $\mathtt{soft}_\tau(x) = \mathtt{sign}(x)(|x| - \tau)_+$ is known as the soft-thresholding operator and $a = 3$. As for (29), the gradient update is applied:

$$\boldsymbol{u}^{(t+1)} = \boldsymbol{u}^{(t)} - \rho(\boldsymbol{\gamma}^{t+1} - \boldsymbol{\gamma}_1^{t+1}). \tag{35}$$

Note that $\hat{\boldsymbol{\gamma}}$ will not be strictly sparse. Therefore, we use $\hat{\boldsymbol{\gamma}}_1$ as the final output once the entire minimization process converges.

3.4 Deduction of expectation of estimating equation

We incorporate the unbiased estimating function into the above estimating procedure by looking at the score function of (19). Specifically, let $\mathcal{M}_\theta^{(t)} = \{j, \theta_j^{(t)} \neq 0\}$ and $\mathcal{M}_\gamma^{(t)} = \{s, \gamma_s^{(t)} \neq 0\}$ be two working active sets in the tth iteration. The joint score function (denoted as $\mathrm{U}(\cdot)$) of $\boldsymbol{\theta}^{(t)}_{\mathcal{M}_\theta^{(t)}}$ and $\boldsymbol{\gamma}^{(t)}_{\mathcal{M}_\gamma^{(t)}}$ is

$$\mathbf{0} = \mathrm{U}\begin{pmatrix} \boldsymbol{\theta}^{(t)}_{\mathcal{M}_\theta^{(t)}} \\ \boldsymbol{\gamma}^{(t)}_{\mathcal{M}_\gamma^{(t)}} \end{pmatrix} = -\begin{pmatrix} \widehat{\mathbf{B}}^{\mathrm{PLS}\top}_{\mathcal{M}_\theta^{(t)}} \mathbf{R}^{-1}\left(\hat{\boldsymbol{a}} - \widehat{\mathbf{B}}^{\mathrm{PLS}}_{\mathcal{M}_\theta^{(t)}} \boldsymbol{\theta}^{(t)}_{\mathcal{M}_\theta^{(t)}} - \mathbf{R}_{\mathcal{M}_\gamma^{(t)}} \boldsymbol{\gamma}^{(t)}_{\mathcal{M}_\gamma^{(t)}}\right) \\ \mathbf{I}^\top_{\mathcal{M}_\gamma^{(t)}}\left(\hat{\boldsymbol{a}} - \widehat{\mathbf{B}}^{\mathrm{PLS}}_{\mathcal{M}_\theta^{(t)}} \boldsymbol{\theta}^{(t)}_{\mathcal{M}_\theta^{(t)}} - \mathbf{R}_{\mathcal{M}_\gamma^{(t)}} \boldsymbol{\gamma}^{(t)}_{\mathcal{M}_\gamma^{(t)}}\right) \end{pmatrix} + \begin{pmatrix} \frac{\partial p_{\boldsymbol{\lambda}_\theta}}{\partial \boldsymbol{\theta}^{(t)}_{\mathcal{M}_\theta^{(t)}}} \\ \frac{\partial q_{\boldsymbol{\lambda}_\gamma}}{\partial \boldsymbol{\gamma}^{(t)}_{\mathcal{M}_\gamma^{(t)}}} \end{pmatrix} \tag{36}$$

where $\widehat{\mathbf{B}}^{\mathrm{PLS}}_{\mathcal{M}_\theta^{(t)}}$ and $\mathbf{I}_{\mathcal{M}_\gamma^{(t)}}$ are the sub-matrices of $\widehat{\mathbf{B}}^{\mathrm{PLS}}$ and $\mathbf{I}_m$ with columns being $\mathcal{M}_\theta^{(t)}$ and $\mathcal{M}_\gamma^{(t)}$, respectively. To simplify the deduction, we assume that the penalty functions $p_{\boldsymbol{\lambda}_\theta}(\cdot)$ and $q_{\boldsymbol{\lambda}_\gamma}(\cdot)$ enjoy the oracle property (Fan and Li, 2001) and hence $\partial p_{\boldsymbol{\lambda}_\theta}/\partial \boldsymbol{\theta}^{(t)}_{\mathcal{M}_\theta^{(t)}} \approx 0$ and $\partial q_{\boldsymbol{\lambda}_\gamma}/\partial \boldsymbol{\gamma}^{(t)}_{\mathcal{M}_\gamma^{(t)}} \approx 0$. As a result,

$$\boldsymbol{\gamma}^{(t)}_{\mathcal{M}_\gamma^{(t)}} = \mathbf{R}^{-1}_{\mathcal{M}_\gamma^{(t)}\mathcal{M}_\gamma^{(t)}} \mathbf{I}^\top_{\mathcal{M}_\gamma^{(t)}} (\hat{\boldsymbol{a}} - \widehat{\mathbf{B}}^{\mathrm{PLS}}_{\mathcal{M}_\theta^{(t)}} \boldsymbol{\theta}^{(t)}_{\mathcal{M}_\theta^{(t)}}), \tag{37}$$

where $\mathbf{R}^{-1}_{\mathcal{M}_\gamma^{(t)}\mathcal{M}_\gamma^{(t)}}$ is the sub-matrix of $\mathbf{R}$ with rows and columns in $\mathcal{M}_\gamma^{(t)}$. The marginal score function of $\boldsymbol{\theta}^{(t)}_{\mathcal{M}_\theta^{(t)}}$ is thus given by

$$\mathrm{U}(\boldsymbol{\theta}^{(t)}_{\mathcal{M}_\theta^{(t)}}) = -\widehat{\mathbf{B}}^{\mathrm{PLS}\top}_{\mathcal{M}_\theta^{(t)}} \mathbf{R}^{-1}\left(\hat{\boldsymbol{a}} - \widehat{\mathbf{B}}^{\mathrm{PLS}}_{\mathcal{M}_\theta^{(t)}} \boldsymbol{\theta}^{(t)}_{\mathcal{M}_\theta^{(t)}} - \mathbf{R}_{\mathcal{M}_\gamma^{(t)}} \mathbf{R}^{-1}_{\mathcal{M}_\gamma^{(t)}\mathcal{M}_\gamma^{(t)}} \mathbf{I}^\top_{\mathcal{M}_\gamma^{(t)}} (\hat{\boldsymbol{a}} - \widehat{\mathbf{B}}^{\mathrm{PLS}}_{\mathcal{M}_\theta^{(t)}} \boldsymbol{\theta}^{(t)}_{\mathcal{M}_\theta^{(t)}})\right). \tag{38}$$

According to the principle of unbiased estimating function, if the expectation of $\mathrm{U}(\boldsymbol{\theta}^{(t)}_{\mathcal{M}_\theta^{(t)}})$ is not zero, the corresponding estimate $\boldsymbol{\theta}^{(t)}_{\mathcal{M}_\theta^{(t)}}$ such that $\mathrm{U}(\boldsymbol{\theta}^{(t)}_{\mathcal{M}_\theta^{(t)}}) = \mathbf{0}$ is subject to bias. We define that

$$\hat{\boldsymbol{a}} = \mathbf{R}\boldsymbol{\alpha} + \mathbf{R}^{\frac{1}{2}} \boldsymbol{e}_\alpha, \quad \hat{\mathbf{B}}^{\mathrm{PLE}} = \mathbf{R}\mathbf{B} + \mathbf{R}^{\frac{1}{2}} \mathbf{E}_B, \tag{39}$$

where

$$(\mathbf{E}_B, \boldsymbol{e}_\alpha) \sim \mathcal{MN}(\mathbf{0}, \mathbf{I}, \boldsymbol{\Delta}_{\alpha B}), \tag{40}$$

$\mathcal{MN}(\mathbf{U}, \boldsymbol{\Sigma}_1, \boldsymbol{\Sigma}_2)$ refers to the matrix-normal distribution (Dutilleul, 1999): for any matrix $\mathbf{X} \sim \mathcal{MN}(\mathbf{U}, \boldsymbol{\Sigma}_1, \boldsymbol{\Sigma}_2)$, $\mathrm{E}(\mathbf{X}) = \mathbf{U}$ and $\mathrm{cov}(\mathrm{vec}(\mathbf{X})) = \boldsymbol{\Sigma}_1 \otimes \boldsymbol{\Sigma}_2$. Here, $\boldsymbol{\Delta}_{\alpha B}$ is still the estimation error covariance matrix, whose diagonal elements are $n_1^{-1}, \dots, n_p^{-1}, n_0^{-1}$, and offdiagonal elements are

$$\Delta_{\beta_j\beta_k} = \frac{n_{jk}}{n_j n_k} \mathrm{cov}(x_{ij}, x_{ik}), \quad \Delta_{\beta_j\alpha} = \frac{n_{j0}}{n_j n_0} \mathrm{cov}(x_{ij}, y_i), \tag{41}$$

where n_{jk} is the overlapping sample size between the GWAS cohorts of x_{ij} and x_{ik}. Based on these variables, we can re-express $\mathrm{U}(\boldsymbol{\theta}^{(t)}_{\mathcal{M}^{(t)}_\theta})$ as

$$
\begin{aligned}
\mathrm{U}(\boldsymbol{\theta}^{(t)}_{\mathcal{M}^{(t)}_\theta}) &= (\mathbf{R}\mathbf{B}_{\mathcal{M}^{(t)}_\theta}\boldsymbol{\theta}_{\mathcal{M}^{(t)}_\theta} + \mathbf{R}^{\frac{1}{2}}\mathbf{E}_B\mathcal{M}^{(t)}_\theta)\mathbf{R}^{-1}(\mathbf{R}^{\frac{1}{2}}\boldsymbol{e}_\alpha - \mathbf{R}^{\frac{1}{2}}\mathbf{E}_{B\mathcal{M}^{(t)}_\theta}\boldsymbol{\theta}_{\mathcal{M}^{(t)}_\theta}) \\
&\quad + (\mathbf{R}\mathbf{B}_{\mathcal{M}^{(t)}_\theta}\boldsymbol{\theta}_{\mathcal{M}^{(t)}_\theta} + \mathbf{R}^{\frac{1}{2}}\mathbf{E}_B\mathcal{M}^{(t)}_\theta)\mathbf{R}^{-1}\mathbf{P}_{\boldsymbol{\gamma}^{(t)}}(\mathbf{R}^{\frac{1}{2}}\boldsymbol{e}_\alpha - \mathbf{R}^{\frac{1}{2}}\mathbf{E}_{B\mathcal{M}^{(t)}_\theta}\boldsymbol{\theta}_{\mathcal{M}^{(t)}_\theta}) \\
&= I_1 + I_2 + I_3 + I_4,
\end{aligned}
\tag{42}
$$

where $\mathbf{P}_{\boldsymbol{\gamma}^{(t)}} = \mathbf{R}^{-1}_{\mathcal{M}^{(t)}_\gamma\mathcal{M}^{(t)}_\gamma}\mathbf{I}^\top_{\mathcal{M}^{(t)}_\gamma}$, which can be sorted as

$$
\mathbf{P}^{\text{sorted according to } \mathcal{M}^{(t)}_\gamma}_{\boldsymbol{\gamma}^{(t)}} = \begin{pmatrix} \mathbf{I}_{\mathcal{M}^{(t)}_\gamma} & \mathbf{0} \\ \mathbf{0} & \mathbf{0} \end{pmatrix},
\tag{43}
$$

and

$$
\begin{aligned}
I_1 &= \mathbf{B}^\top_{\mathcal{M}^{(t)}_\theta}\mathbf{R}^{\frac{1}{2}}(\mathbf{R}^{\frac{1}{2}}\boldsymbol{e}_\alpha - \mathbf{R}^{\frac{1}{2}}\mathbf{E}_{B\mathcal{M}^{(t)}_\theta}\boldsymbol{\theta}_{\mathcal{M}^{(t)}_\theta}), \quad I_2 = \mathbf{E}^\top_{B\mathcal{M}^{(t)}_\theta}(\boldsymbol{e}_\alpha - \mathbf{E}_{B\mathcal{M}^{(t)}_\theta}\boldsymbol{\theta}_{\mathcal{M}^{(t)}_\theta}), \\
I_3 &= \mathbf{B}^\top_{\mathcal{M}^{(t)}_\theta}\mathbf{R}^{\frac{1}{2}}\mathbf{P}_{\boldsymbol{\gamma}^{(t)}}(\mathbf{R}^{\frac{1}{2}}\boldsymbol{e}_\alpha - \mathbf{R}^{\frac{1}{2}}\mathbf{E}_{B\mathcal{M}^{(t)}_\theta}\boldsymbol{\theta}_{\mathcal{M}^{(t)}_\theta}), \quad I_4 = \mathbf{E}^\top_{B\mathcal{M}^{(t)}_\theta}(\boldsymbol{e}_\alpha - \mathbf{E}_{B\mathcal{M}^{(t)}_\theta}\boldsymbol{\theta}_{\mathcal{M}^{(t)}_\theta}).
\end{aligned}
\tag{44}
$$

It is easy to see that $\mathrm{E}(I_1) = \mathrm{E}(I_3) = \mathbf{0}$ since the estimation error should be independent of the genetic effects. As for I_2 and I_4,

$$
\mathrm{E}(I_2) = m\Big(\boldsymbol{\Delta}_{\mathcal{M}^{(t)}_\theta}\boldsymbol{\theta}_{\mathcal{M}^{(t)}_\theta} - \boldsymbol{\delta}_{\mathcal{M}^{(t)}_\theta}\Big), \quad \mathrm{E}(I_4) = ||\boldsymbol{\gamma}^{(t)}_{\mathcal{M}^{(t)}_\gamma}||_0\Big(\boldsymbol{\Delta}_{\mathcal{M}^{(t)}_\theta}\boldsymbol{\theta}_{\mathcal{M}^{(t)}_\theta} - \boldsymbol{\delta}_{\mathcal{M}^{(t)}_\theta}\Big),
\tag{45}
$$

where $\boldsymbol{\Delta}_{\mathcal{M}^{(t)}_\theta}$ is the sub-matrix of $\boldsymbol{\Delta}_{\alpha B}$ with indices of columns in $\mathcal{M}^{(t)}_\theta$, and $\boldsymbol{\delta}_{\mathcal{M}^{(t)}_\theta}$ is a sub-vector of the last column of $\boldsymbol{\Delta}_{\alpha B}$ with indices of elements in $\mathcal{M}^{(t)}_\theta$. Thus, we prove that

$$
\mathrm{E}\Big(\mathrm{U}(\boldsymbol{\theta}^{(t)}_{\mathcal{M}^{(t)}_\theta})\Big) = m_{\text{valid}}\Big(\boldsymbol{\Delta}_{\mathcal{M}^{(t)}_\theta}\boldsymbol{\theta}_{\mathcal{M}^{(t)}_\theta} - \boldsymbol{\delta}_{\mathcal{M}^{(t)}_\theta}\Big),
\tag{46}
$$

where $m_{\text{valid}} = m - ||\boldsymbol{\gamma}^{(t)}_{\mathcal{M}^{(t)}_\gamma}||_0$ is the number of valid IVs.

After obtaining the expectation of $\mathrm{U}(\boldsymbol{\theta}^{(t)}_{\mathcal{M}^{(t)}_\theta})$, the bias-corrected estimating equation of $\boldsymbol{\theta}^{(t)}_{\mathcal{M}^{(t)}_\theta}$ is

$$
\mathrm{U}_{\text{BEE}}(\boldsymbol{\theta}^{(t)}_{\mathcal{M}^{(t)}_\theta}) = \mathrm{U}(\boldsymbol{\theta}^{(t)}_{\mathcal{M}^{(t)}_\theta}) - m_{\text{valid}}\Big(\boldsymbol{\Delta}_{\mathcal{M}^{(t)}_\theta}\boldsymbol{\theta}_{\mathcal{M}^{(t)}_\theta} - \boldsymbol{\delta}_{\mathcal{M}^{(t)}_\theta}\Big),
\tag{47}
$$

and then cis-MRBEE re-updates the sub-vector $\boldsymbol{\theta}^{(t)}_{\mathcal{M}^{(t)}_\theta}$ by

$$
\boldsymbol{\theta}^{(t)}_{\mathcal{M}^{(t)}_\theta} = \Big(\widehat{\mathbf{B}}^{\text{PLS}\top}_{\mathcal{M}^{(t)}_\theta}\mathbf{R}^{-1}\widehat{\mathbf{B}}^{\text{PLS}}_{\mathcal{M}^{(t)}_\theta} - m_{\text{valid}}\boldsymbol{\Delta}_{\mathcal{M}^{(t)}_\theta}\Big)^{-1}\Big(\widehat{\mathbf{B}}^{\text{PLS}\top}_{\mathcal{M}^{(t)}_\theta}\mathbf{R}^{-1}(\hat{\boldsymbol{a}} - \mathbf{R}\boldsymbol{\gamma}^{(t)}) - m_{\text{valid}}\boldsymbol{\delta}_{\mathcal{M}^{(t)}_\theta}\Big).
\tag{48}
$$

The updates (20), (21), and (48) are iterated until $\boldsymbol{\theta}^{(t+1)}$ and $\boldsymbol{\gamma}^{(t+1)}$ get convergence, respectively denoted as $\hat{\boldsymbol{\theta}}$ and $\hat{\boldsymbol{\gamma}}$.

Cis-MRBEE requires the users to input the correlation matrix of the estimation errors, i.e.,

$$
\mathbf{R}_{xy} = \mathrm{cor}((\mathbf{E}_B, \boldsymbol{e}_\alpha)),
$$

which can be estimated by the statistically insignificant variants. Let $F_{i1}, \ldots, F_{iM}$ be M independent genetic variants that are not associated with a trait with GWAS effect estimate $\hat{b}^*_{jk} = \sum_{i=1}^{n_k} F_{ij}X_{ik}/n_k$. A consistent estimate of $\mathbf{R}_{xy}$ is

$$
\widehat{\mathbf{R}}_{xy} = \widehat{\mathrm{cor}}(\widehat{B}^*_i)
\tag{49}
$$

where $\widehat{B}^*_i = (\hat{\boldsymbol{b}}^*_1, \ldots, \hat{\boldsymbol{b}}^*_p, \hat{\boldsymbol{a}}^*)$. Please see our previous works (Lorincz-Comi et al., 2024; Yang et al., 2024) for more details.

3.5 Dealing with credible sets using discrete differential penalty

Using SuSiE for sparse prediction can lead to duplicated predicted exposures in cis-MVMR. This occurs when the sample size is small or due to specific local genetic architectures, where only one xQTL is identified by SuSiE, making others statistically indistinguishable. This issue is common in fine-mapping, and SuSiE groups these variants into a credible set and represents them with a single effect. However, directly extending the concept of credible sets to cis-MVMR presents technical challenges. To overcome this, we suggest applying the following discrete differential penalty to the credible set with two or more exposures:

$$d^0_{\lambda_s}(\mathrm{CS}_s) = \frac{\lambda_s}{2}\sum_{j,s\in\mathrm{CS}_s}(\hat{\boldsymbol{\beta}}^{\mathrm{PLS}}_j\theta_j - \hat{\boldsymbol{\beta}}^{\mathrm{PLS}}_s\theta_s)^\top\mathbf{R}^{-1}(\hat{\boldsymbol{\beta}}^{\mathrm{PLS}}_j\theta_j - \hat{\boldsymbol{\beta}}^{\mathrm{PLS}}_s\theta_s) \tag{50}$$

where CS_s is the sth credible set given by SuSiE, λ_s is a tuning parameter penalizing the sequential differences of causal effects of exposures in the CS_s. This penalty try to penalize the difference of predicted effects of exposures in the same credible set, which mimics the single effect defined by SuSiE. However, it is not easy to deal with in implementation, and hence we try to simplify it as

$$d^0_{\lambda_s}(\mathrm{CS}_s) \approx d_{\lambda_s}(\mathrm{CS}_s) = \frac{\lambda_s}{2}\sum_{j,t\in\mathrm{CS}_s}\left(\sqrt{\hat{\boldsymbol{\beta}}^{\mathrm{PLS}\top}_j\mathbf{R}\hat{\boldsymbol{\beta}}^{\mathrm{PLS}}_j\theta_j^2} - \sqrt{\hat{\boldsymbol{\beta}}^{\mathrm{PLS}\top}_s\mathbf{R}\hat{\boldsymbol{\beta}}^{\mathrm{PLS}}_s\theta_s^2}\right)^2, \tag{51}$$

where the difference between (50) and (51) is

$$d^0_{\lambda_s}(\mathrm{CS}_s) - d_{\lambda_s}(\mathrm{CS}_s) = 2\hat{\boldsymbol{\beta}}^{\mathrm{PLS}\top}_j\mathbf{R}^{-1}\hat{\boldsymbol{\beta}}^{\mathrm{PLS}}_s\theta_j\theta_s - 2\sqrt{\hat{\boldsymbol{\beta}}^{\mathrm{PLS}\top}_j\mathbf{R}\hat{\boldsymbol{\beta}}^{\mathrm{PLS}}_j}\sqrt{\hat{\boldsymbol{\beta}}^{\mathrm{PLS}\top}_s\mathbf{R}\hat{\boldsymbol{\beta}}^{\mathrm{PLS}}_s}|\theta_j||\theta_s|, \tag{52}$$

which will be ignorable if the predicted exposures are almost identical in the same credible set. Using $d_{\lambda_s}(\mathrm{CS}_s)$, it is easy to get its gradient with respective to $\boldsymbol{\theta}_{\mathcal{M}^{(t)}_\theta}$ is

$$\frac{\partial d_{\lambda_s}(\mathrm{CS}_s)}{\partial\boldsymbol{\theta}_{\mathcal{M}^{(t)}_\theta}} = \mathbf{D}^\top\mathbf{D}\boldsymbol{\theta}_{\mathcal{M}^{(t)}_\theta}. \tag{53}$$

The matrix $\mathbf{D}$ is a $p_0 \times \binom{p_0}{2}$ sparse operator encoding pairwise weighted differences of the parameter vector $\boldsymbol{\theta}_{\mathcal{M}^{(t)}_\theta}$, where p_0 here is the length of $\boldsymbol{\theta}_{\mathcal{M}^{(t)}_\theta}$. Each of its $\binom{p_0}{2}$ columns corresponds to a unique pair (i,j), $1 \le i < j \le p_0$, and contains nonzero entries only in the i-th and j-th rows. Specifically, if $\mathbf{s}$ is defined by $s_j = \sqrt{\hat{\boldsymbol{\beta}}^{\mathrm{PLS}\top}_j\mathbf{R}\hat{\boldsymbol{\beta}}^{\mathrm{PLS}}_j}$, then the (i,j)-th pairwise difference column in $\mathbf{D}$ can be formed as $(\cdots,\mathrm{sign}_i s_i,\cdots,-\mathrm{sign}_j s_j,\cdots)^\top$, where $\mathrm{sign}_i \in \{+1,-1\}$ adjusts the orientation of θ_i, $i \in \mathrm{CS}_s$. Thus, applying $\mathbf{D}$ to $\boldsymbol{\theta}_{\mathcal{M}^{(t)}_\theta}$ produces a vector of all signed, scaled pairwise differences. Furthermore, the unbiased estimating function (47) incorporated with this penalty will be

$$\mathrm{U_{BEE}}(\boldsymbol{\theta}^{(t)}_{\mathcal{M}^{(t)}_\theta}) = \mathrm{U}(\boldsymbol{\theta}^{(t)}_{\mathcal{M}^{(t)}_\theta}) - m_{\mathrm{valid}}\left(\boldsymbol{\Delta}_{\mathcal{M}^{(t)}_\theta}\boldsymbol{\theta}_{\mathcal{M}^{(t)}_\theta} - \boldsymbol{\delta}_{\mathcal{M}^{(t)}_\theta}\right) + \lambda_s\mathbf{D}^\top\mathbf{D}\boldsymbol{\theta}^{(t)}_{\mathcal{M}^{(t)}_\theta}. \tag{54}$$

Thus, the sub-vector $\boldsymbol{\theta}^{(t)}_{\mathcal{M}^{(t)}_\theta}$ by

$$\boldsymbol{\theta}^{(t)}_{\mathcal{M}^{(t)}_\theta} = \left(\widehat{\mathbf{B}}^{\mathrm{PLS}\top}_{\mathcal{M}^{(t)}_\theta}\mathbf{R}^{-1}\widehat{\mathbf{B}}^{\mathrm{PLS}}_{\mathcal{M}^{(t)}_\theta} - m_{\mathrm{valid}}\boldsymbol{\Delta}_{\mathcal{M}^{(t)}_\theta} + \lambda_s\mathbf{D}^\top\mathbf{D}\right)^{-1}\left(\widehat{\mathbf{B}}^{\mathrm{PLS}\top}_{\mathcal{M}^{(t)}_\theta}\mathbf{R}^{-1}(\hat{\boldsymbol{a}} - \mathbf{R}\boldsymbol{\gamma}^{(t)}) - m_{\mathrm{valid}}\boldsymbol{\delta}_{\mathcal{M}^{(t)}_\theta}\right). \tag{55}$$

3.6 Covariance matrix of causal effect estimate

Here, we provide two methods to estimate the covariance matrix of causal effect estimate $\hat{\boldsymbol{\theta}}_{\mathcal{M}_{\hat{\theta}}}$. The first method is the standard sandwich formula. Let

$$\boldsymbol{\epsilon} = \hat{\boldsymbol{a}} - \widehat{\mathbf{B}}^{\mathrm{PLS}}_{\mathcal{M}_{\hat{\theta}}}\hat{\boldsymbol{\theta}}_{\mathcal{M}_{\hat{\theta}}} - \mathbf{R}\hat{\boldsymbol{\alpha}}. \tag{56}$$

Then the covariance matrix of $\hat{\boldsymbol{\theta}}_{\mathcal{M}_{\hat{\theta}}}$ is

$$\operatorname{cov}(\hat{\boldsymbol{\theta}}_{\mathcal{M}_{\hat{\theta}}}) = \mathbf{H}_{\mathcal{M}_{\hat{\theta}}}^{-1} \widehat{\mathbf{B}}_{\mathcal{M}_{\hat{\theta}}}^{\text{PLS}\top} \mathbf{R}^{-1} \operatorname{diag}(\boldsymbol{\epsilon}^2) \mathbf{R}^{-1} \widehat{\mathbf{B}}_{\mathcal{M}_{\hat{\theta}}}^{\text{PLS}} \mathbf{H}_{\mathcal{M}_{\hat{\theta}}}^{-1} \tag{57}$$

where

$$\mathbf{H}_{\mathcal{M}_{\hat{\theta}}} = \widehat{\mathbf{B}}_{\mathcal{M}_{\hat{\theta}}}^{\text{PLS}\top} \mathbf{R}^{-1} \widehat{\mathbf{B}}_{\mathcal{M}_{\hat{\theta}}}^{\text{PLS}} - m_{\text{valid}} \boldsymbol{\Delta}_{\mathcal{M}_{\hat{\theta}}} + \lambda_s \mathbf{D}^\top \mathbf{D}. \tag{58}$$

When $\boldsymbol{\beta}_j$ contains many non-zero but small-scale informative variants, these variants may not be identified by SuSiE, resulting in what are known as infinitesimal effects (Cui et al., 2024). The mathematical model for the infinitesimal effect is:

$$\hat{\boldsymbol{a}} = \mathbf{R}\left(\sum_{j=1}^{p} \boldsymbol{\beta}_j \theta_j + \boldsymbol{\gamma} \right) + \underbrace{\mathbf{R}\boldsymbol{\upsilon} + \mathbf{R}^{\frac{1}{2}} \boldsymbol{e}_\alpha}_{\text{residual term}}, \tag{59}$$

where $\boldsymbol{e}_\alpha \sim \mathcal{N}(\mathbf{0}, \tau_e \mathbf{I}_m)$ and $\boldsymbol{\upsilon} \sim \mathcal{N}(\mathbf{0}, \tau_\upsilon \mathbf{I}_m)$ is an additional normal error term. We allow $\tau_e \geq 1$ to make the variance estimation more robust to potential uncontrolled uncertainties. Furthermore, restricted maximum likelihood (REML) is used to estimate τ_e and τ_υ, with the iterative estimation steps as follows:

$$\hat{\boldsymbol{\upsilon}} \leftarrow \left(\frac{\hat{\tau}_e}{\hat{\tau}_\upsilon} \mathbf{I}_m + \mathbf{R} \right)^{-1} \boldsymbol{\epsilon}, \tag{60}$$

$$\hat{\tau}_\upsilon \leftarrow m^{-1} \left[\|\hat{\boldsymbol{\upsilon}}\|_2^2 + \operatorname{tr}\left\{ \left(\frac{\hat{\tau}_e}{\hat{\tau}_\upsilon} \mathbf{I}_m + \mathbf{R} \right)^{-1} \right\} \right], \tag{61}$$

$$\hat{\tau}_e \leftarrow \frac{(\boldsymbol{\epsilon} - \mathbf{R}\hat{\boldsymbol{\upsilon}})^\top \mathbf{R}^{-1} (\boldsymbol{\epsilon} - \mathbf{R}\hat{\boldsymbol{\upsilon}})}{m - \operatorname{df}(\hat{\boldsymbol{\theta}}) - \operatorname{df}(\hat{\boldsymbol{\upsilon}}) - \operatorname{df}(\hat{\boldsymbol{\upsilon}})}, \tag{62}$$

where

$$\operatorname{df}(\hat{\boldsymbol{\theta}}) = \|\hat{\boldsymbol{\theta}}\|_0, \ \operatorname{df}(\hat{\boldsymbol{\upsilon}}) = \|\hat{\boldsymbol{\gamma}}\|_0, \ \operatorname{df}(\hat{\boldsymbol{\upsilon}}) = \operatorname{tr}\left\{ \left(\frac{\hat{\tau}_e}{\hat{\tau}_\upsilon} \mathbf{I}_m + \mathbf{R} \right)^{-1} \mathbf{R} \right\}. \tag{63}$$

Finally,

$$\operatorname{cov}(\hat{\boldsymbol{\theta}}_{\mathcal{M}_{\hat{\theta}}}) = \mathbf{H}_{\mathcal{M}_{\hat{\theta}}}^{-1} \widehat{\mathbf{B}}_{\mathcal{M}_{\hat{\theta}}}^{\text{PLS}\top} \mathbf{R}^{-1} \left(\hat{\tau}_e \mathbf{R} + \hat{\tau}_\upsilon \mathbf{R}^2 \right) \mathbf{R}^{-1} \widehat{\mathbf{B}}_{\mathcal{M}_{\hat{\theta}}}^{\text{PLS}} \mathbf{H}_{\mathcal{M}_{\hat{\theta}}}^{-1}. \tag{64}$$

3.7 Strength of horizontal pleiotropy

We should first clarify that the horizontal pleiotropy bias addressed in this study differs from that discussed in MR-Egger. In MR-Egger, horizontal pleiotropy bias is modeled as the intercept in a linear regression, with the corresponding hypothesis test assessing the significance of this intercept (Bowden et al., 2015). In contrast, the horizontal pleiotropy in our study aligns with the definition in MR-PRESSO, where it is characterized as "outliers": a deviation of a minority of IVs from the pathway formed by the majority of IVs (Verbanck et al., 2018).

To quantify the strength of this bias, we propose that it can be characterized using the following ratio:

$$V_\gamma = \frac{\hat{\boldsymbol{\gamma}}^\top \mathbf{R} \hat{\boldsymbol{\gamma}}}{(\hat{\mathbf{B}}^{\text{PLS}} \hat{\boldsymbol{\theta}} + \mathbf{R}\hat{\boldsymbol{\gamma}})^\top \mathbf{R}^{-1} (\hat{\mathbf{B}}^{\text{PLS}} \hat{\boldsymbol{\theta}} + \mathbf{R}\hat{\boldsymbol{\gamma}})}, \tag{65}$$

which is the ratio of the variance of horizontal pleiotropy to the combined variances of horizontal pleiotropy and exposure. Similarly, we can define:

$$\text{PVE}_\gamma = \frac{\hat{\boldsymbol{\gamma}}^\top \mathbf{R} \hat{\boldsymbol{\gamma}}}{\hat{\boldsymbol{a}}^\top \mathbf{R}^{-1} \hat{\boldsymbol{a}}}, \tag{66}$$

which is the proportion of variance explained (PVE) by horizontal pleiotropy. We applied the two aforementioned ratios to the real data of ANGPTL3 and found $V_\gamma = 0.1209$ and $\text{PVE}_\gamma = 0.1051$. This indicates that the variance explained by horizontal pleiotropy relative to the exposure is minimal.

3.8 Implementation

First, we introduce a method we term reliability shrinkage to address the issue of cis-MRBEE producing estimates with large variances when the estimation errors are substantial. Although these estimates are unbiased, their large variances make them unreliable and thus undesirable. To address this, Wu et al. (2024) proposed a spectral regularized estimator (formerly known as the adIVW estimator) to reduce variance. Instead of adopting this approach, we use a simpler method: scaling down the bias-correction term. This technique has already been employed in our estimation of genetic graph (EGG, Yang et al. (2024, Supplementary Materials)). Specifically, we proceed as follows: Specifically, we consider re-scaling the covariance matrix of estimation error by:

$$\boldsymbol{\Delta}_{\alpha B} \leftarrow \mathbf{L}\boldsymbol{\Delta}_{\alpha B}\mathbf{L}, \tag{67}$$

where $\mathbf{L}$ is a diagonal matrix. We consider let

$$L_{ii} = \begin{cases} 1, & \frac{\hat{\Sigma}_{\hat{\beta}_j\hat{\beta}_j} - \hat{\Sigma}_{\omega_j\omega_j}}{\hat{\Sigma}_{\hat{\beta}_j\hat{\beta}_j}} > r, \\ \frac{\frac{1}{r}\hat{\Sigma}_{\hat{\beta}_j\hat{\beta}_j} - \hat{\Sigma}_{\hat{\beta}_j\hat{\beta}_j}}{\hat{\Sigma}_{\omega_j\omega_j}}, & \frac{\hat{\Sigma}_{\hat{\beta}_j\hat{\beta}_j} - \hat{\Sigma}_{\omega_j\omega_j}}{\hat{\Sigma}_{\hat{\beta}_j\hat{\beta}_j}} \le r, \end{cases} \tag{68}$$

where $r \in (0, 1)$ is a threshold. Increase r will decrease L_{ii} and $L_{ii} \to 0$ if $r \to 1$. We suggest $r = 0.75$ in practice. Note that this reliability shrinkage will not reduce the degree of bias-correction of the exposures whose reliability ratio is larger than r, and hence we would rather call it adaptive bias-correction.

We use the extended Bayesian information criterion (EBIC, Chen and Chen (2012)) to select the tuning parameters L in SuSiE and τ in MCP. The EBIC formula is given by:

$$\mathrm{EBIC}(L, \tau) = m\log(\hat{\sigma}_\alpha^2) + (\log m + \mathrm{ebic}_L \log p)L + \log m(1 + \mathrm{ebic}_\tau)\mathrm{df}_{\boldsymbol{\gamma}}, \tag{69}$$

where $\mathrm{df}_{\boldsymbol{\gamma}} = ||\hat{\boldsymbol{\gamma}}||_0$ is the number of non-zero elements in $\hat{\boldsymbol{\gamma}}$, m is the number of IVs, p is the number of exposures, $\mathrm{ebic}_L \ge 0$ and $\mathrm{ebic}_\tau \ge 0$ are additional penalty parameters in EBIC, and

$$\hat{\sigma}_\alpha^2 = \frac{\boldsymbol{\epsilon}^\top \boldsymbol{\epsilon}}{m - L - \mathrm{df}_{\boldsymbol{\gamma}}} \tag{70}$$

The larger these parameters are, the higher the penalization applied to the corresponding terms. When both parameters are set to 0, EBIC reduces to the standard BIC. We recommend setting $\mathrm{ebic}_L = 0$ because p is typically small, making additional penalization unnecessary, while $\mathrm{ebic}_\tau = 2$ is suggested to impose a moderate penalty on τ.

We found that cis-MRBEE is not sensitive to the choice of ρ, likely because the optimal ρ depends on the Hessian matrix of the optimization, which, in cis-MRBEE, corresponds to $\mathbf{R}$ with diagonal elements all equal to 1. While we consider ρ as a user-adjustable tuning parameter, the method performs stably for $\rho \in [1, 10]$. We do not recommend selecting values outside this range, particularly overly small values for ρ. In addition, cis-MRBEE is very insensitive to the choice of λ_s in discrete differential penalty. One can chose arbitrary $\lambda_s \in (1, 1e5)$.

3.9 Algorithm

Thus, we can give the concrete how do we implement cis-MRBEE.

4 Supplementary Issues During Peer Review

We are grateful to three Reviewers for their valuable suggestions to improve this manuscript. Some of their comments have been incorporated into the latest version of the paper, while others, being relatively independent of the methodology presented in this work, are addressed here.

Algorithm 1 Algorithm of cis-MRBEE

Require:

Outcome GWAS effect vector $\hat{\boldsymbol{\alpha}}$ and the SE vector se($\hat{\boldsymbol{\alpha}}$)
Exposures xQTL effect matrix $\hat{\mathbf{B}}$ and the SE matrix se($\hat{\mathbf{B}}$)
The LD matrix of IVs $\mathbf{R}$
The correlation matrix of estimation errors $\boldsymbol{\Delta}_{\alpha B}$
The number of single effects L_{xQTL} used in selecting informative xQTL
A vector of candidates of numbers of single effects $\mathcal{L}_{\text{exposure}}$
A vector of candidates of tuning parameters in MCP $\mathcal{T}_{\text{pleiotropy}}$
EBIC factors ebic$_L$ and ebic$_\tau$
A scale of tuning parameter in differential penalty λ_s

Ensure:

Estimate $\hat{\mathbf{B}}^{\text{PLS}}$ using (7)
Obtain the SE matrix of $\hat{\mathbf{B}}^{\text{PLS}}$ using (9)
for $L \in \mathcal{L}_{\text{exposure}}$ **do**
    for $\tau \in \mathcal{T}_{\text{pleiotropy}}$ **do**
        repeat
            Update $\boldsymbol{\gamma}^{(t+1)}$ using (16)-(26)
            Update $\mathcal{M}_{\theta}^{(t+1)}$ using (13)
            Update $\boldsymbol{\theta}^{(t+1)}$ using (45)
        until convergence
        Calculate the EBIC value using (57) and 58
    end for
end for
Determine the best model according to the minimum EBIC.
Yield the corresponding $\hat{\boldsymbol{\theta}}$ and $\hat{\boldsymbol{\gamma}}$ of the best model
Estimate the SE vector of $\hat{\boldsymbol{\theta}}$ using (50)-(54)

4.1 Polygencity of human complex trait

The polygenicity of human complex diseases may reduce the robustness of cis-MRBEE, particularly at loci with regulatory hubs. We are conducting additional simulations to validate the robustness of cis-MRBEE with respect to polygenicity. Specifically, following Cui et al. (2024), we employ the infinitesimal model to characterize polygenicity:

$$
\begin{aligned}
\hat{\boldsymbol{\alpha}} &\sim \mathcal{N}\left(\mathbf{R}\left(\sum_{j=1}^{p}\boldsymbol{\beta}_j\theta_j + \boldsymbol{\gamma} + \boldsymbol{v}_\alpha\right), \sigma_\alpha^2\mathbf{R}\right), \\
\hat{\boldsymbol{b}}_j &\sim \mathcal{N}\left(\mathbf{R}(\boldsymbol{\beta}_j + \boldsymbol{v}_{\beta_j}), \sigma_{\beta_j}^2\mathbf{R}\right), \quad 1 \le j \le p,
\end{aligned} \tag{71}
$$

where $\boldsymbol{v}_\alpha, \boldsymbol{v}_{\beta_1}, \ldots, \boldsymbol{v}_{\beta_p}$ are called infinitesimal effects, which are a sort of vectors with independently and identically distributed (i.i.d.) Gaussian entries. In this simulation, the variances of the Gaussian random variables were set to 0.1 and 0.2 of the local heritability of the exposure and outcome, respectively. We considered four scenarios: no infinitesimal effects, infinitesimal effects in exposures only, infinitesimal effects in the outcome only, and infinitesimal effects in both exposure and outcome.

Our findings indicate that including infinitesimal effects in the model has a limited impact on the estimation and inference of causal effects for individual exposures. Therefore, we chose to evaluate performance using three joint metrics for all causal effect estimates: mean squared error (MSE), true negative (TN) rate, and true positive (TP) rate. A true negative event is defined as correctly identifying all non-causal exposures, while a true positive event is defined as correctly identifying causal exposures (consistent with the definitions of Type I error and power in the main text). The figure below illustrates these metrics when infinitesimal effects account for 0.1 of the local variance of exposures and outcomes. Notably, including minor infinitesimal effects has no significant impact on cis-MRBEE.

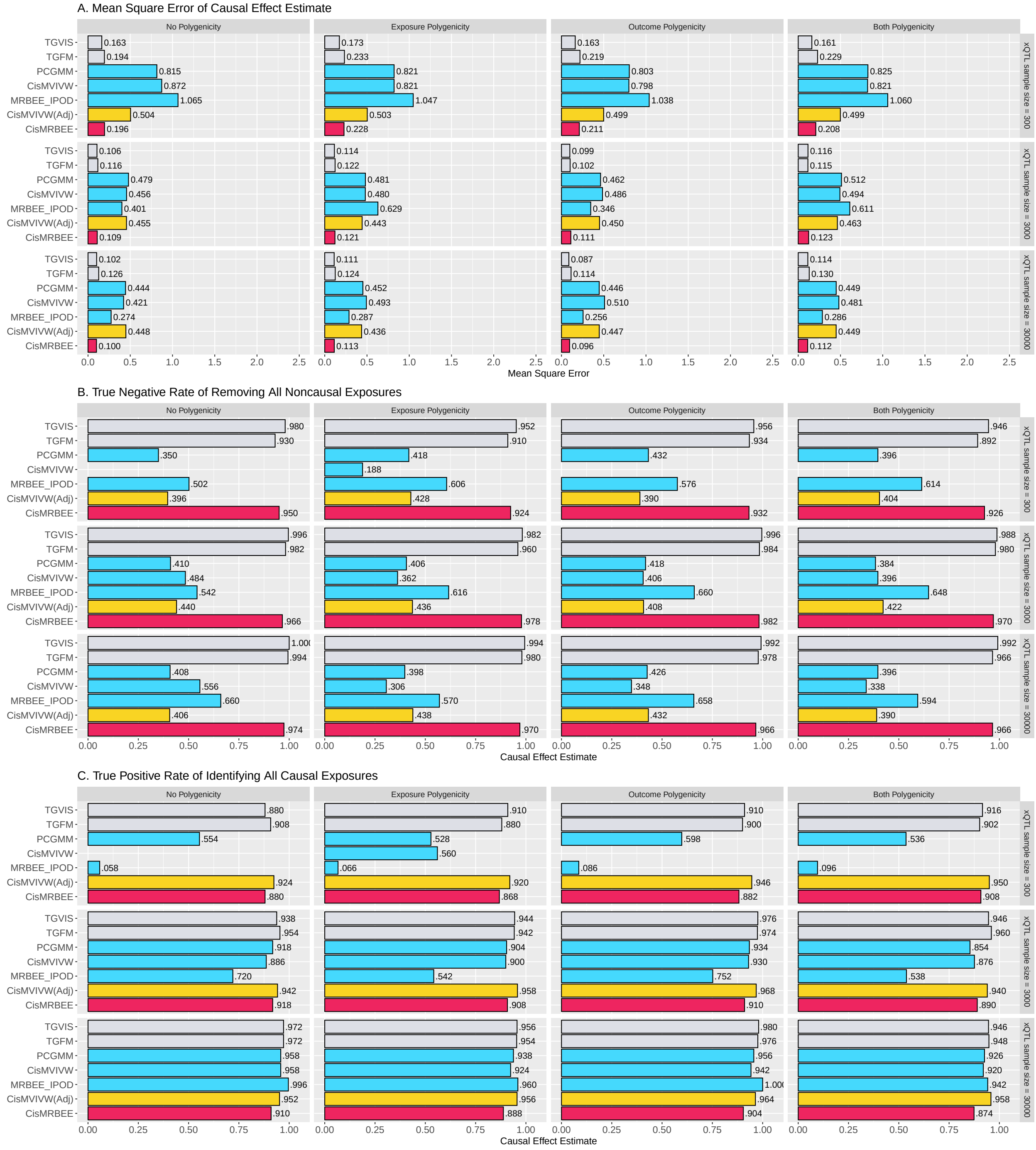

This figure Mean squared error (MSE), true negative (TN) rate, and true positive (TP) rate when the proportion of variance explained by infinitesimal effect is 0.1. A true negative event is defined as correctly identifying all non-causal exposures, while a true positive event is defined as correctly identifying causal exposures.

4.2 Computing Time

Outcome	Method	Clumping R2	IV Number	Sparse Prediction	Estimation
Triglyceride	CisMRBEE	No Clumping	6,450	128.933 secs	371.511 secs
Triglyceride	CisMVIVW	No Clumping	6,450		9.775283 secs
Triglyceride	PCGMM	No Clumping	6,450		70.44602 secs
Triglyceride	CisMRBEE	0.99	791	4.481 secs	55.633 secs
Triglyceride	CisMVIVW	0.99	791		0.4931264 secs
Triglyceride	PCGMM	0.99	791		10.1916 secs
Triglyceride	CisMRBEE	0.9	486	2.549 secs	32.281 secs
Triglyceride	CisMVIVW	0.9	486		0.04493284 secs
Triglyceride	PCGMM	0.9	486		7.500954 secs
Triglyceride	CisMRBEE	0.81	415	3.245 secs	26.675 secs
Triglyceride	CisMVIVW	0.81	415		0.03418541 secs
Triglyceride	PCGMM	0.81	415		8.694357 secs
Triglyceride	CisMRBEE	0.64	313	1.534 secs	8.993 secs
Triglyceride	CisMVIVW	0.64	313		0.01991844 secs
Triglyceride	PCGMM	0.64	313		10.82568 secs
Triglyceride	CisMRBEE	0.49	256	1.441 secs	7.343 secs
Triglyceride	CisMVIVW	0.49	256		0.0229001 secs
Triglyceride	PCGMM	0.49	256		9.548179 secs

This table illustrates the computing time of cis-MRBEE, cis-MVIVW, and PCGMM for different number of IVs.

We use ANGPTL3 real-world data to illustrate the impact of the number of instruments (m). Specifically, we divide the cis-MRBEE computation time into two parts: sparse prediction (informative variants selection) and estimation of the causal effect of exposure. From the above table, we observe that when the number of IVs is large (e.g., $m = 6450$), the total estimation time for cis-MRBEE is approximately 8 minutes, which is significantly higher than the two comparison methods. However, when m is reduced to 791, the estimation completes in just 1 minute. As m continues to decrease, the computational burden further reduces, approaching that of PCGMM.

We use simulations to investigate the impact of p. Specifically, we extend the $p = 10$ simulation presented in the main text to $p = 100$ by adding 90 noise exposures generated from the first 10 exposures (only the first and third exposures are causal). We first select informative variants for all 100 exposures and then incrementally include different subsets of exposures in cis-MRBEE, starting from all 100 exposures, then the first 90, and finally the first 10 exposures. We find that cis-MRBEE is not highly sensitive to the exposure dimension (p). This is primarily due to the following optimizations we implemented when developing the MRBEEX software package: 1) rewriting matrix multiplications in C++ using CppMatrix (https://github.com/harryyiheyang/CppMatrix), significantly improving computational efficiency, and 2) implementing a warm start strategy, where SuSiE's s_init is set to the fitted result from the previous iteration, reducing redundant computations.

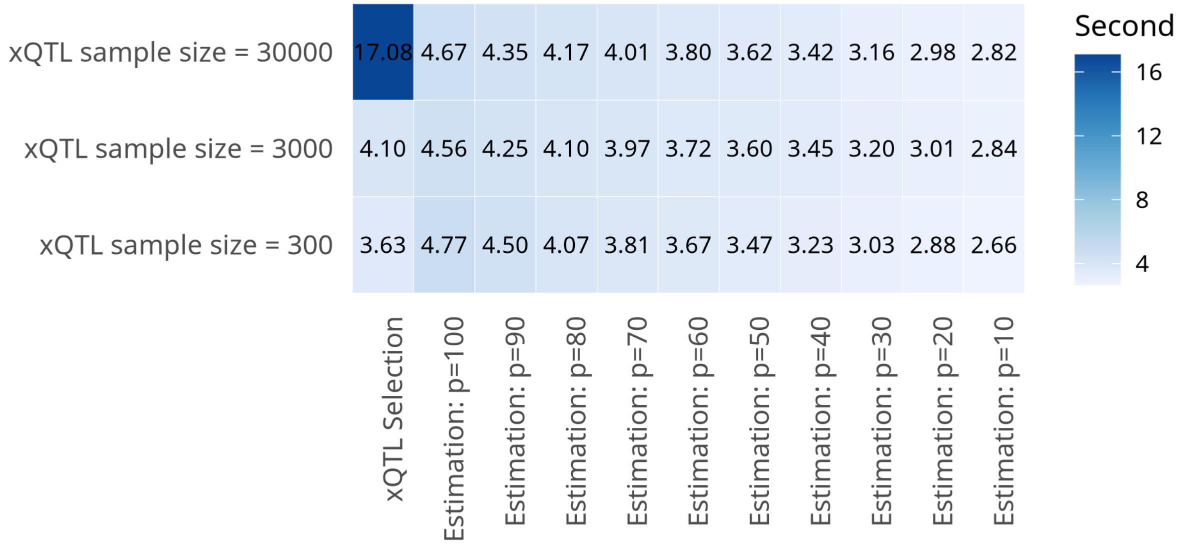

This figures illurate the computing time of cis-MRBEE for different number of exposures.

4.3 Influence of clumping threshold r^2

To investigate the impact of different C+T r^2 thresholds, we apply PLINK to perform C+T with `p1 = p2 = 5e5`, `kb = 1000`, and `r2 = 0.99, 0.9, 0.81, 0.64, 0.49`, obtaining different subsets of variants for cis-MVMR analysis. We use different sets of IVs for informative variants selection. However, when predicting exposures or assessing horizontal pleiotropy effects, we use the following formula for prediction:

$$\hat{\boldsymbol{b}}_j^{\text{PLS}} = \mathbf{R}_{\text{no clumping}} \hat{\boldsymbol{\beta}}_j, \tag{72}$$

where $\hat{\boldsymbol{\beta}}_j$ is a sparse vector with non-zero entries (genetic effects) estimated using a subset of IVs corresponding to a specific clumping parameter. We compare how the causal effect estimates change across different sets of IVs and how these variations affect the prediction of the outcome (Triglyceride GWAS).

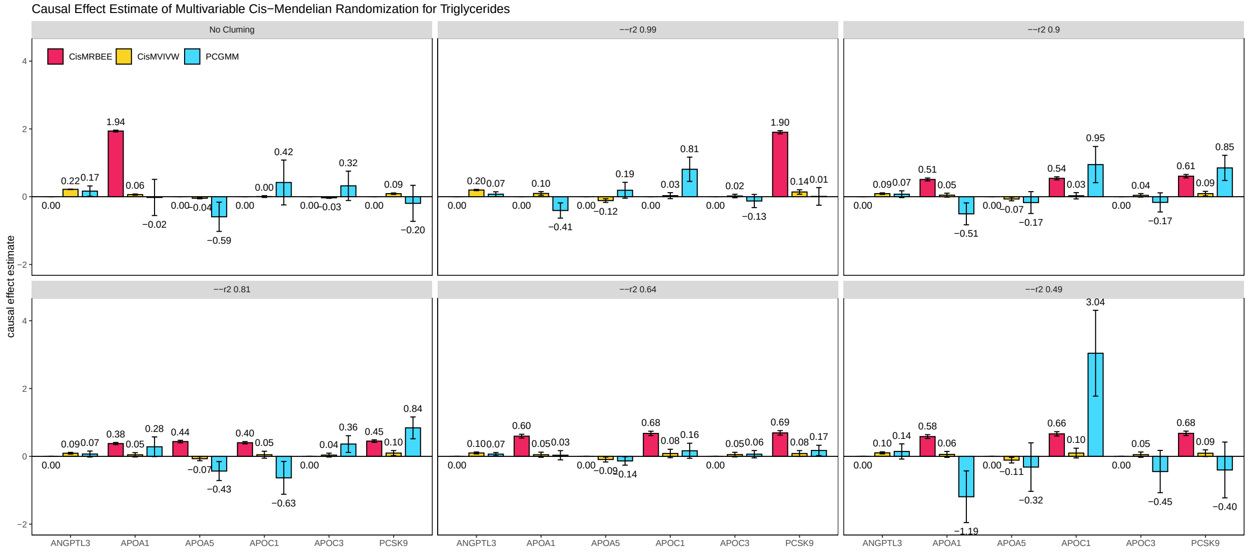

This figure shows the causal effect estimation of triglyceride in ANGPTL3 locus with varying r2 threshold in C+T.

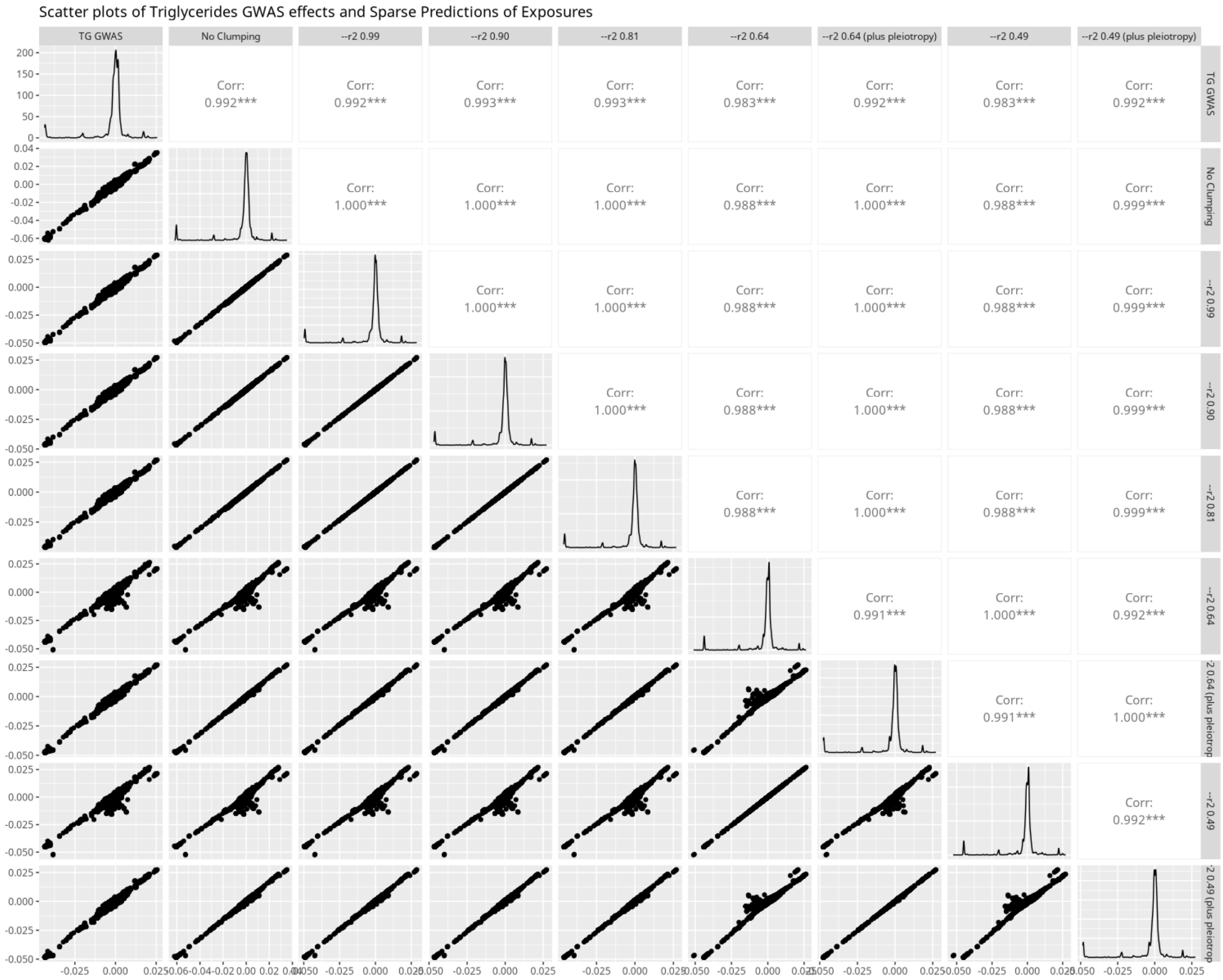

This figure shows the prediction of triglyceride GWAS in ANGPTL3 locus with varying r2 threshold in C+T. We generally show the scatter plot of TG GWAS $\hat{\boldsymbol{\alpha}}$ and the sparse prediction of exposures $\hat{\mathbf{B}}^{\text{PLS}}\hat{\boldsymbol{\theta}}$. "plus pleiotropy" means that we show the scatter plot of TG GWAS $\hat{\boldsymbol{\alpha}}$ and the sparse prediction of exposures and horizontal pleiotropy $\hat{\mathbf{B}}^{\text{PLS}}\hat{\boldsymbol{\theta}} + \mathbf{R}\hat{\boldsymbol{\gamma}}$.

We find that across different sets of IVs, the identified causal exposures may vary, but in most cases, they belong to the credible set of APOA1, APOC1, or PCSK9. For example, in the no clumping case, cis-MRBEE identifies APOA1 as the causal exposure; with `r2 = 0.99`, it identifies PCSK9 as causal; in other cases, it identifies a credible set containing APOA1, APOC1, and PCSK9 as causal exposures. In all scenarios, cis-MRBEE consistently identifies only one credible set, and the effect size of the single effect corresponding to this credible set remains nearly unchanged (~1.9). In our simulations, we also find that cis-MRBEE does not always identify all variables within a credible set. However, it consistently identified at least one variable from the credible set and would not cause inflated type-I errors and enjoyed high power.

Another notable feature we observe is that bias may arise when a set of IVs lacks a good proxy for causal variants, but this bias can be captured by horizontal pleiotropy. Specifically, when `--r2 = 0.64` or `0.49`, the prediction of exposures shows a clear inconsistency with TG. However, after incorporating the prediction of pleiotropy (i.e., $\mathbf{R}_{\text{no clumping}}\hat{\boldsymbol{\gamma}}$), the combined prediction of exposures and pleiotropy closely aligns with TG, similar to cases with weaker clumping thresholds. In other words, horizontal pleiotropy may not necessarily reflect non-exposure-mediated variants with some unknown biological functions. Instead, it may capture the bias introduced by missing causal variants. Fortunately, this bias does not affect the causal effect estimation of the exposure. This also explains why $V_\gamma = 0.1209$ and $\text{PVE}_\gamma = 0.1051$, both of which are relatively small values: they essentially capture the bias caused by the absence of causal variants or their good proxies.

4.4 Lack of causal variants

One consequence of using C+T is the potential removal of causal variants by mistake. However, the variants retained through C+T should include approximations of these causal variants, allowing them to be considered as informative variants.

To support this claim, we first conduct simulations to assess whether cis-MRBEE remains robust even when causal variants are not included in the model. We design the simulation as follows: specifically, we generate summary statistics from a model with $2m$ variants, where the LD matrix of these $2m$ variants is given by:

$$\mathbf{R}_{2m} = \begin{pmatrix} 1 & \rho \\ \rho & 1 \end{pmatrix} \otimes \mathbf{R} = \begin{pmatrix} \mathbf{R} & \rho\mathbf{R} \\ \rho\mathbf{R} & \mathbf{R} \end{pmatrix}, \tag{73}$$

where $\mathbf{R}$ is the LD matrix used in common simulations. For each exposure, we randomly generate a sparse vector $\boldsymbol{\beta}_j$ with three non-zero xQTL effects and compute the marginal effect as $\hat{\boldsymbol{b}}_j = \mathbf{R}_{2m}\boldsymbol{\beta}_j + \boldsymbol{e}_j$. However, when performing cis-MVMR analyses, we only include the first m variants in the corresponding methods. This means that each of the three causal variants has a 0.5 probability of being missing from the model, while a proxy variant with LD=ρ exists in the included set. We consider four scenarios with $\rho = 0.95, 0.9, 0.85, 0.8$, and the simulation results are as follows:

From the width of the boxplots, we observe that as ρ decreases, the width of the cis-MRBEE estimates increases, yet the estimates remain unbiased. Additionally, we find that as ρ decreases, the power of cis-MRBEE to detect the smaller effect $\theta_3 = 0.5$ visibly declines.

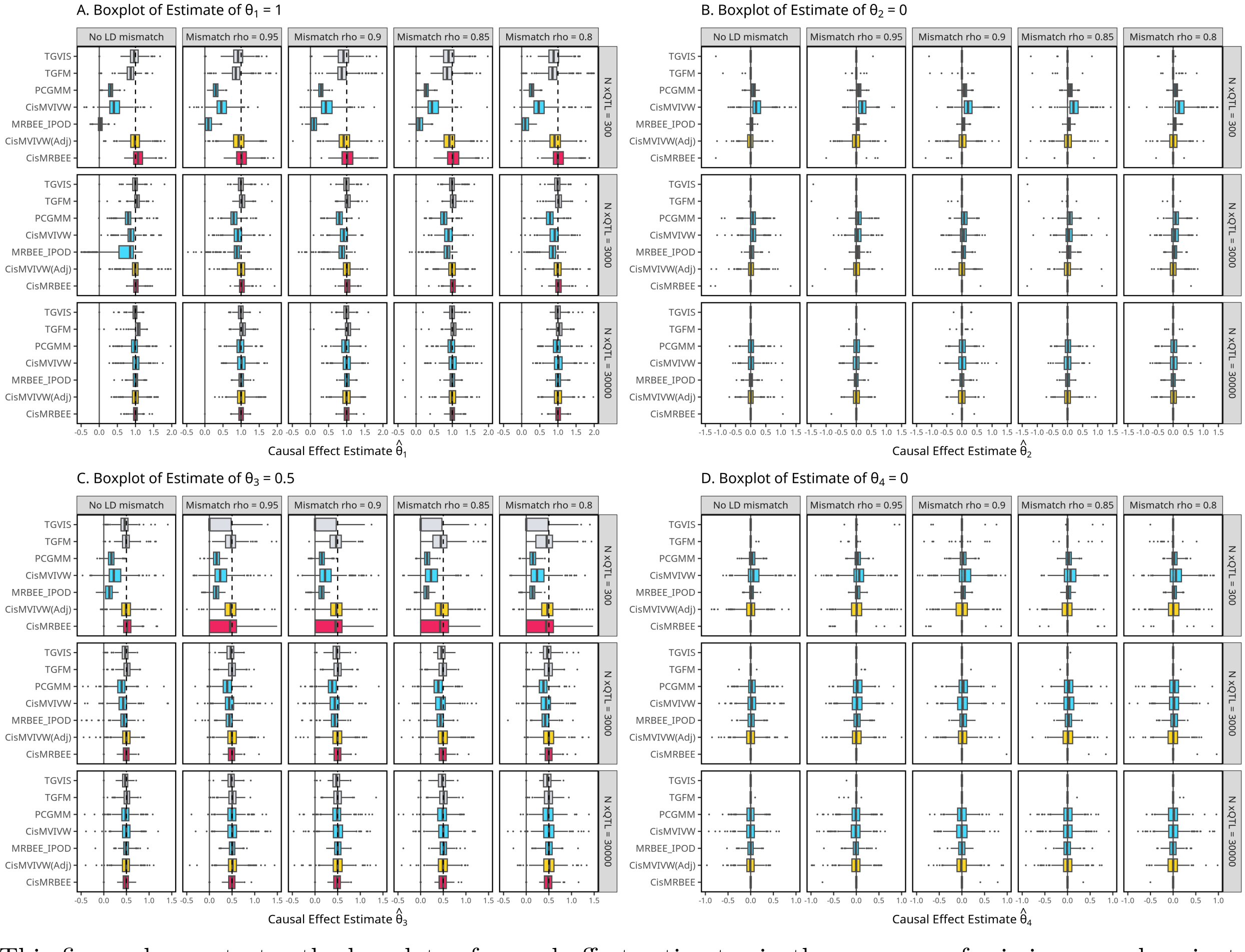

This figure demonstrates the boxplots of causal effect estimates in the presence of missing causal variants and their proxies with varying LD approximate coefficient ρ.

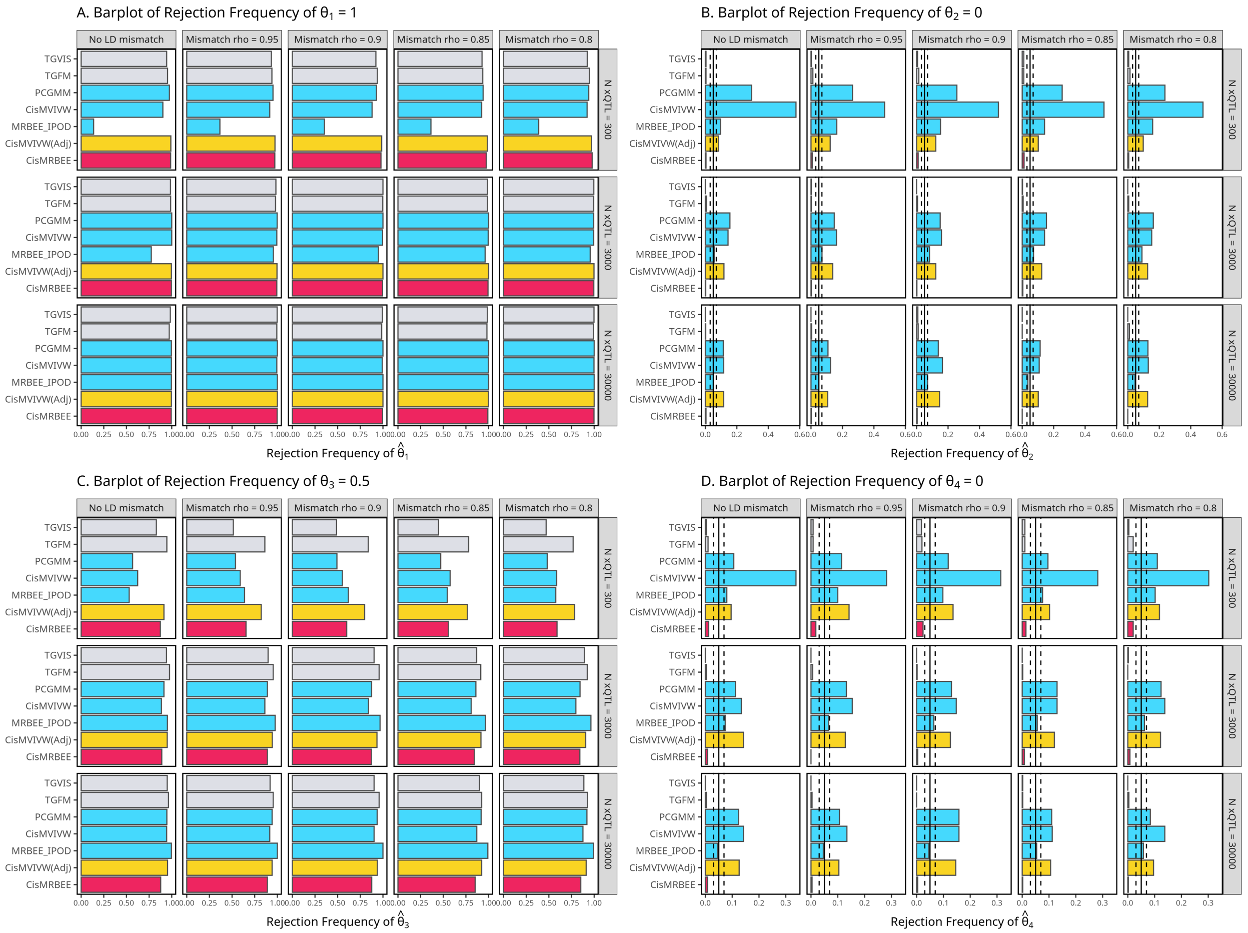

This figure demonstrates the boxplots of rejection frequencies of causal effect estimates in the presence of missing causal variants and their proxies with varying LD approximate coefficient ρ.

.

4.5 Irrepresentable condition

There is a viewpoint that to identify causal variants, one should avoid any clumping and include all variants from a locus in SuSiE or other fine-mapping methods. However, statistical principles do not actually support this viewpoint. Specifically, Zhao and Yu (2006) proved that for the model: $y = \boldsymbol{X}\boldsymbol{\beta} + \boldsymbol{e}$, lasso can consistently select the true model selection asymptotically only when

$$\|\mathbf{X}_{\mathcal{M}^c}^\top \mathbf{X}_{\mathcal{M}}(\mathbf{X}_{\mathcal{M}}^\top \mathbf{X}_{\mathcal{M}})\text{sign}(\boldsymbol{\beta}_{\mathcal{M}})\|_\infty < 1,$$

where $\mathcal{M}$ is the true model (the indices of the non-zero coefficients in $\boldsymbol{\beta}$). This condition is known as the irrepresentable condition, and it is quite strong in practice. Fan and Lv (2011) proved that for nonconvex penalties, this condition can be relaxed to:

$$\|\mathbf{X}_{\mathcal{M}^c}^\top \mathbf{X}_{\mathcal{M}}(\mathbf{X}_{\mathcal{M}}^\top \mathbf{X}_{\mathcal{M}})\text{sign}(\boldsymbol{\beta}_{\mathcal{M}})\|_\infty < \min(C_1 \frac{p'_\lambda(0+)}{p'_\lambda(\min_{j\in\mathcal{M}}(|\beta_j|))}, C_2 n^\alpha),$$

for certain fixed constants C_1, C_2 and α. However, this does not mean that variable selection consistency is always guaranteed whenever a nonconvex penalty (including SCAD, MCP, and some Bayesian priors) is used.

Although SuSiE introduces credible sets specifically to address this issue by grouping highly correlated variants into a single credible set and replacing their effects with single effects, it relies on a stepwise regression approach. Some studies have pointed out that when the number of causal variants within a locus is large,

SuSiE may fail to converge (Li et al., 2024). Therefore, we believe that if the goal is to predict exposures well, applying clumping to appropriately remove highly correlated variants is a reasonable approach.

4.6 Influence of local heritability

In MRBEE, we emphasize that estimation error is the key driver of weak instrument bias, and that the degree of weak instrument bias is dynamic: it depends on the relative scale (or variance) of estimation error and true effect sizes (Lorincz-Comi et al., 2024). In other words, modifying the heritability of the exposure or increasing the sample size of the exposure dataset can have an equivalent impact on weak instrument bias. For this reason, we did not explicitly consider varying local heritability in the first version of this manuscript.

Following your suggestion, we compared the boxplots of different estimators and barplots of rejection frequencies under hˆ2=0.3,0.2,0.1. We found that small changes in hˆ2 had minimal impact on the performance of different methods. When n_"xQTL" =300, cis-MRBEE suffered from lower power in these cases, so did the other methods.

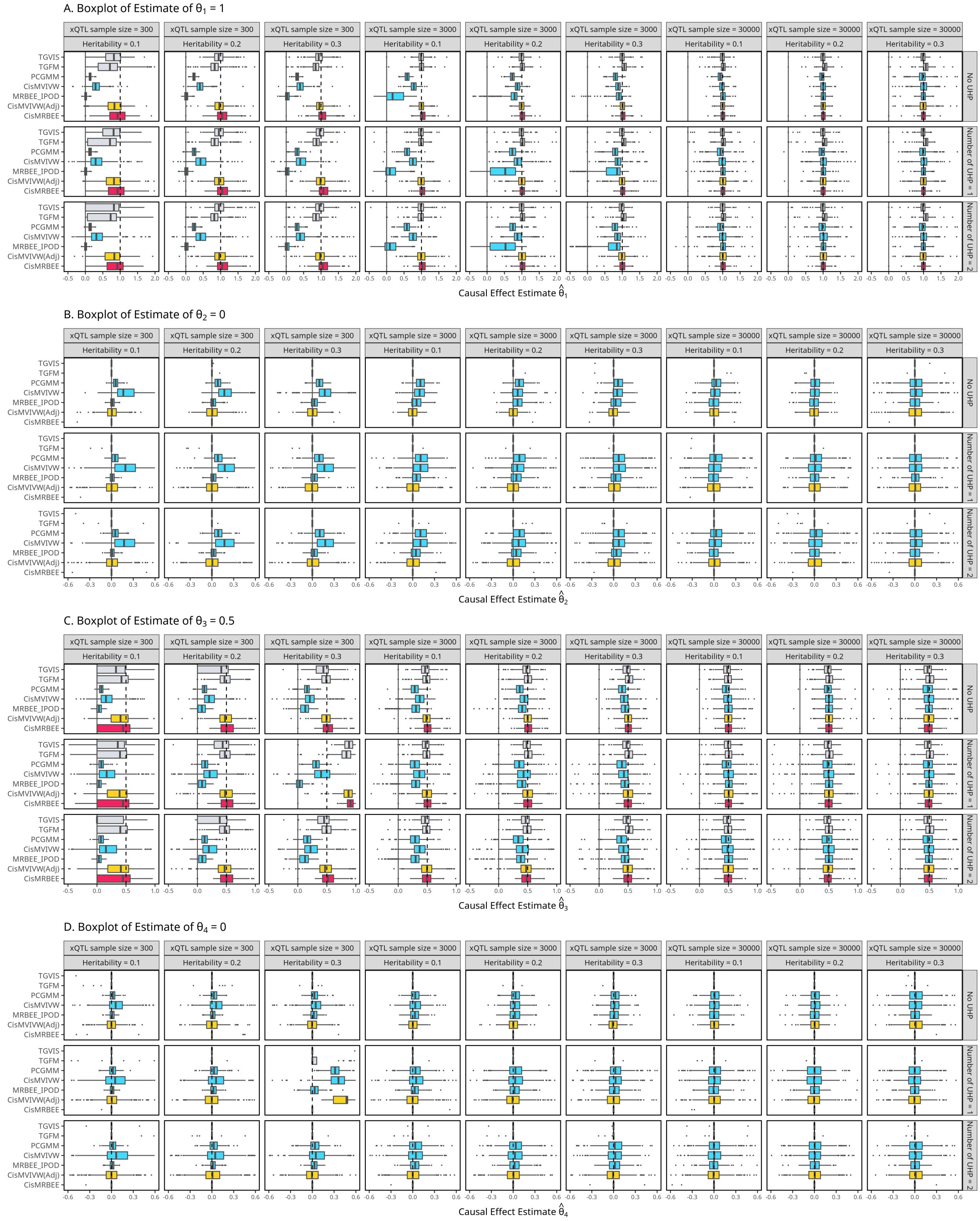

This figure exhibits the boxplots of causal effect estimates with varying local heritability of exposure.

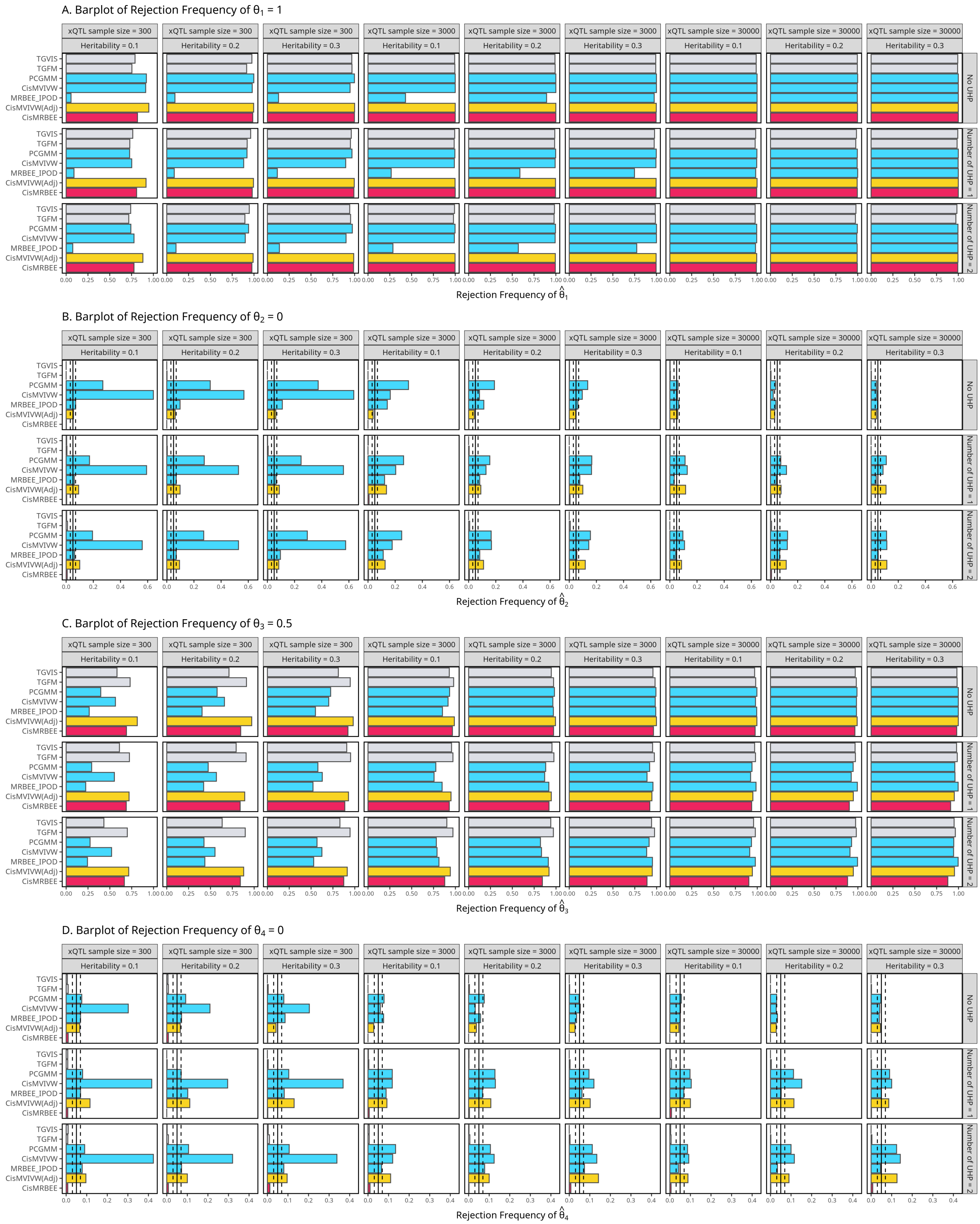

This figure exhibits the barplots of rejection frequencies of causal effect estimates with varying local heritability of exposure.

5 Settings in Real Data Analysis

5.1 C+T in cis-MR analysis

We recommend using C+T to filter out variants in high LD, which prevents the inclusion of numerous highly correlated or redundant variants in the analysis, which can unnecessarily complicate the model and result in multiple credible sets consisting of these variants. We evaluated the minimum P-value of each variant across exposures and outcome. In PLINK, we applied the C+T with the following parameters: –clump-kb 1000, –clump-p1 5E-5, –clump-p2 5E-5, and –clump-r2 0.64, with LD reference panels consisting of the 9,680 individuals and 9.3M variants from UKBB.

5.2 C+T in MVMR analysis using genome-wide significant IVs

We performed MVMR using pQTLs of proteins involved in the cis-MVMR analysis in the ANGPLT3 locus as IVs to evaluating the identified causal protein. We selected independent, genome-wide significant variants for proteins and also LDL-C, HDL-C, and TG, using the minimum P-values across as the identifier of P in PLINK. The selection method for independent IVs was C+T (–clump-kb 1000 –clump-p1 5e-8 –clump-p2 5e-8 –clump-r2 0.01 using PLINK), with LD reference panels consisting of the 9,680 individuals and 9.3M variants from UKBB.

5.3 C+T in UVMR analysis using genome-wide significant IVs

In the UVMR analysis, we performed both protein-to-protein and protein-to-traits analyses. In this case, IVs were selected solely based on their significance for the exposure. For instance, the genome-wide independent significant variants for ANGPTL3 were reduced to 78, compared to 224 in the previous analyses. The selection method for independent IVs utilized clumping and thresholding (C+T) with the following parameters: –clump-kb 1000 –clump-p1 5e-8 –clump-p2 5e-8 –clump-r2 0.01, implemented in PLINK. The LD reference panels were derived from 9,680 individuals and 9.3 million variants in the UK Biobank.

5.4 Meta-analysis of pQTL summary data

We examined the plasma pQTL summary data from Sun et al. (2023) and Ferkingstad et al. (2021) If a protein appears in both datasets, we calculate their genetic correlation using LDSC. Only proteins with a genetic correlation exceeding 0.9 are considered for meta-analysis. Due to the extremely strong pQTL effects for many proteins in the pQTL summary data, which exceed R's machine precision, we directly perform meta-analysis using the following formula:

$$Z_{\text{Meta}} = \frac{\sqrt{n_{\text{Sun}}}}{\sqrt{n_{\text{Sun}} + n_{\text{Ferkingstad}}}} Z_{\text{Sun}} + \frac{\sqrt{n_{\text{Ferkingstad}}}}{\sqrt{n_{\text{Sun}} + n_{\text{Ferkingstad}}}} Z_{\text{Ferkingstad}},$$

where Z represents the Z-score and n represents the sample size.

References

Bowden, J., Davey Smith, G., and Burgess, S. (2015). Mendelian randomization with invalid instruments: effect estimation and bias detection through Egger regression. Int. J. Epidemiol., 44(2), pp. 512–525.

Bowden, J., Davey Smith, G., Haycock, P. C., & Burgess, S. (2016). Consistent estimation in Mendelian randomization with some invalid instruments using a weighted median estimator. Geneti. Epidemiol., 40(4), pp. 304–314.

Breheny, P. and Huang, J. (2011). Coordinate descent algorithms for nonconvex penalized regression, with applications to biological feature selection. The Annals of Applied Statistics, pp 232–253.

Boyd, S., Parikh, N., Chu, E., Peleato, B., Eckstein, J., et al. (2011). Distributed optimization and statistical learning via the alternating direction method of multipliers. Found. Trends Mach. Learn. 3(1), pp. 1–122.

Chen, Jiahua, and Chen, Zehua (2012). Extended BIC for small-n-large-P sparse GLM. Stat. Sinica, 555–574. JSTOR.

Cui, R., Elzur, R. A., Kanai, M., Ulirsch, J. C., Weissbrod, O., Daly, M. J., Neale, B. M., Fan, Z., and Finucane, H. K. (2024). Improving fine-mapping by modeling infinitesimal effects. Nat. Genet. 56(1), pp. 162–169.

Dutilleul, P. (1999). The MLE algorithm for the matrix normal distribution. J. Stat. Comput. Simul. 64(2), pp. 105–123.

Fan, J., and Li, R. (2001). Variable selection via nonconcave penalized likelihood and its oracle properties. J. Am. Stat. Assoc. 96(456), pp. 1348–1360.

Fan, Jianqing, Liao, Yuan, and Mincheva, Martina (2013). Large covariance estimation by thresholding principal orthogonal complements. Journal of the Royal Statistical Society Series B: Statistical Methodology, 75(4), 603–680. Oxford University Press.

Fan, J. and Lv, J. (2011). Nonconcave penalized likelihood with NP-dimensionality. IEEE Trans. Inf. Theory, 57(8), pp. 5467–5484.

Ferkingstad, E., Sulem, P., Atlason, B. A., Sveinbjornsson, G., Magnusson, M. I., Styrmisdottir, E. L., Gunnarsdottir, K., Helgason, A., Oddsson, A., Halldorsson, B. V., et al. (2021). Large-scale integration of the plasma proteome with genetics and disease. Nat. Genet. 53(12), pp. 1712–1721.

Gao, B., and Zhou, X. (2024). MESuSiE enables scalable and powerful multi-ancestry fine-mapping of causal variants in genome-wide association studies. Nat. Genet. 56(1), pp. 170–179.

Li, X., Sham, P. C., and Zhang, Y. D. (2024). A Bayesian fine-mapping model using a continuous global-local shrinkage prior with applications in prostate cancer analysis. Am. J. Hum. Genet., 111(2), pp. 213–226.

Lin, Z., and Pan, W. (2024). A robust cis-Mendelian randomization method with application to drug target discovery. Nat. Commun. 15(1), pp. 6072.

Lorincz-Comi, Noah. and Yang, Yihe. and Li, Gen. and Zhu, Xiaofeng. (2024). MRBEE: A bias-corrected multivariable Mendelian Randomization method. Hum. Genet. Genom. Adv..

She, Y., and Owen, A. B. (2011). Outlier detection using nonconvex penalized regression. J. Am. Stat. Assoc. 106(494), pp. 626–639.

Sun, B. B., Chiou, J., Traylor, M., Benner, C., Hsu, Y.-H., Richardson, T. G., Surendran, P., Mahajan, A., Robins, C., Vasquez-Grinnell, S. G., et al. (2023). Plasma proteomic associations with genetics and health in the UK Biobank. Nature 622(7982), pp. 329–338.

Verbanck, M., Chen, C.-Y., Neale, B., & Do, R. (2018). Detection of widespread horizontal pleiotropy in causal relationships inferred from Mendelian randomization between complex traits and diseases. Nat. Genet., 50(5), pp. 693–698.

Wang, G., Sarkar, A., Carbonetto, P., and Stephens, M. (2020). A simple new approach to variable selection in regression, with application to genetic fine mapping. J. R. Stat. Soc. Ser. B Stat. Methodol. 82(5), pp. 1273–1300.

Wu, Y., Kang, H., and Ye, T. (2024). A more credible approach to multivariable Mendelian randomization. arXiv preprint, arXiv:2402.00307. Available at: https://arxiv.org/abs/2402.00307.

Yavorska, O.O. and Burgess, S. (2017). MendelianRandomization: an R package for performing Mendelian randomization analyses using summarized data. International Journal of Epidemiology, pp.1734-1739.

Yang, J., Ferreira, T., Morris, A. P., Medland, S. E., Genetic Investigation of Anthropometric Traits (GIANT) Consortium, and DIAbetes Genetics Replication And Meta-analysis (DIAGRAM) Consortium, et al. (2012). Conditional and joint multiple-SNP analysis of GWAS summary statistics identifies additional variants influencing complex traits. Nat. Genet. 44(4), pp. 369–375.

Yang, Y., Lorincz-Comi, N. J., and Zhu, X. (2024). Uncovering causal gene-tissue pairs and variants: A multivariable TWAS method controlling for infinitesimal effects. medRxiv, pp. 2024–11.

Yang, Y., Lorincz-Comi, N., and Zhu, X. (2024). Estimation of a genetic Gaussian network using GWAS summary data. Biometrics 80(4), pp. ujae148.

Yi, G. (2016). Statistical analysis with measurement error or misclassification. Springer.

Yuan, Z., Liu, L., Guo, P., Yan, R., Xue, F., and Zhou, X. (2022). Likelihood-based Mendelian randomization analysis with automated instrument selection and horizontal pleiotropic modeling. Sci. Adv. 8(9), pp. eabl5744.

Zhang, C.-H. (2010). Nearly unbiased variable selection under minimax concave penalty. Ann. Stat. pp. 894–942.

Zhao, P. and Yu, B. (2006). On model selection consistency of Lasso. J. Mach. Learn. Res., 7(Nov), pp. 2541–2563.

Zhu, X., and Stephens, M. (2017). Bayesian large-scale multiple regression with summary statistics from genome-wide association studies. Ann. Appl. Stat. 11(3), pp. 1561.

6 Supplementary Figures

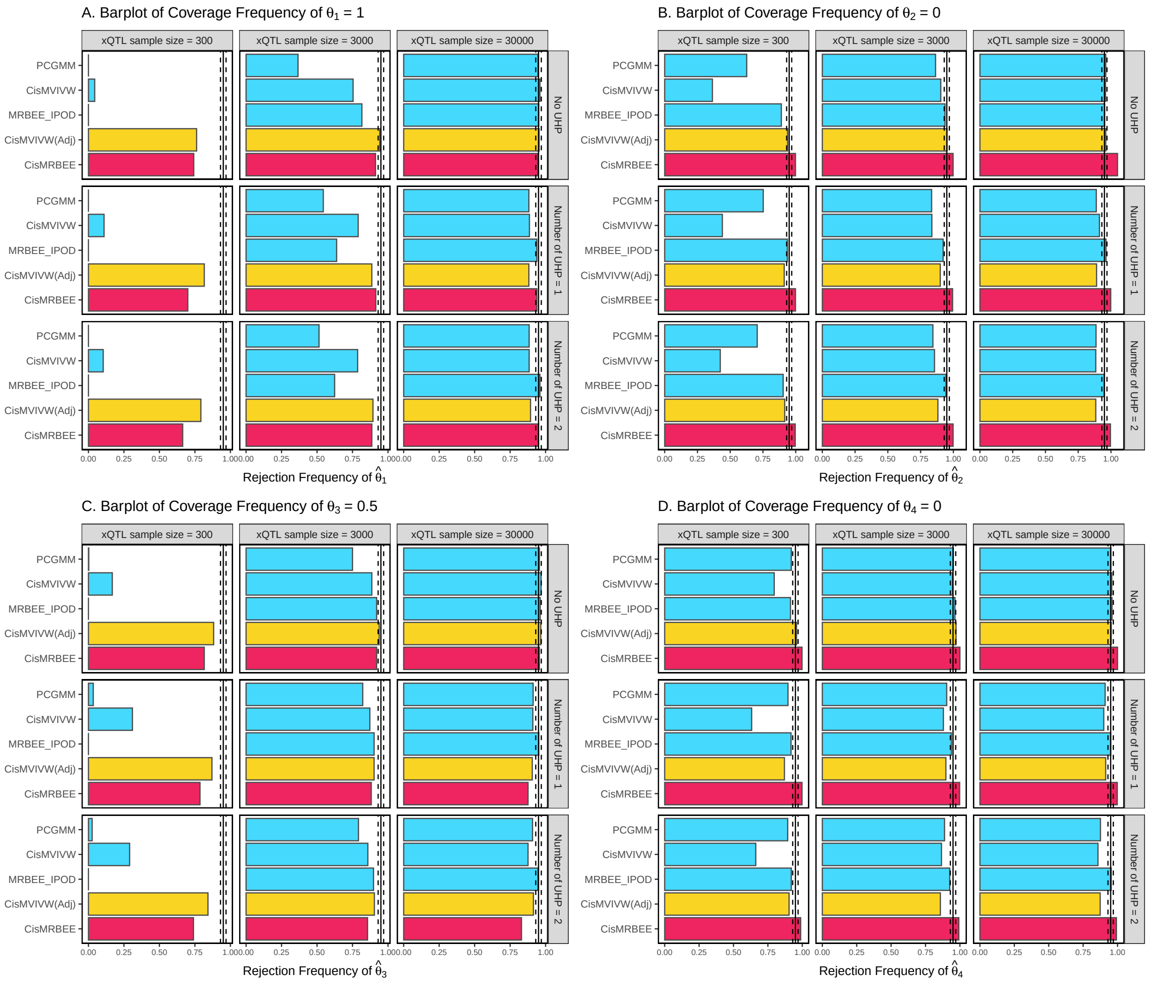

Figure S1: This figure illustrates the coverage frequencies of $\hat{\theta}_1$ to $\hat{\theta}_4$ across different methods, when the number of causal xQTL of exposures equal to **3**. TGVIS and TGFM are not included as they do not involve traditional hypothesis testing. The results show: (1) When the sample size $n_{\text{xQTL}} = 300$, only cis-MRBEE and cis-MVIVW (Adjusted) achieve a coverage frequency of 75%-85%. (2) As the sample size increases, the coverage frequency improves across all methods. (3) For $\hat{\theta}_3$, MRBEE-IPOD maintains a coverage frequency close to 95% when $n_{\text{xQTL}} = 30000$, while cis-MRBEE achieves approximately 90%. Since the primary difference between MRBEE-IPOD and cis-MRBEE is the use of SuSiE to select informative variants, we hypothesize that the uncertainty introduced during this selection process is not fully captured by cis-MRBEE's covariance estimation, resulting in a slightly lower coverage frequency.

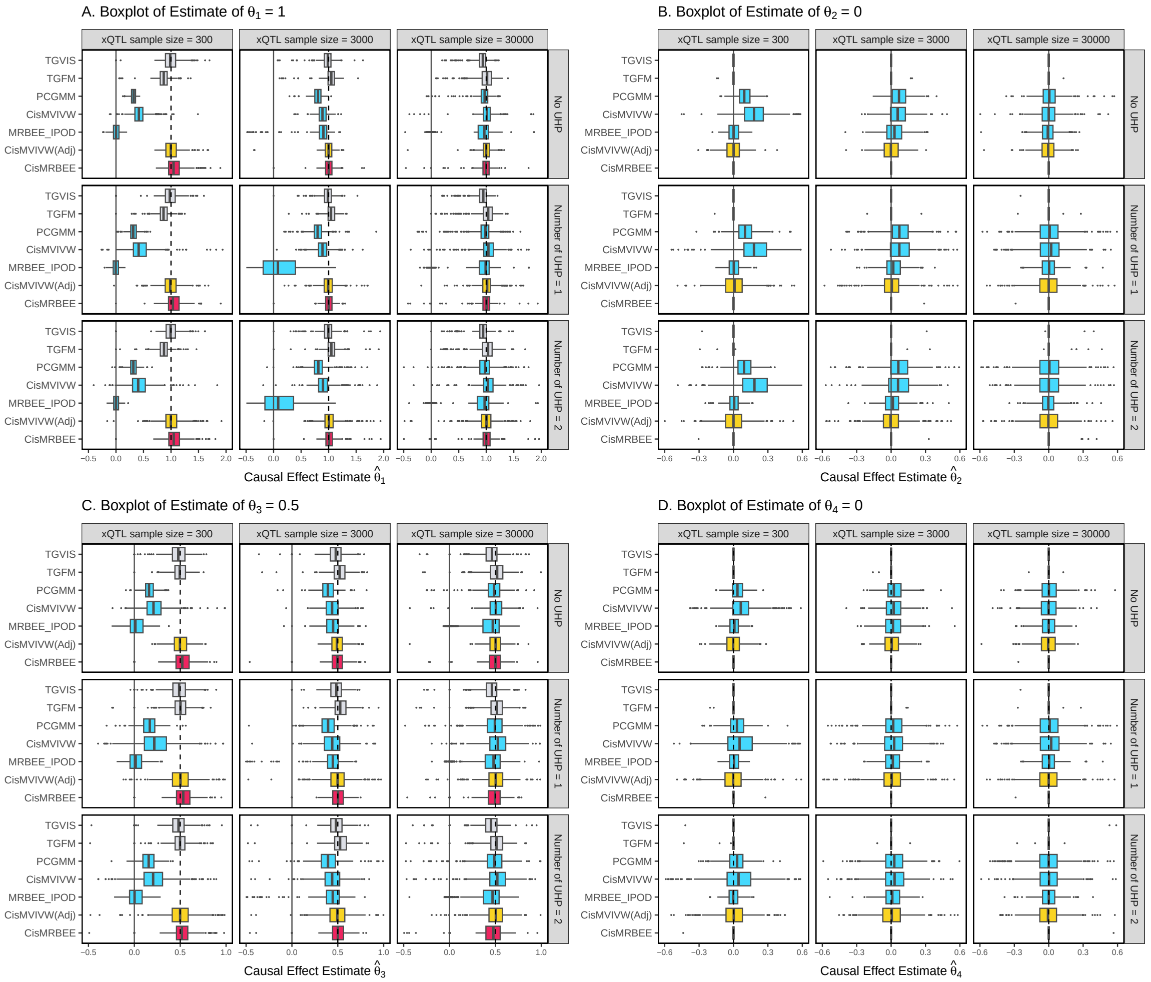

Figure S2: This figure illustrates the boxplots of $\hat{\theta}_1$ to $\hat{\theta}_4$ across different methods when the number of causal xQTLs for exposures is **1**. The conclusions are similar to those presented in the main text for the case where the number of causal xQTLs equals **3**. Specifically: when $n_{\text{xQTL}} = 300$, only cis-MRBEE and TGVIS provide unbiased estimates, while cis-MVIVW (Adjusted) and TGFM exhibit minor biases, and the other methods yield entirely unreliable estimates. As n_{xQTL} increases, cis-MRBEE visibly outperforms the other methods. However, TGVIS, which accounts for infinitesimal effects, shows bias in the estimation of $\hat{\theta}_3$.

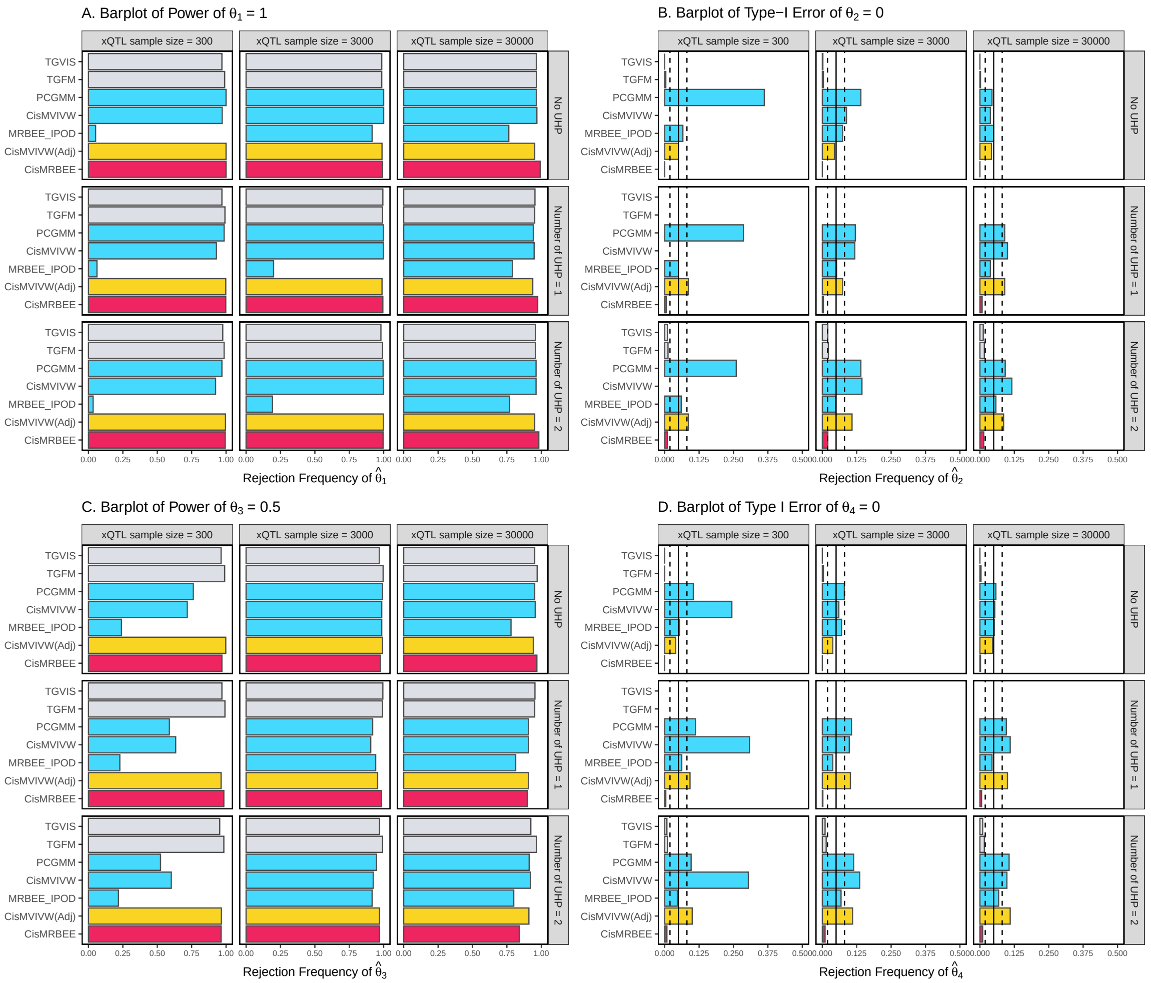

Figure S3: This figure illustrates the barplots of the powers/type-I errors of $\hat{\theta}_1$ to $\hat{\theta}_4$ across different methods when the number of causal xQTLs for exposures is **1**. The conclusions are similar to those presented in the main text for the case where the number of causal xQTLs equals **3**.

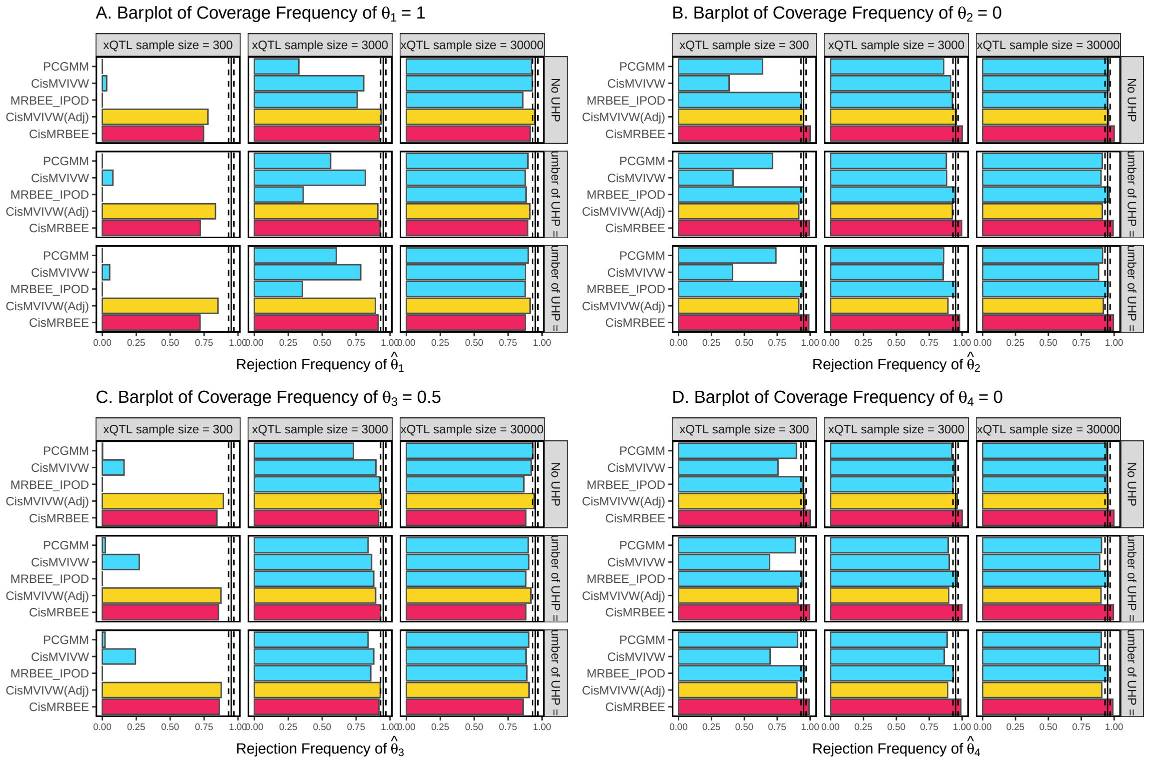

Figure S4: This figure presents the barplots of the coverage frequencies for $\hat{\theta}_1$ to $\hat{\theta}_4$ across different methods when the number of causal xQTLs for exposures is **1**. Compared to the scenario where the number of causal xQTLs is **3**, we observe a slight decrease in coverage rates for all methods. This suggests that the reliability of cis-MVMR methods is influenced by the underlying number of causal or informative variants in each locus. Ideally, a higher number of causal variants enhances the reliability of causal effect estimation.

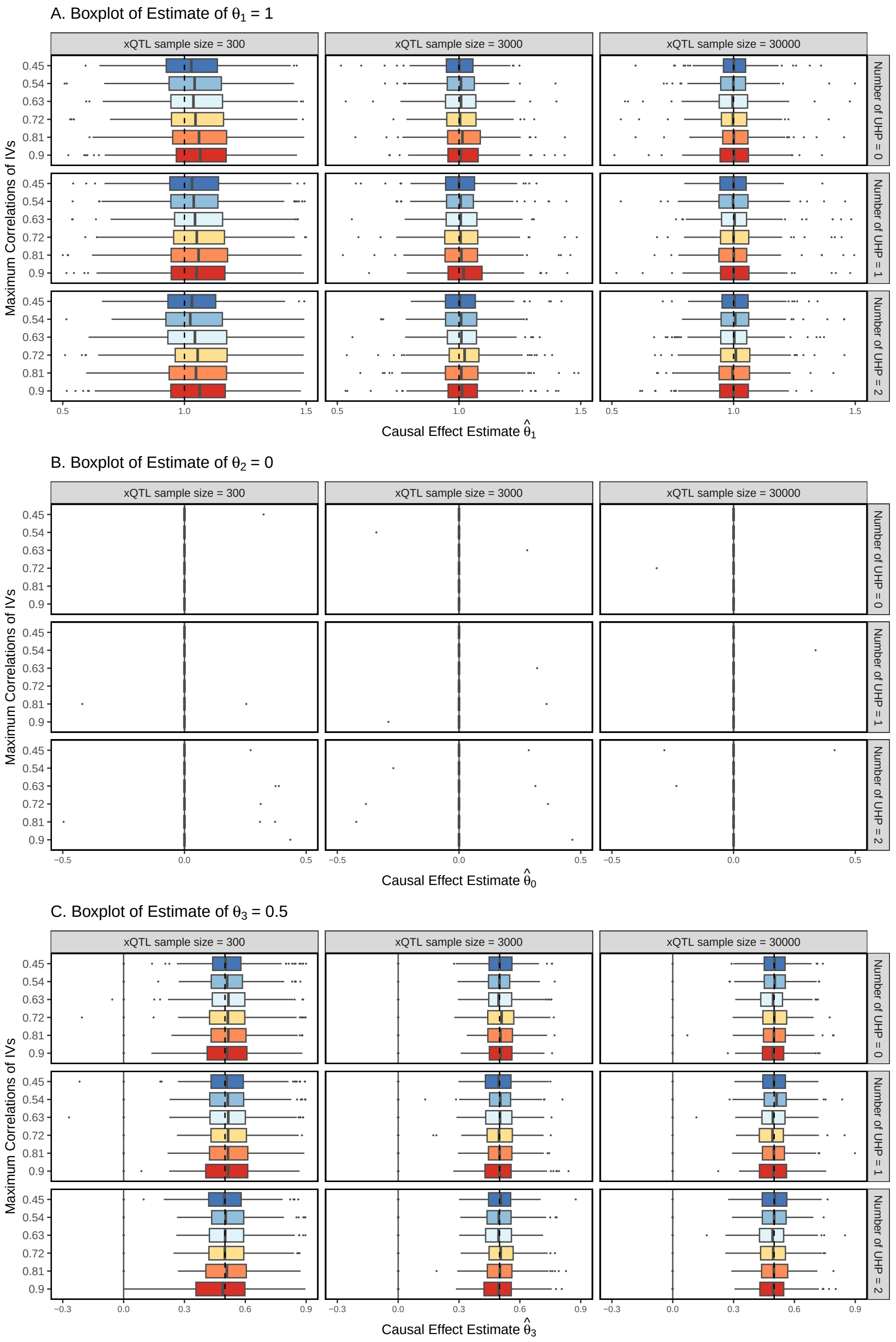

Figure S5: This figure presents the boxplots of $\hat{\theta}_1$ to $\hat{\theta}_3$ estimated by cis-MRBEE as the maximum correlation coefficient in the LD matrix decreases. For $\hat{\theta}_1$, which has a larger effect size, the estimates remain consistent across different correlation coefficients. For $\hat{\theta}_2$, cis-MRBEE effectively shrinks the estimates to zero under all correlation conditions. For $\hat{\theta}_3$, slight instability is observed only when the maximum correlation coefficient is 0.9. The number of causal xQTLs for exposures is **3**.

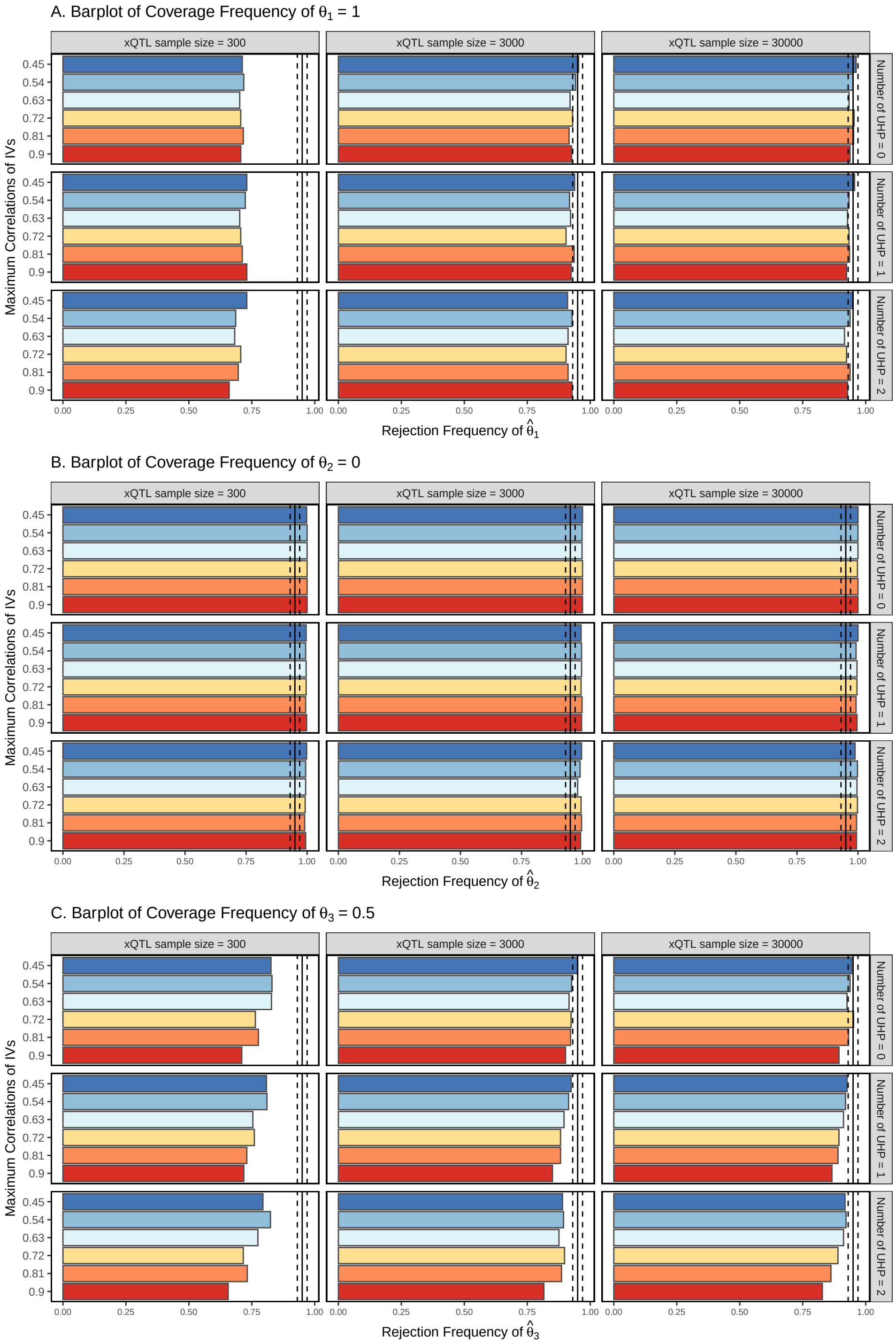

Figure S6: This figure shows the barplots of coverage frequency for $\hat{\theta}_1$ to $\hat{\theta}_3$ estimated by cis-MRBEE as the maximum correlation coefficient in the LD matrix decreases. We observed a noticeably lower coverage frequency when the maximum correlation is 0.9, particularly in models with two horizontal pleiotropy terms. Although the coverage frequency increases smoothly as the maximum correlation decreases, using a lower r^2 threshold in PLINK may inadvertently remove too many causal and informative variants, posing a more significant issue. Therefore, we recommend an r^2 threshold of 0.64 in practical applications, corresponding to a maximum correlation coefficient of 0.8. The number of causal xQTLs for exposures is **3**.

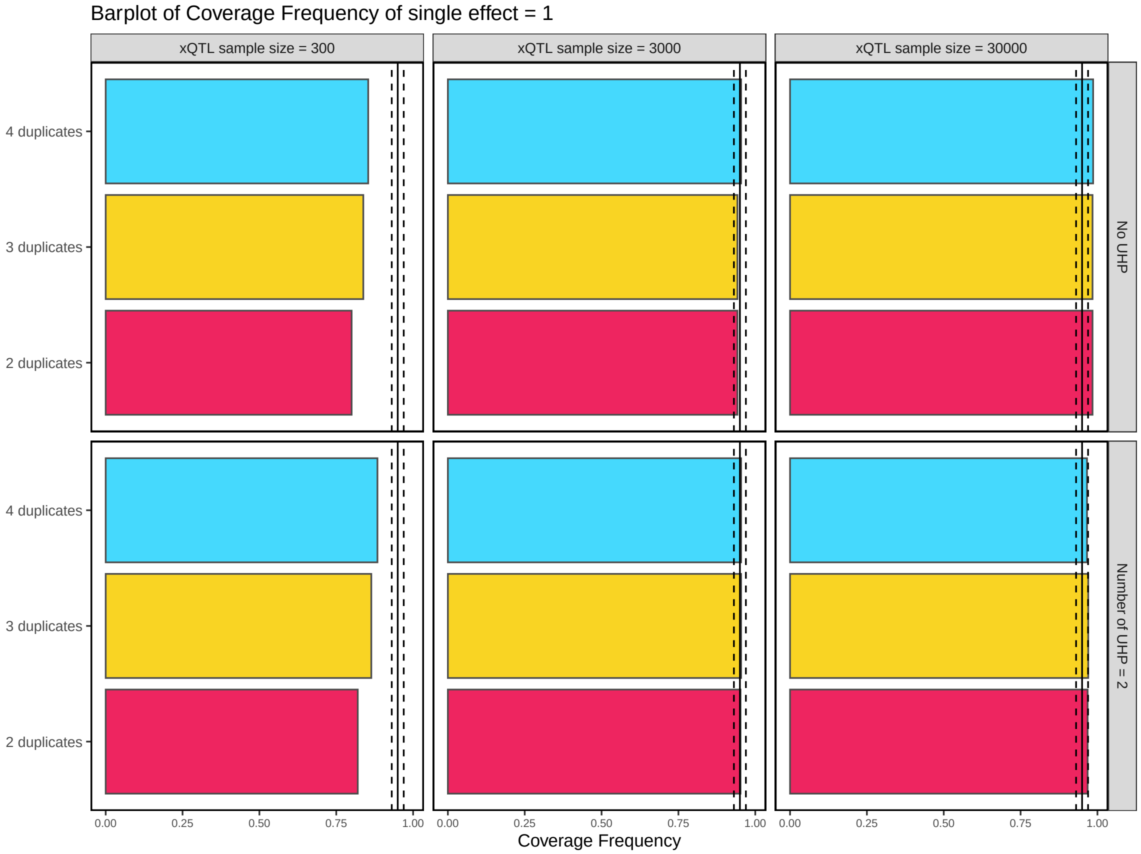

Figure S7: This figure illustrates the coverage frequency of single effects constructed from all exposures within a credible set when duplicates of exposures exist in the model. The single effect can be understood as a meta-analysis-like aggregation of variables within the credible set, treated as a single variable in the cis-MVMR analysis. This approach effectively increases the sample size for exposures. As a result, we observed improved coverage: even at $n_{\mathrm{eQTL}} = 3000$, the coverage frequency maintained 95%. However, when $n_{\mathrm{eQTL}} = 300$, the coverage frequency averaged only around 85%.

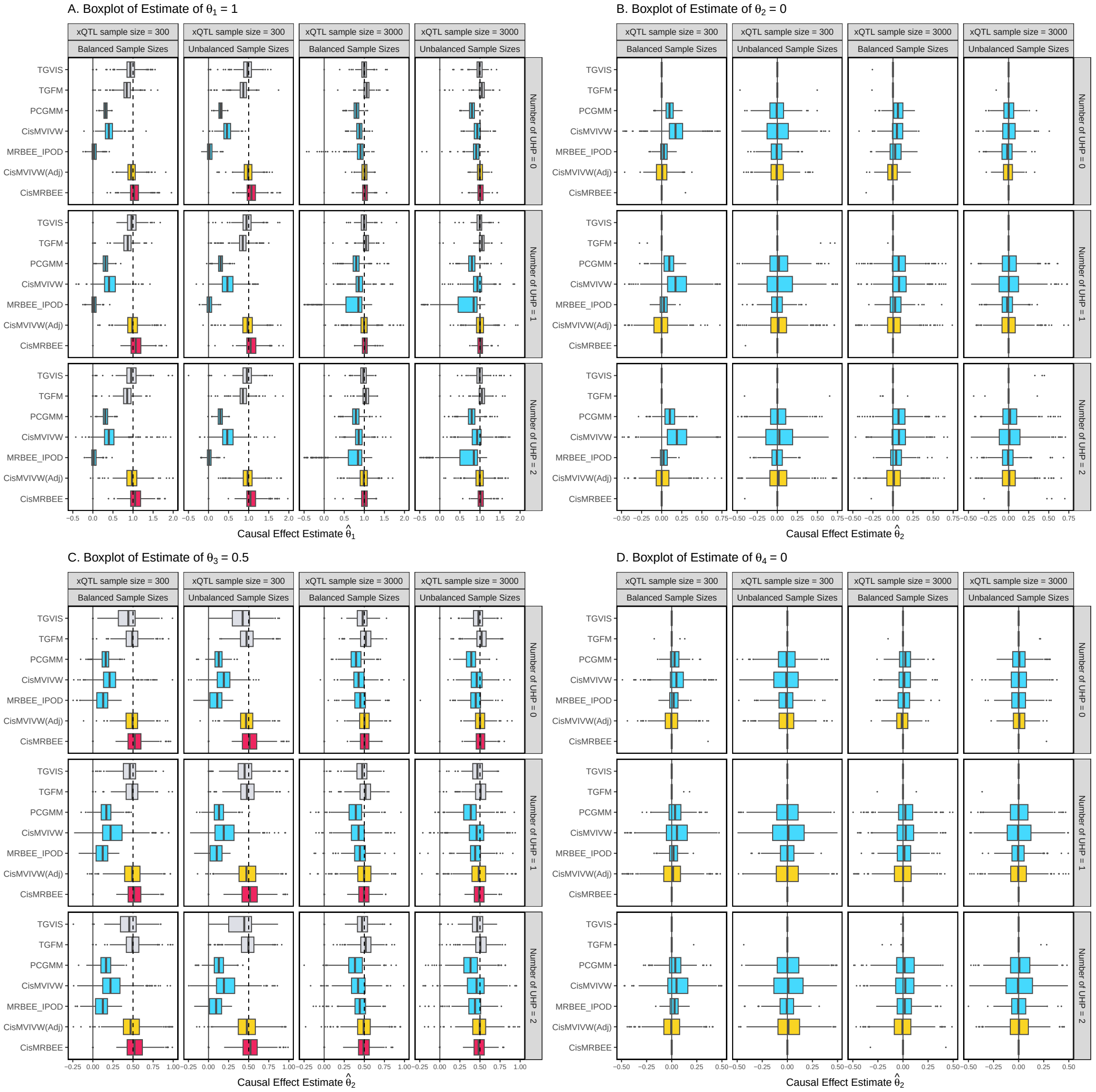

Figure S8: This figure illustrates the performance of various methods when the sample sizes of exposures are unbalanced, with the number of causal xQTLs set to 3. Specifically, the sample sizes for the second and fourth exposures were fixed at 30,000, while the sample sizes for the other exposures varied between 300 and 30,000. These exposures were chosen because they exhibited higher correlations with the causal exposures. The results show that cis-MRBEE did not overemphasize exposures with larger sample sizes, maintaining balanced power across exposures.

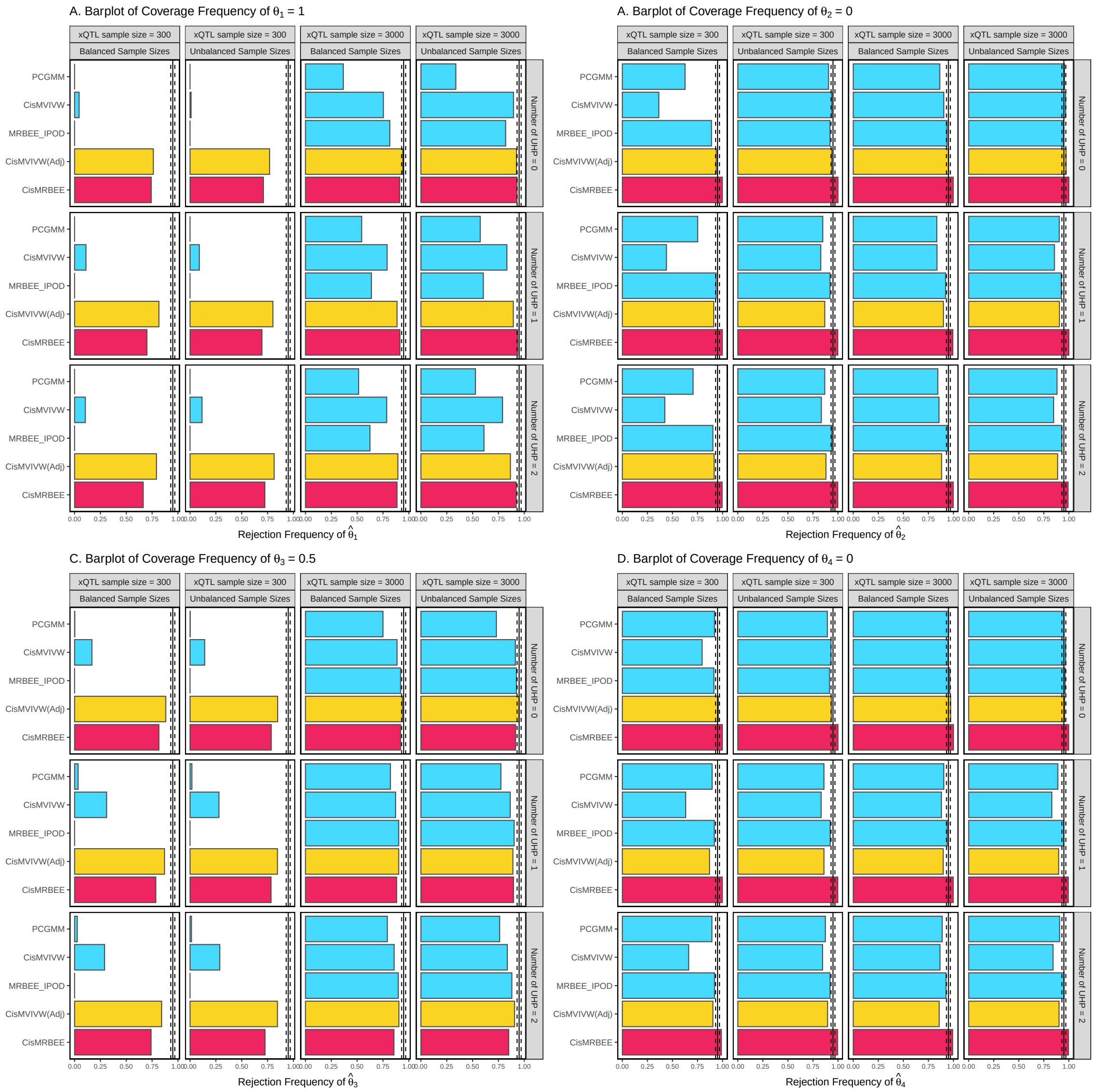

Figure S9: This figure further investigates the coverage frequency of various methods. Compared to scenarios with balanced sample sizes, unbalanced sample sizes did not impact the coverage frequency of cis-MRBEE, demonstrating its robustness under such conditions.

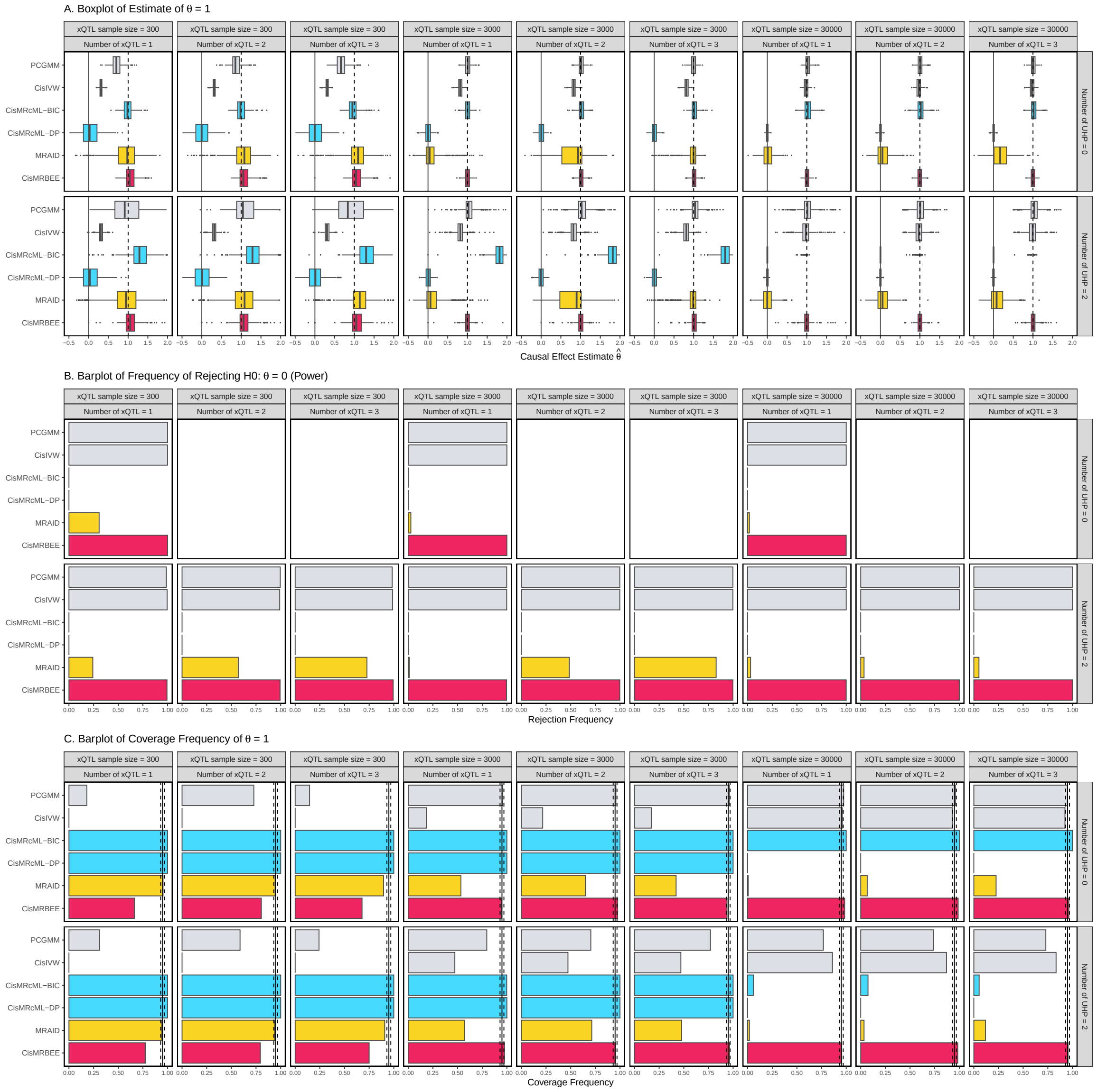

Figure S10: This figure illustrates the boxplots of causal effect estimates, power barplots, and coverage frequency barplots under the cis-UVMR setting across various methods. The true causal effect $\theta = 1$. A notable anomaly is that, as n_{xQTL} increases, the performance of MRAID declines, resulting in causal effect estimates close to 0, particularly when the number of causal xQTLs for exposures is **1**. We hypothesize that when the number of causal xQTLs is very small, MRAID tends to degenerate into a fine-mapping method, misidentifying both horizontal pleiotropy of the outcome and causal xQTLs of the exposure as outcome causal variants. A similar issue occurs with MRcML-BIC, which also misclassifies horizontal pleiotropy and causal xQTLs as causal variants of the outcome in the presence of horizontal pleiotropy. MRcML-DP, on the other hand, consistently yields $\hat{\theta} = 0$, likely due to the failure of the permutation step under our model settings. Notably, under the cis-UVMR setting, cis-MRBEE demonstrates improved coverage frequency compared to its performance in cis-MVMR.

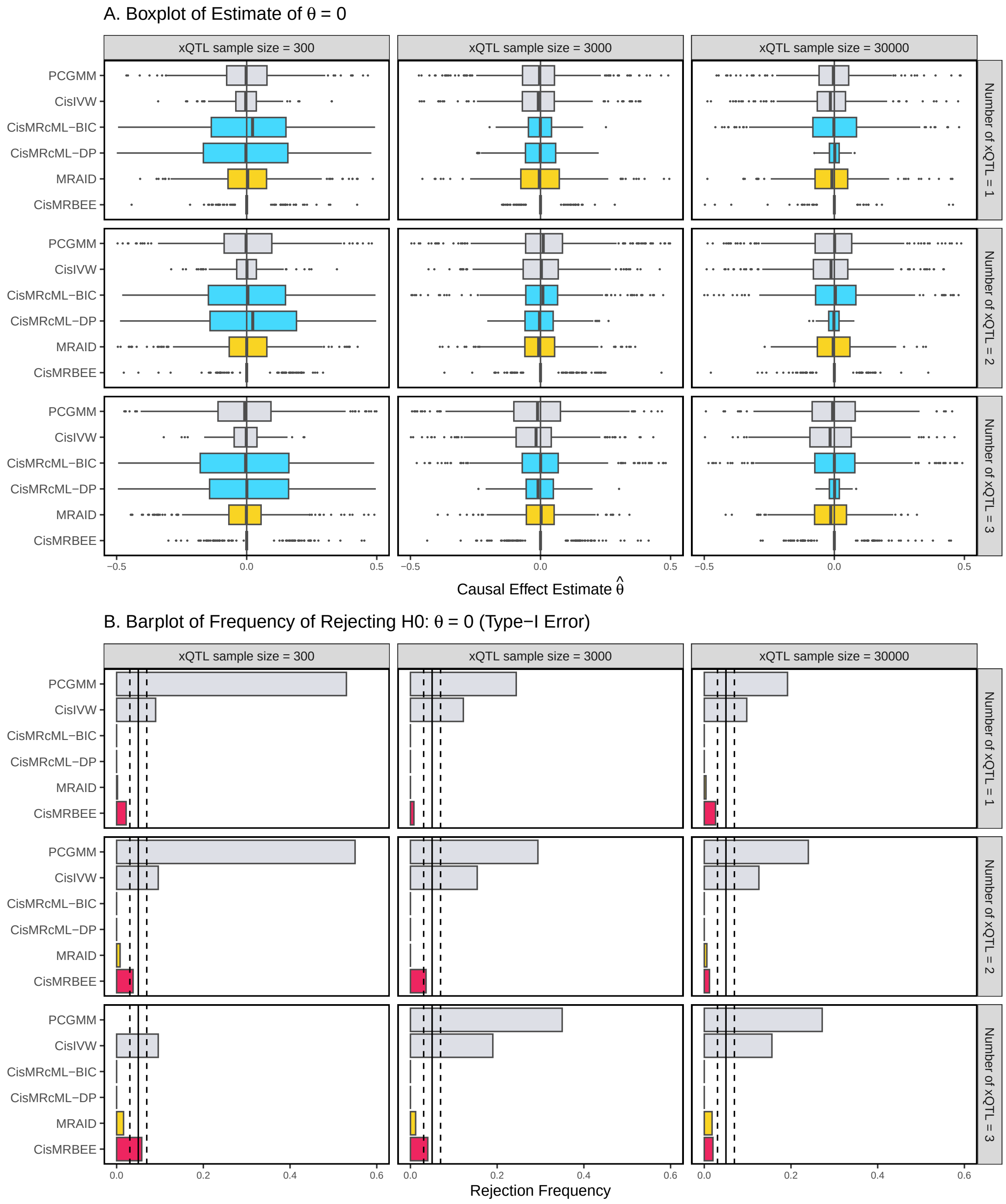

Figure S11: This figure illustrates the boxplots of causal effect estimates and type-I error barplots under the cis-UVMR setting across various methods, where the true causal effect is $\theta = 0$. Traditional methods, such as PCGMM and cis-IVW, continue to exhibit significantly inflated type-I error rates.

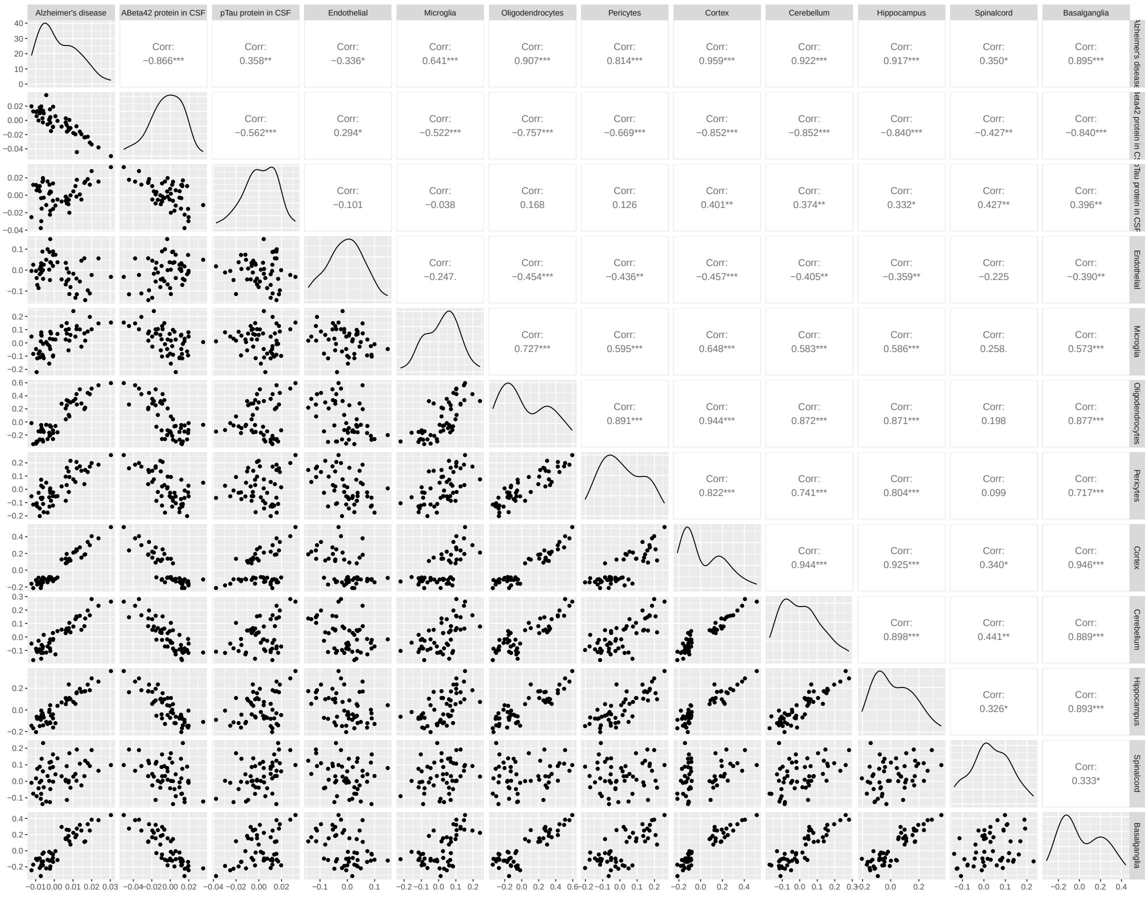

Figure S12: This figure presents the counterpart of Figure 2A in the main text, showing scatter plots of the effect sizes of IVs used in cis-MVMR for AD, Aβ42, and pTau against $CR1$-tissue/cell-type pairs at the CR1 locus.

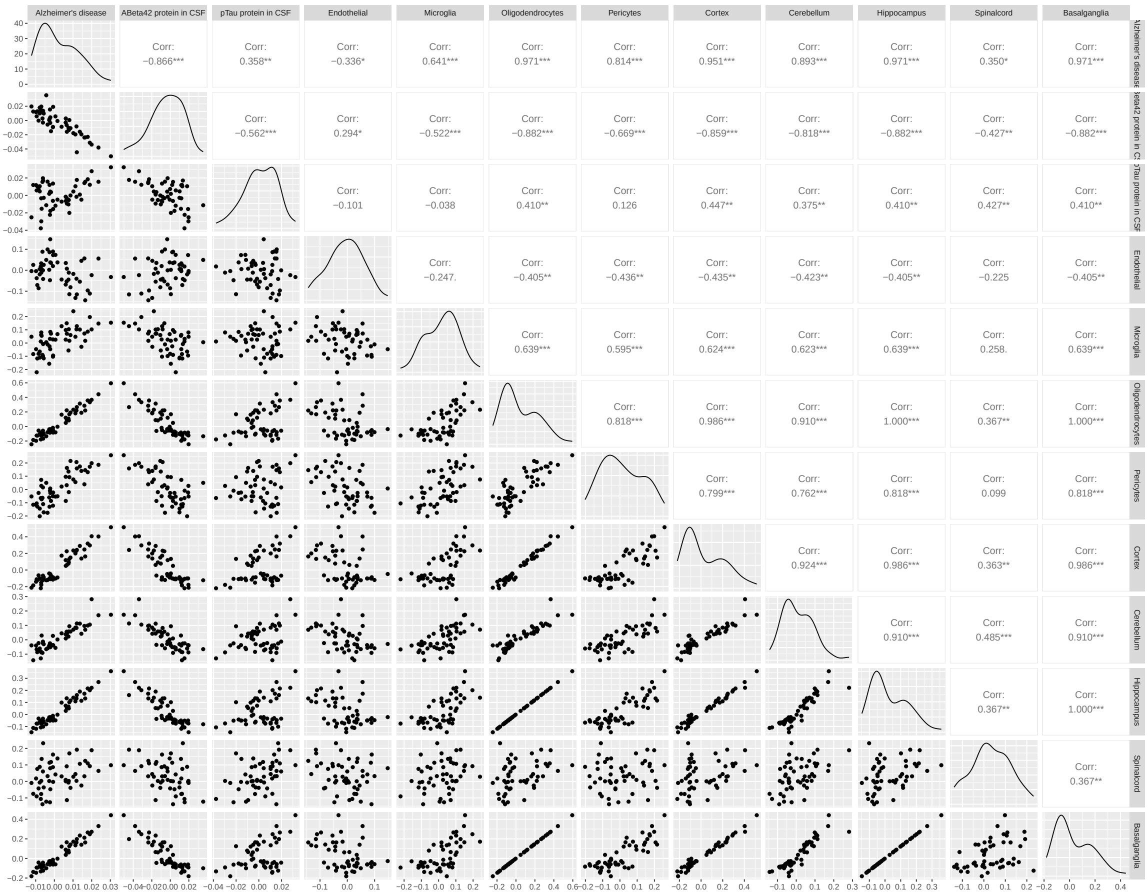

Figure S13: This figure presents the counterpart of Figure 2B in the main text, showing scatter plots of the effect sizes of IVs used in cis-MVMR for AD, Aβ42, and pTau against the sparsely predicted effect sizes of $CR1$-tissue/cell-type pairs at the CR1 locus.

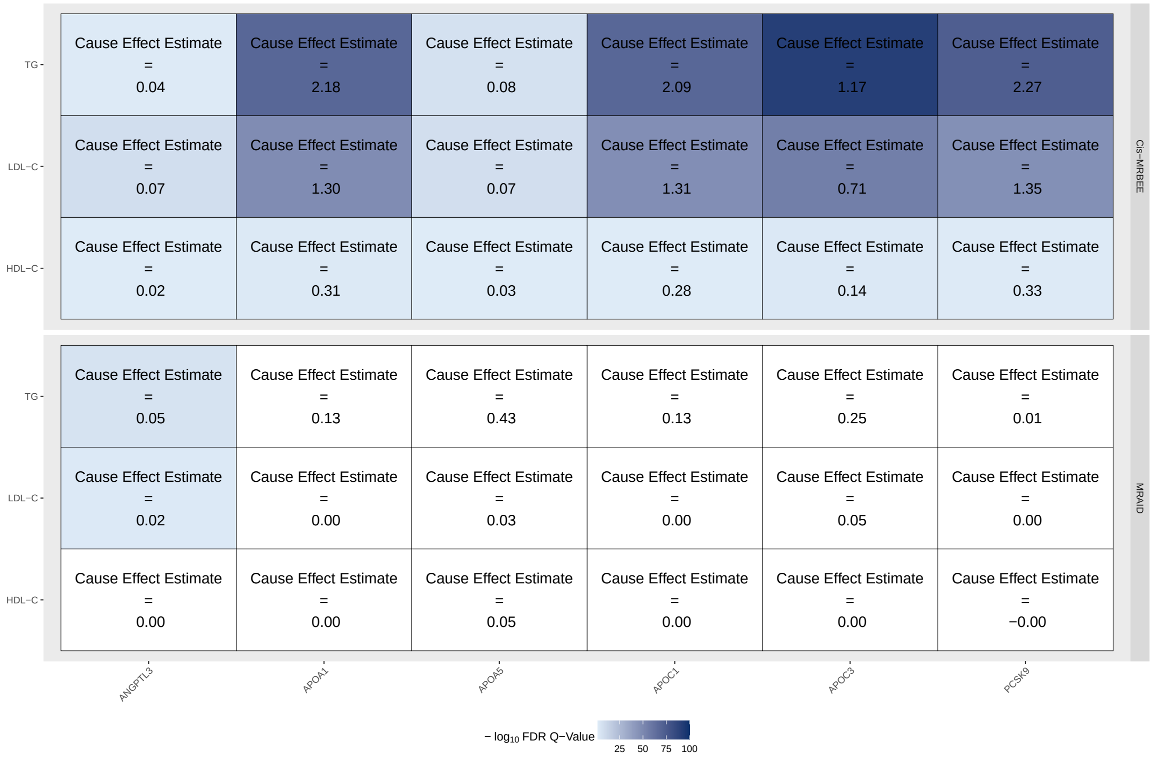

Figure S14: This figure illustrates the cis-UVMR estimates of protein-to-lipid causal effects provided by cis-MRBEE and MRAID at the ANGPTL3 locus. Heatmap of cis-UVMR causal effects (Estimate) between exposures and outcomes using two methods. Each cell displays the Estimate value, with color intensity representing the -$\log_{10}$(FDR Q-value) for $Q < 0.05$ (shaded from light to dark blue using the Blues palette; $Q \geq 0.05$, white). The x-axis lists exposures, the y-axis lists outcomes, and panels separate the two methods.

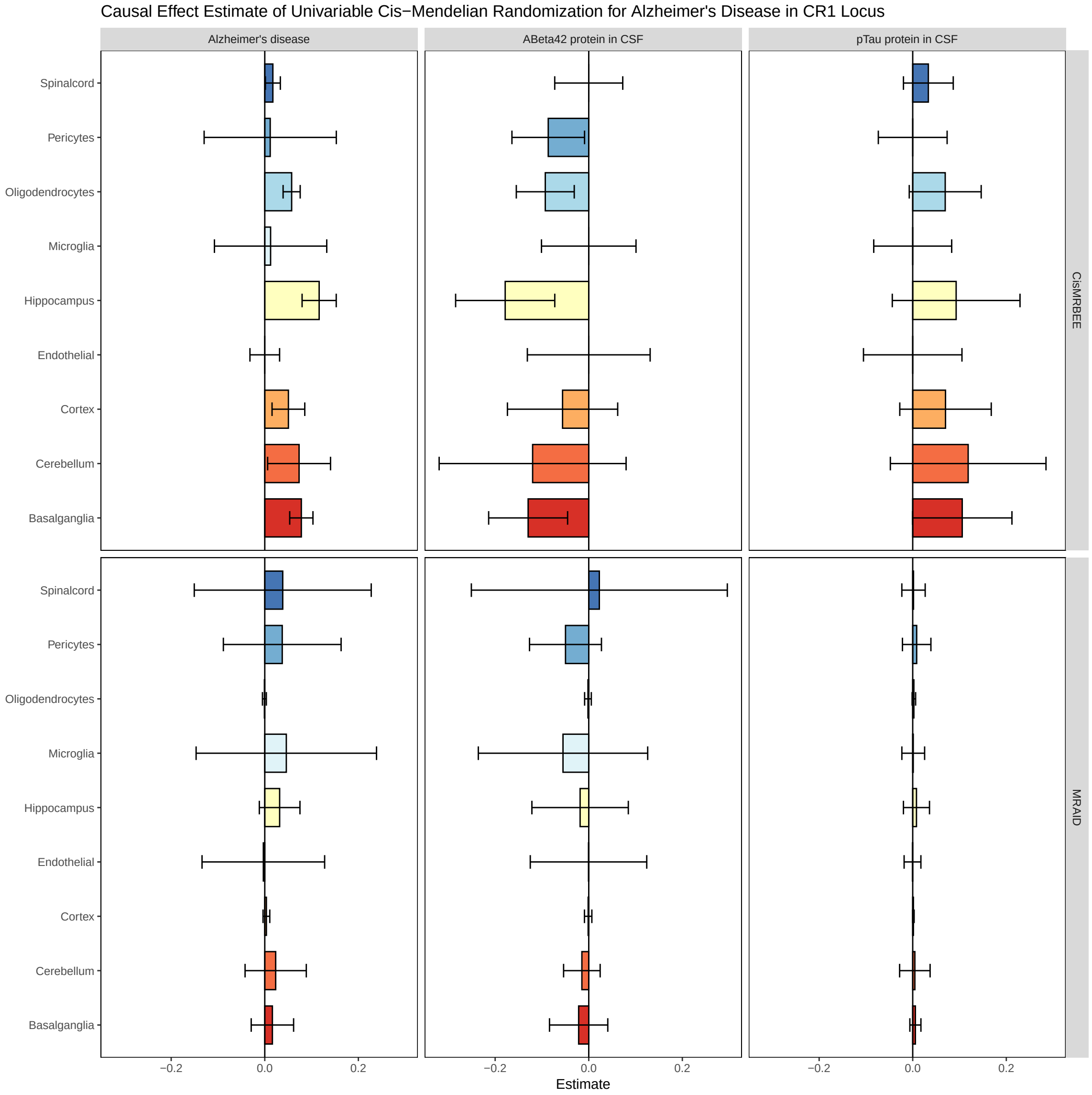

Figure S15: This figure illustrates the cis-UVMR estimates of protein-to-AD/biomarkers causal effects provided by cis-MRBEE and MRAID at the CR1 locus.

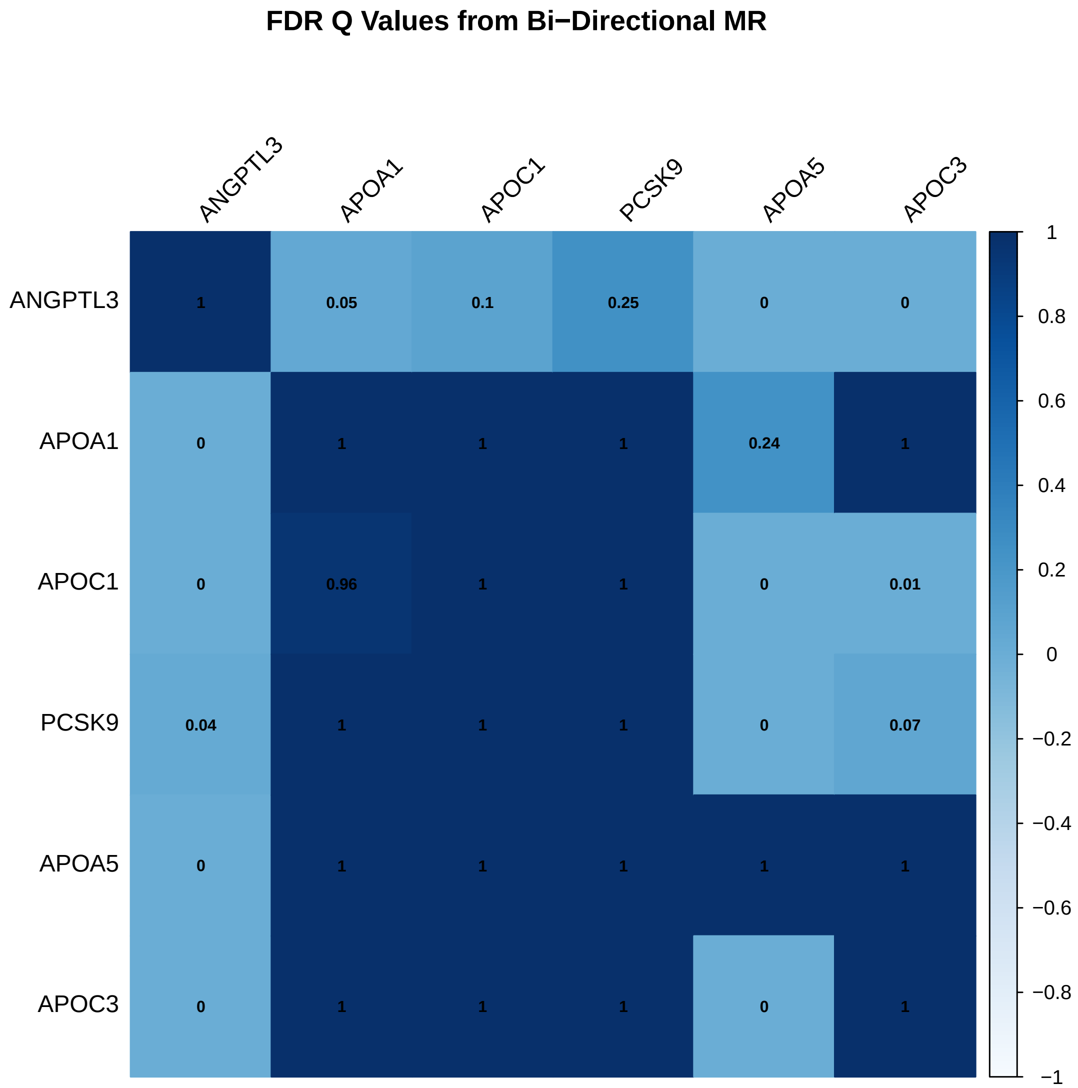

Figure S16: This figure illustrates FDR Q-values of bidirectional cis-UVMR analysis. The columns represent the exposure, while the rows represent the outcome. For example, the Q-value at the matrix position (2,1) is 0, indicating that the Q-value for the causal effect of ANGPTL3 to APOA1 is 0.

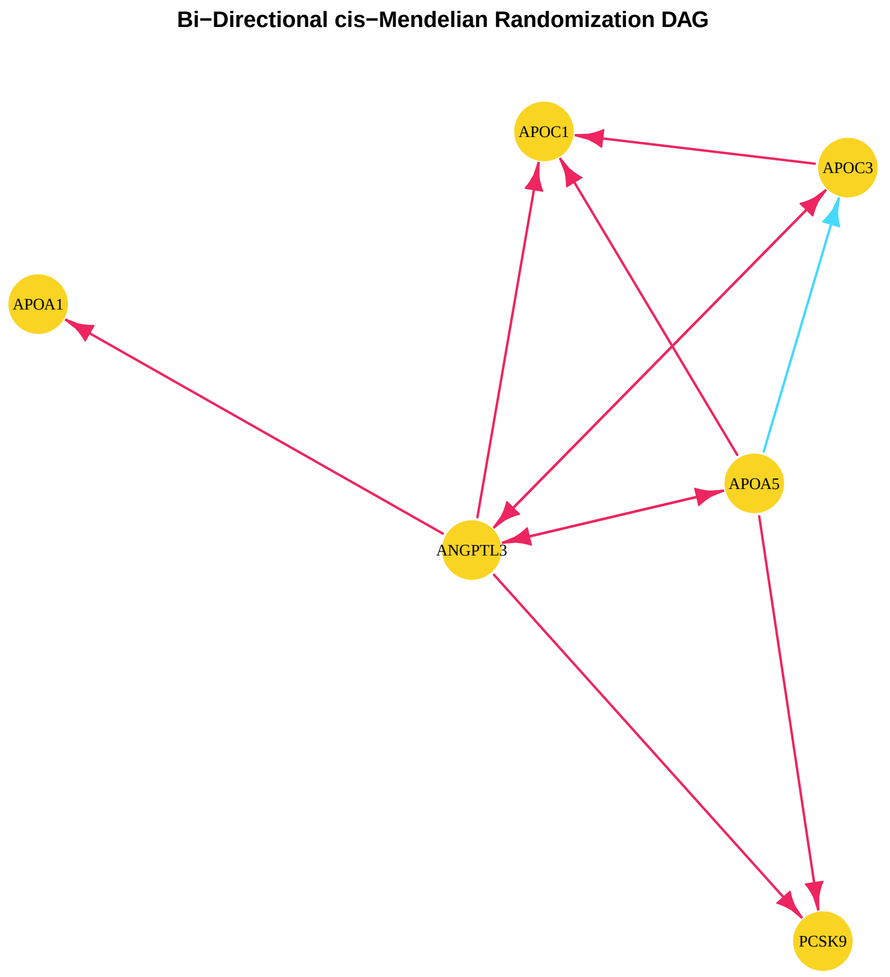

Figure S17: This diagram represents a DAG constructed based on cis-UVMR MRBEE results. For instance, a significant UVMR result from ANGPLT3 to PCSK9 is represented by an arrow pointing from ANGPLT3 to PCSK9. Red and blue arrows refer to positive and negative direction.

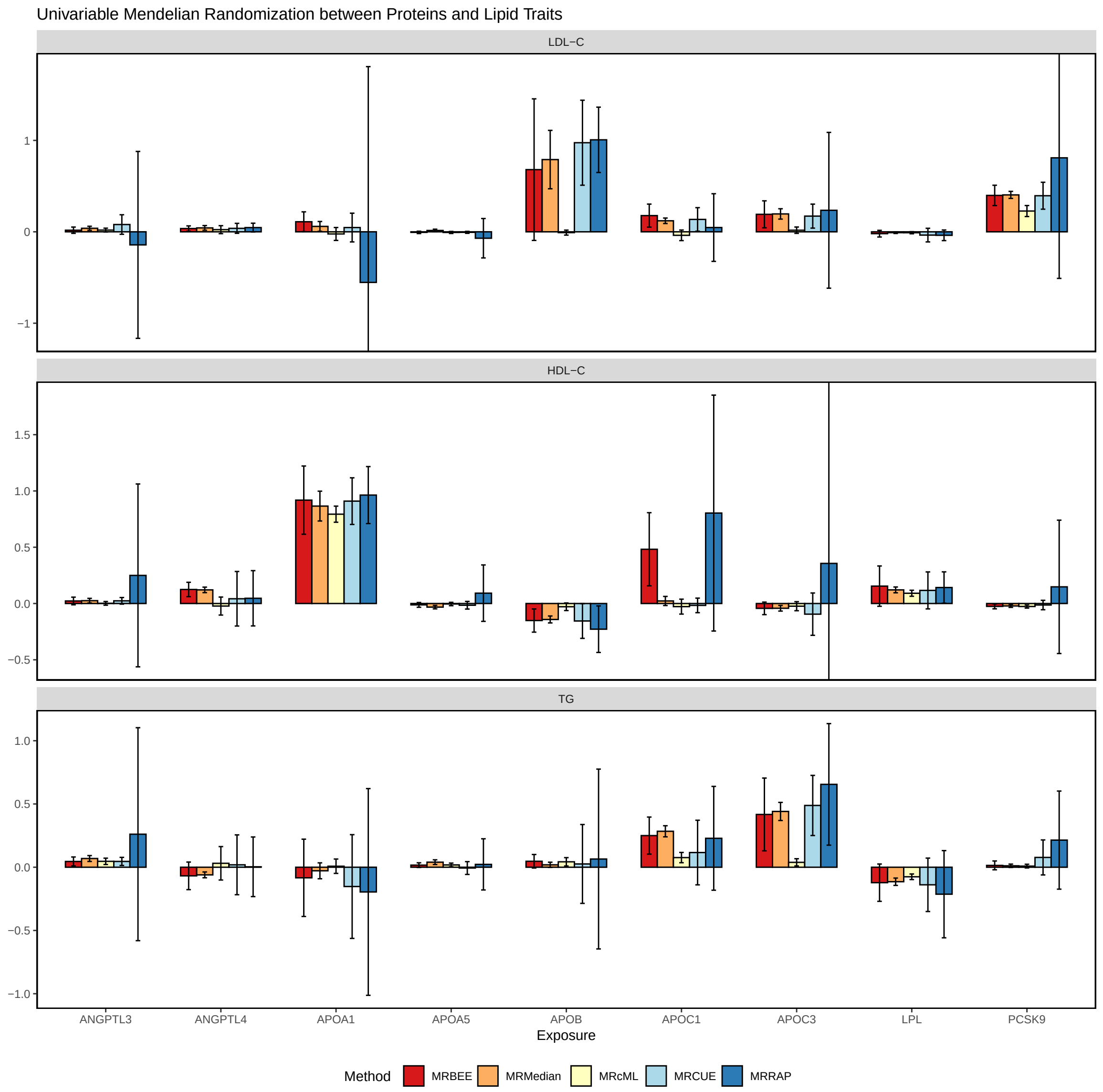

Figure S18: This figure illustrates the UVMR estimates of protein-to-lipids causal effects provided by MRBEE, MR-Median, MRcML, MRCUE, and MR-RAPS using the genome-wide significant variants as IVs. Confidence intervals were calculated while accounting for Bonferroni correction.

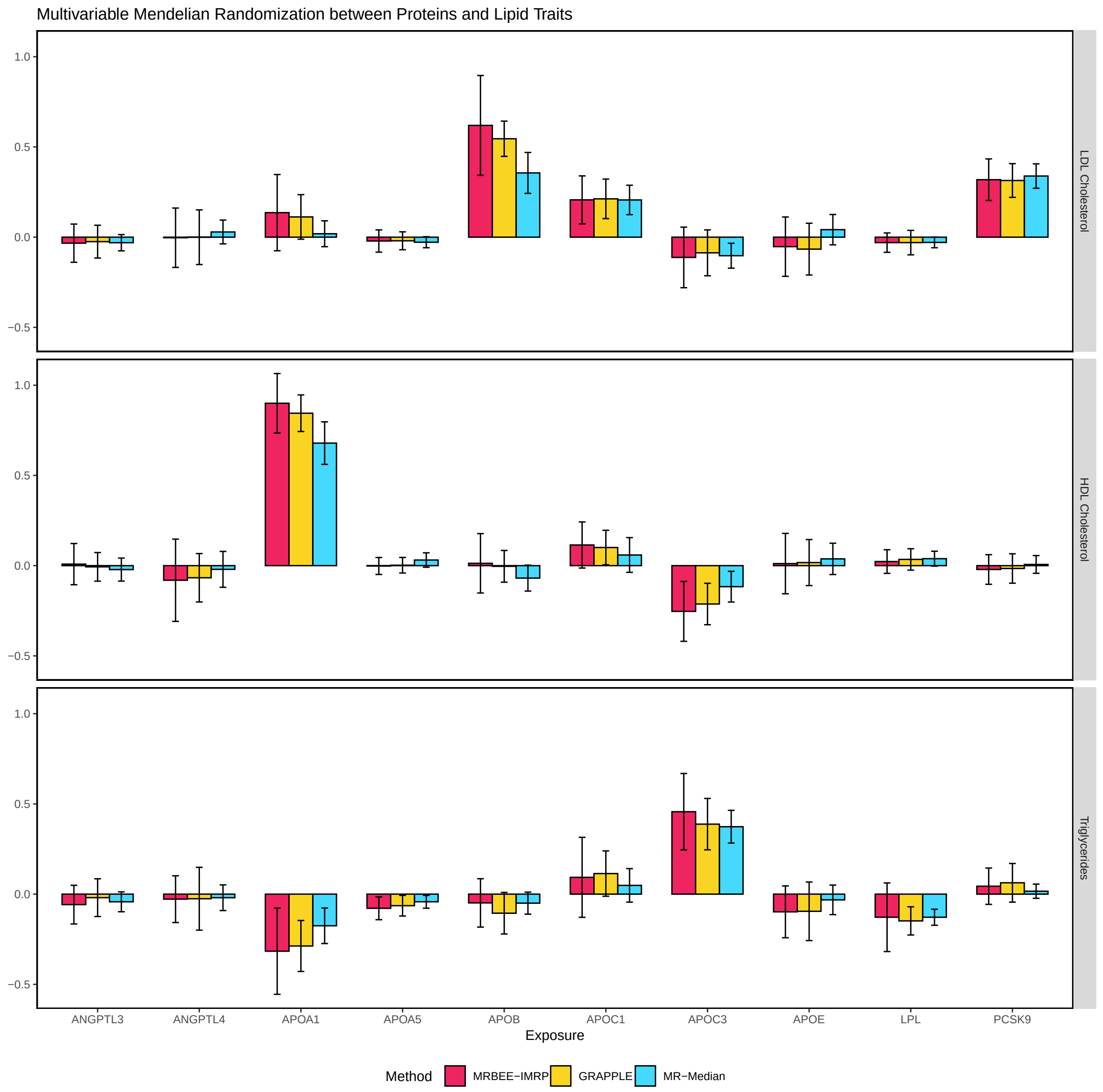

Figure S19: This figure illustrates the MVMR estimates of protein-to-lipids causal effects provided by MRBEE, GRAPPLE, and MR-Median using the genome-wide significant variants as IVs. Confidence intervals were calculated while accounting for Bonferroni correction.

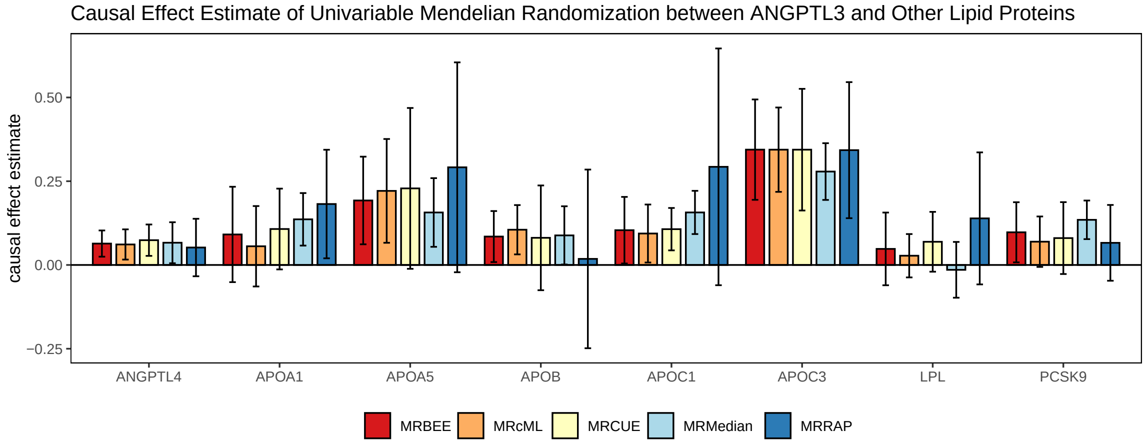

Figure S20: We conducted bi-directional Mendelian randomization between proteins, using independent variants with P < 5E-8 as IVs. This figure presents the results with ANGPLT3 as the exposure and other proteins as the outcomes. Confidence intervals were calculated while accounting for Bonferroni correction.

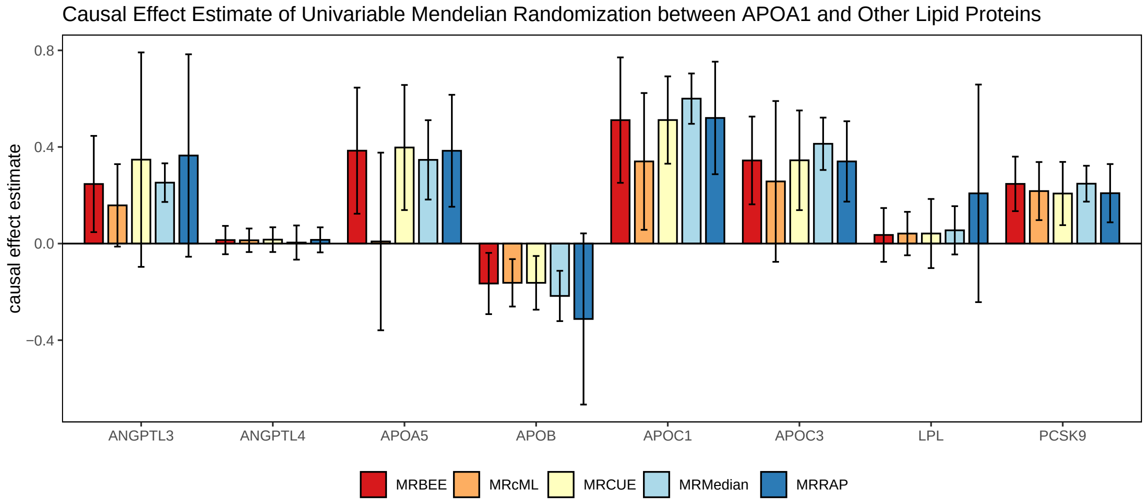

Figure S21: We conducted bi-directional Mendelian randomization between proteins, using independent variants with P < 5E-8 as IVs. This figure presents the results with APOA1 as the exposure and other proteins as the outcomes. Confidence intervals were calculated while accounting for Bonferroni correction.

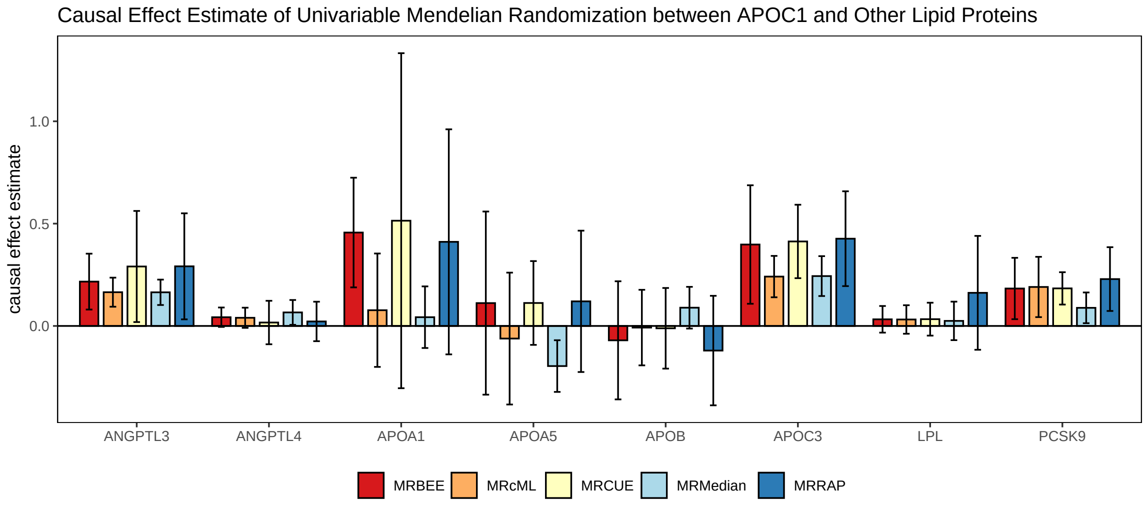

Figure S22: We conducted bi-directional Mendelian randomization between proteins, using independent variants with P < 5E-8 as IVs. This figure presents the results with APOC1 as the exposure and other proteins as the outcomes. Confidence intervals were calculated while accounting for Bonferroni correction.

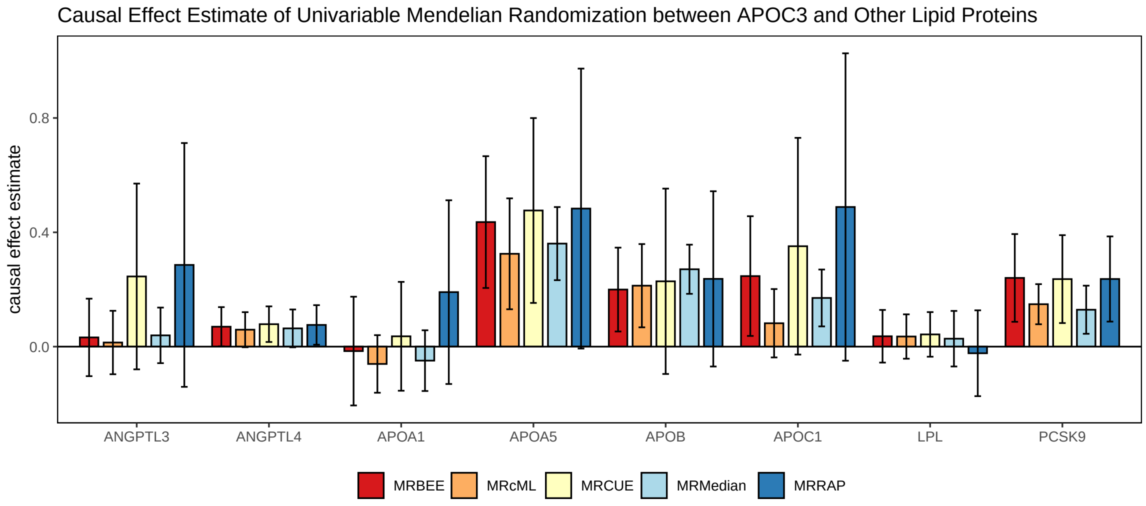

Figure S23: We conducted bi-directional Mendelian randomization between proteins, using independent variants with P < 5E-8 as IVs. This figure presents the results with APOC3 as the exposure and other proteins as the outcomes. Confidence intervals were calculated while accounting for Bonferroni correction.

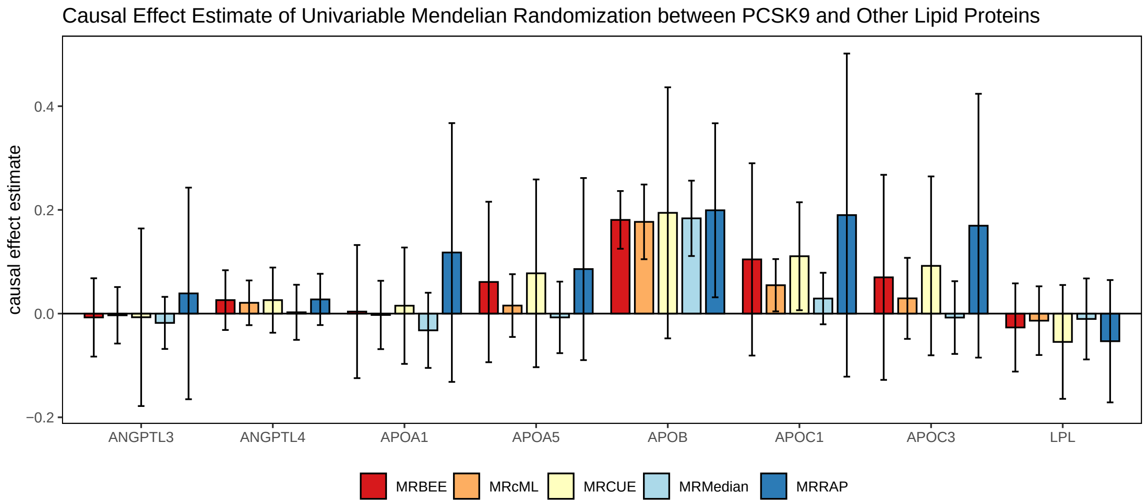

Figure S24: We conducted bi-directional Mendelian randomization between proteins, using independent variants with P < 5E-8 as IVs. This figure presents the results with PCSK9 as the exposure and other proteins as the outcomes. Confidence intervals were calculated while accounting for Bonferroni correction.

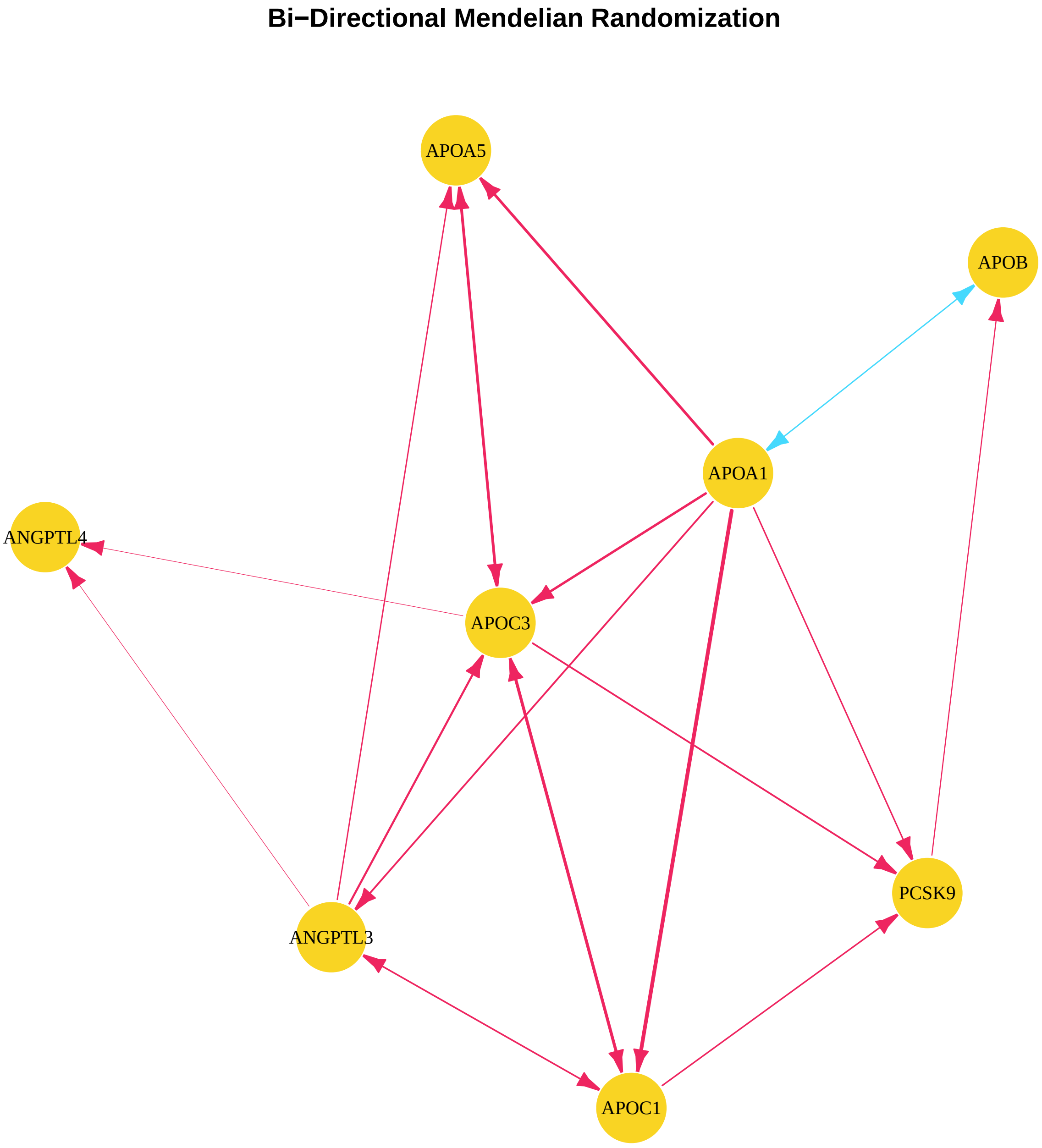

Figure S25: This diagram represents a directed acyclic graph (DAG) constructed based on univariable MRBEE results. For instance, a significant UVMR result from ANGPLT3 to ANGPLT4 is represented by an arrow pointing from ANGPLT3 to ANGPLT4, whereas the reverse direction (ANGPLT3 to ANGPLT4) showed no significant result. Significance was determined using Bonferroni correction. Blue arrows indicate a negative causal direction, while red arrows indicate a positive causal direction.

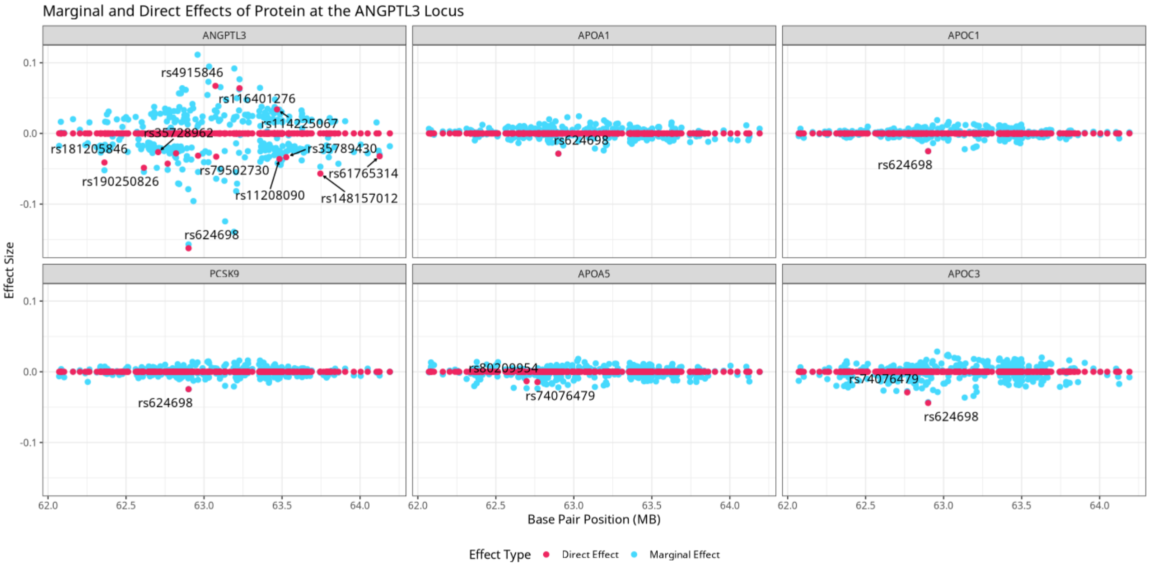

Figure S26: This figure demonstrate the marginal and direct effect estimates of 6 proteins at the ANGPTL3 locus.
